# Supplementary material for: Versican accumulation drives Nos2 induction and aortic disease in Marfan syndrome via Akt activation
Source: EMBO Mol Med. 2024 Jan 2;16(1):9. doi: 10.1038/s44321-023-00009-7 (PMC10897446; doi:10.1038/s44321-023-00009-7)
Supplement: Supplementary file 1 — Appendix [file 44321_2023_9_MOESM1_ESM.pdf]

Appendix Table of Contents

Appendix Figure S1. Plasma Vcan levels are slightly increased in 38-week-old MFS mice.

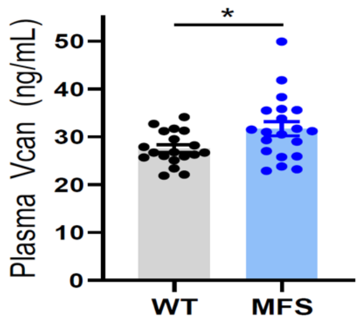

Appendix Figure S1. Plasma Vcan levels are slightly increased in 38-week-old MFS mice.

Plasma Vcan levels in 38-week-old WT (n=19) and MFS mice (n=20).

Data information: Data are shown as mean ± s.e.m. Each data point denotes an individual mouse.

*$p < 0.05$, (unpaired t-test with Welch's correction).
