# Supplementary figures and images for: Versican accumulation drives Nos2 induction and aortic disease in Marfan syndrome via Akt activation
Source: EMBO Mol Med. 2024 Jan 2;16(1):9. doi: 10.1038/s44321-023-00009-7 (PMC10897446; doi:10.1038/s44321-023-00009-7)

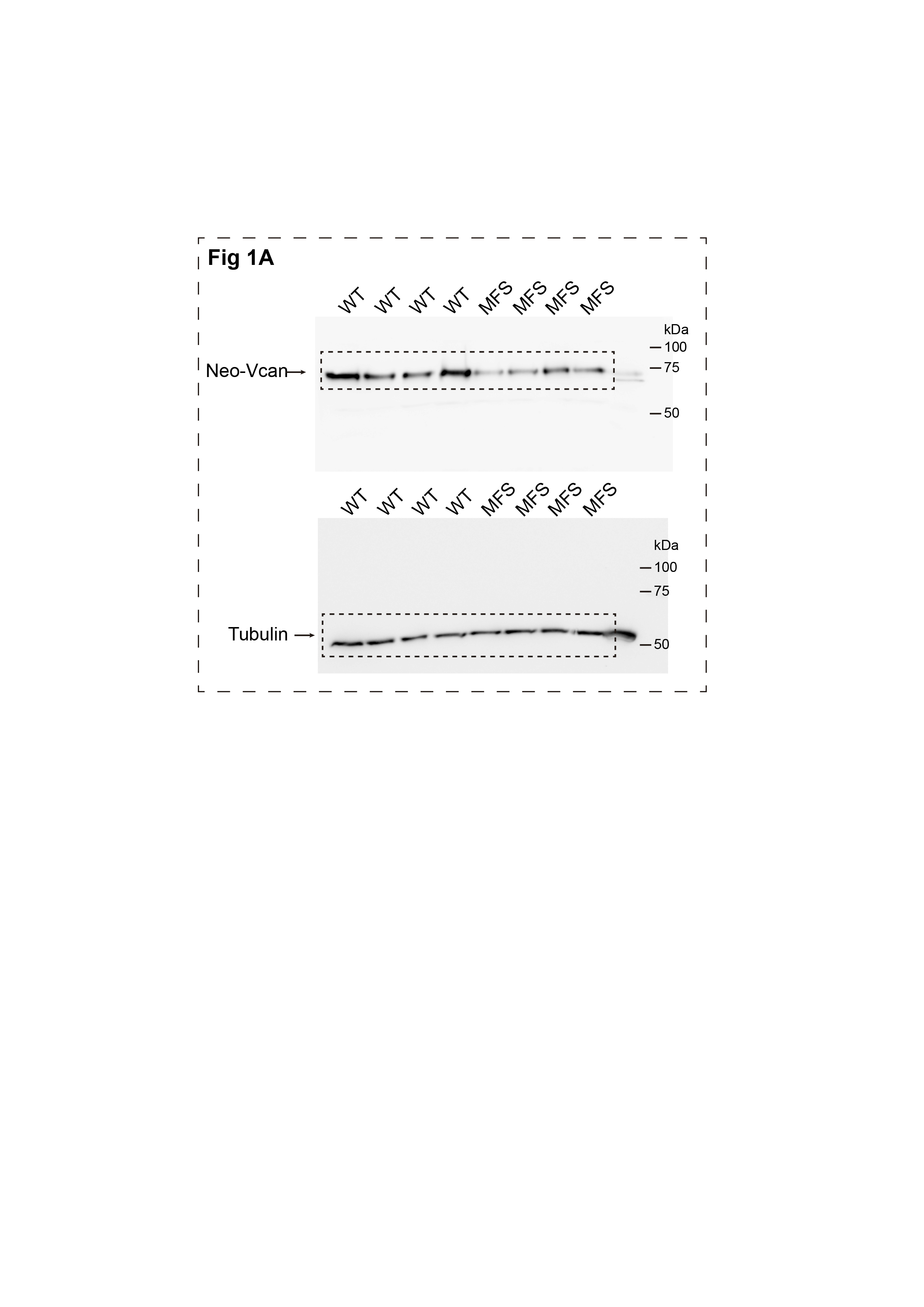

Supplement: Supplementary file 3 — Source Data Fig. 1 [file 44321_2023_9_MOESM3_ESM.zip › Figure 1/1A/cropped-blot.tif]

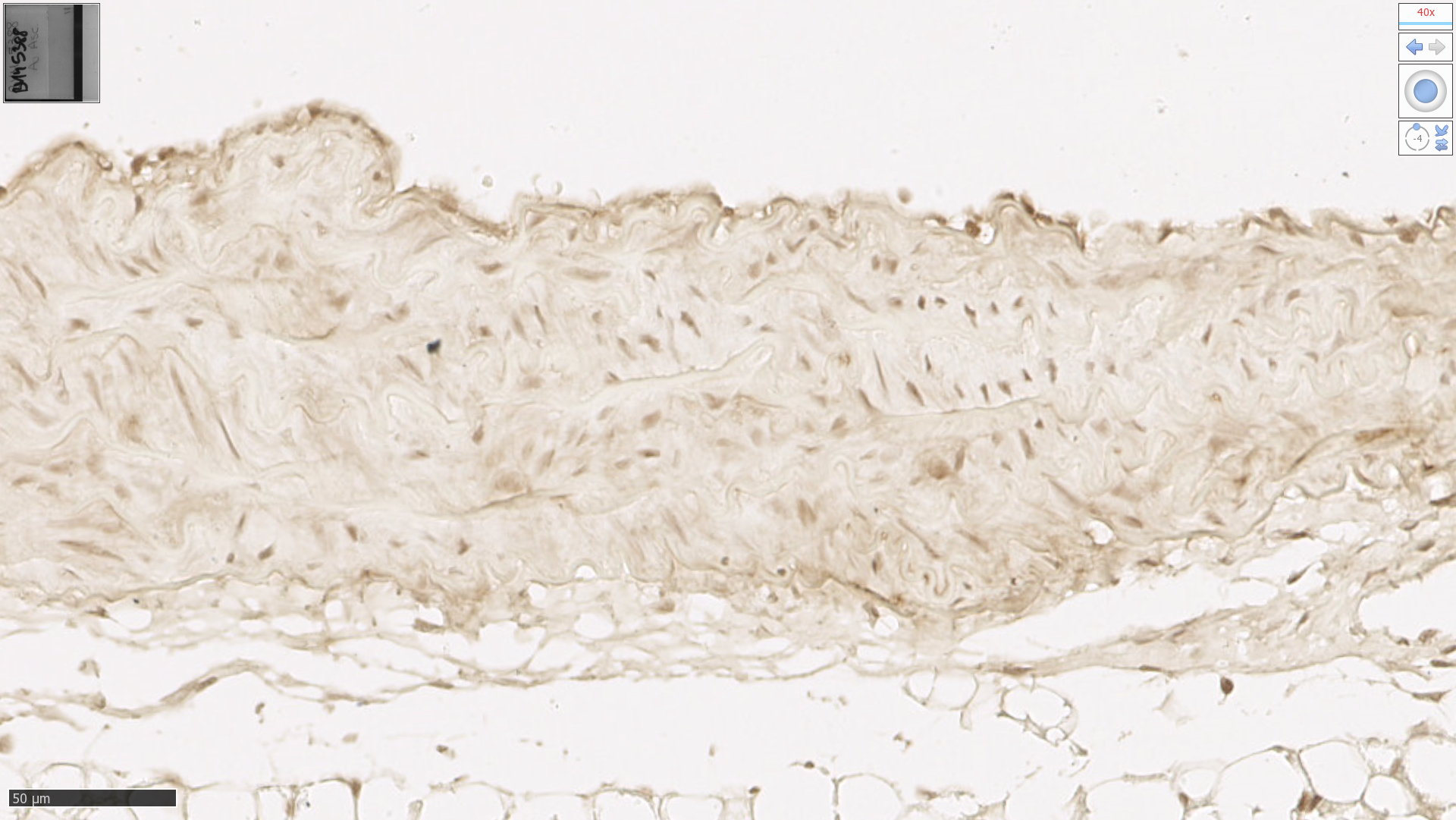

Supplement: Supplementary file 3 — Source Data Fig. 1 [file 44321_2023_9_MOESM3_ESM.zip › Figure 1/1B/neovcan-mfs.tif]

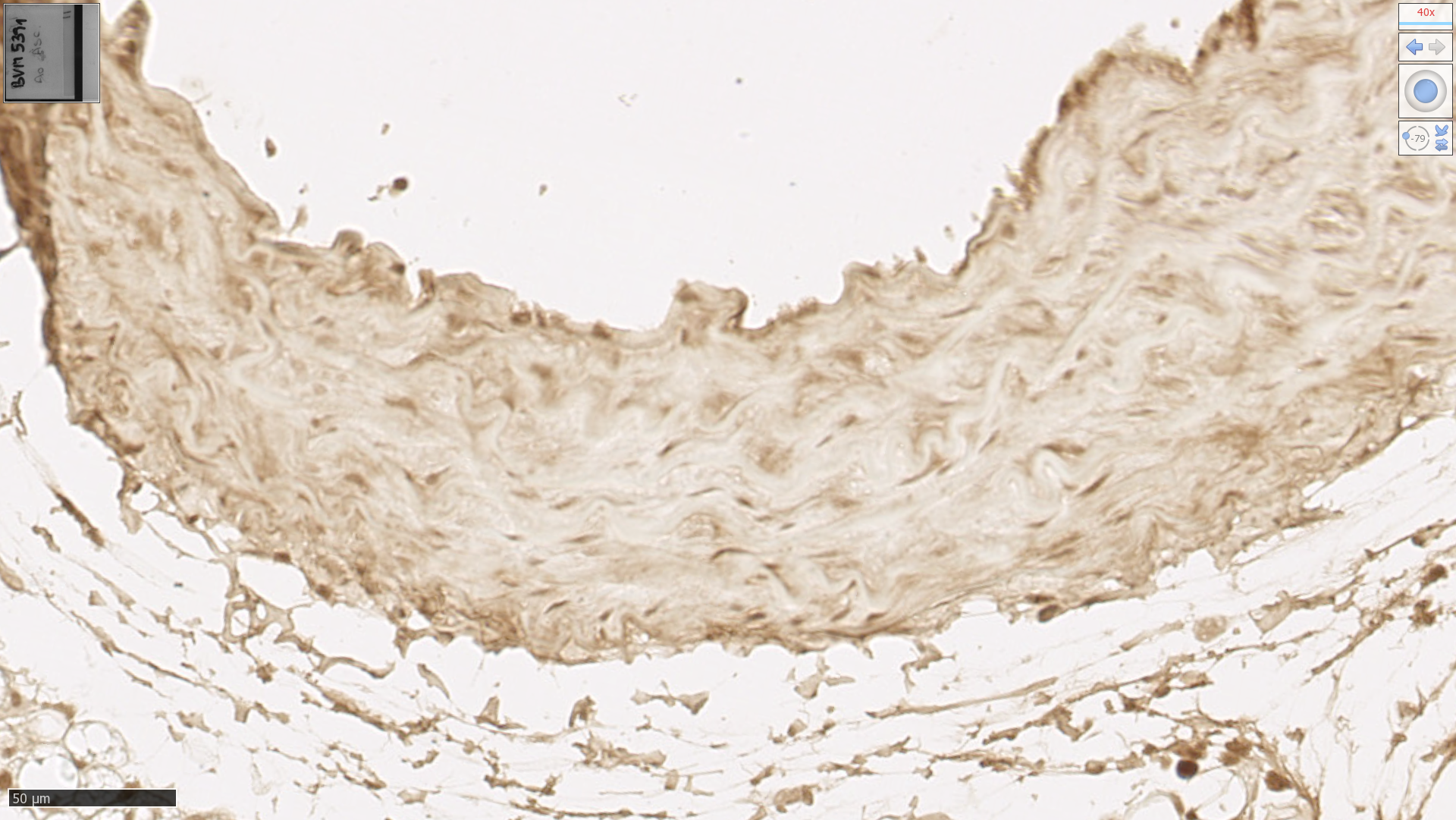

Supplement: Supplementary file 3 — Source Data Fig. 1 [file 44321_2023_9_MOESM3_ESM.zip › Figure 1/1B/neovcan-wt.tif]

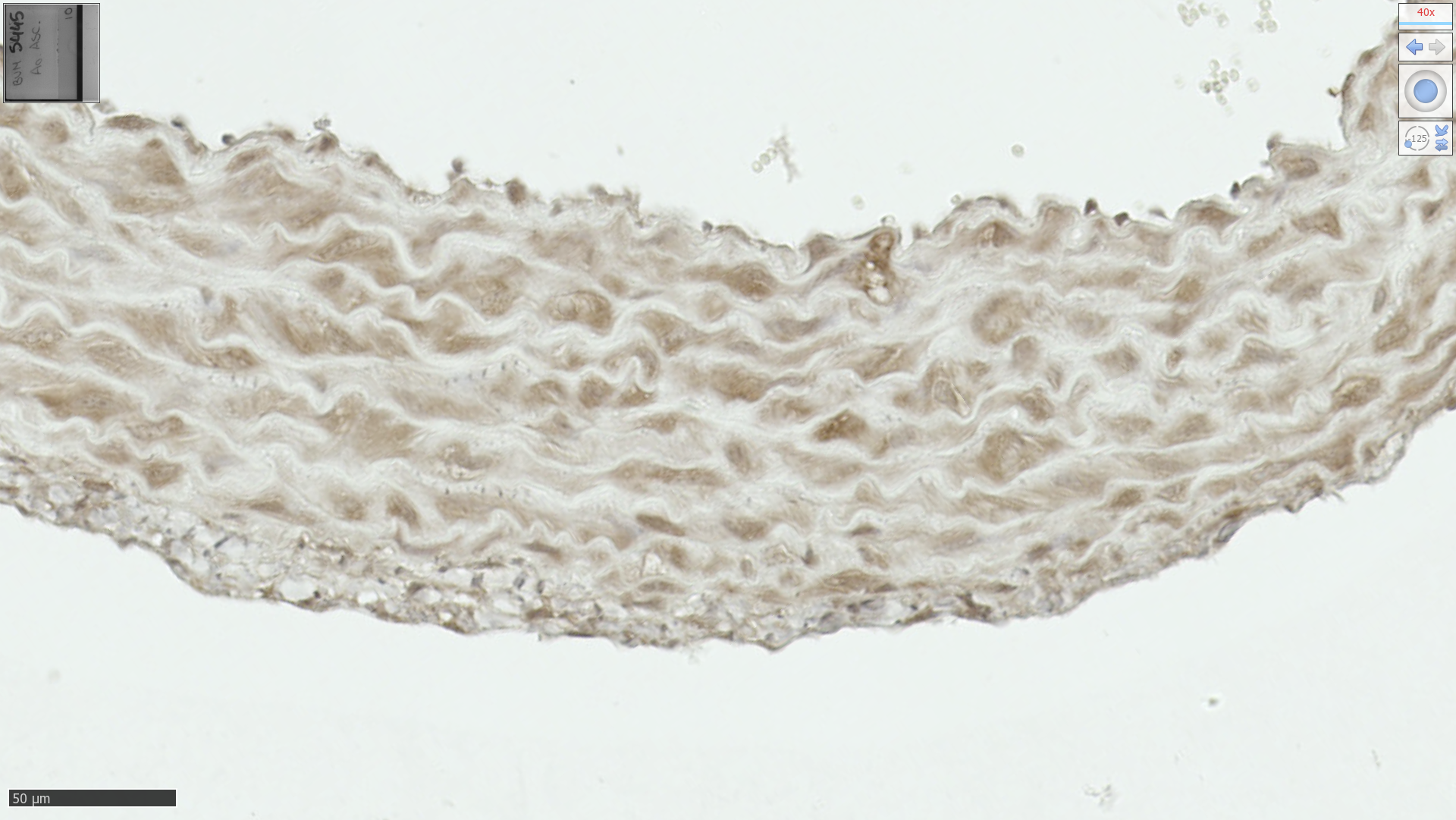

Supplement: Supplementary file 3 — Source Data Fig. 1 [file 44321_2023_9_MOESM3_ESM.zip › Figure 1/1B/vcan-mfs.tif]

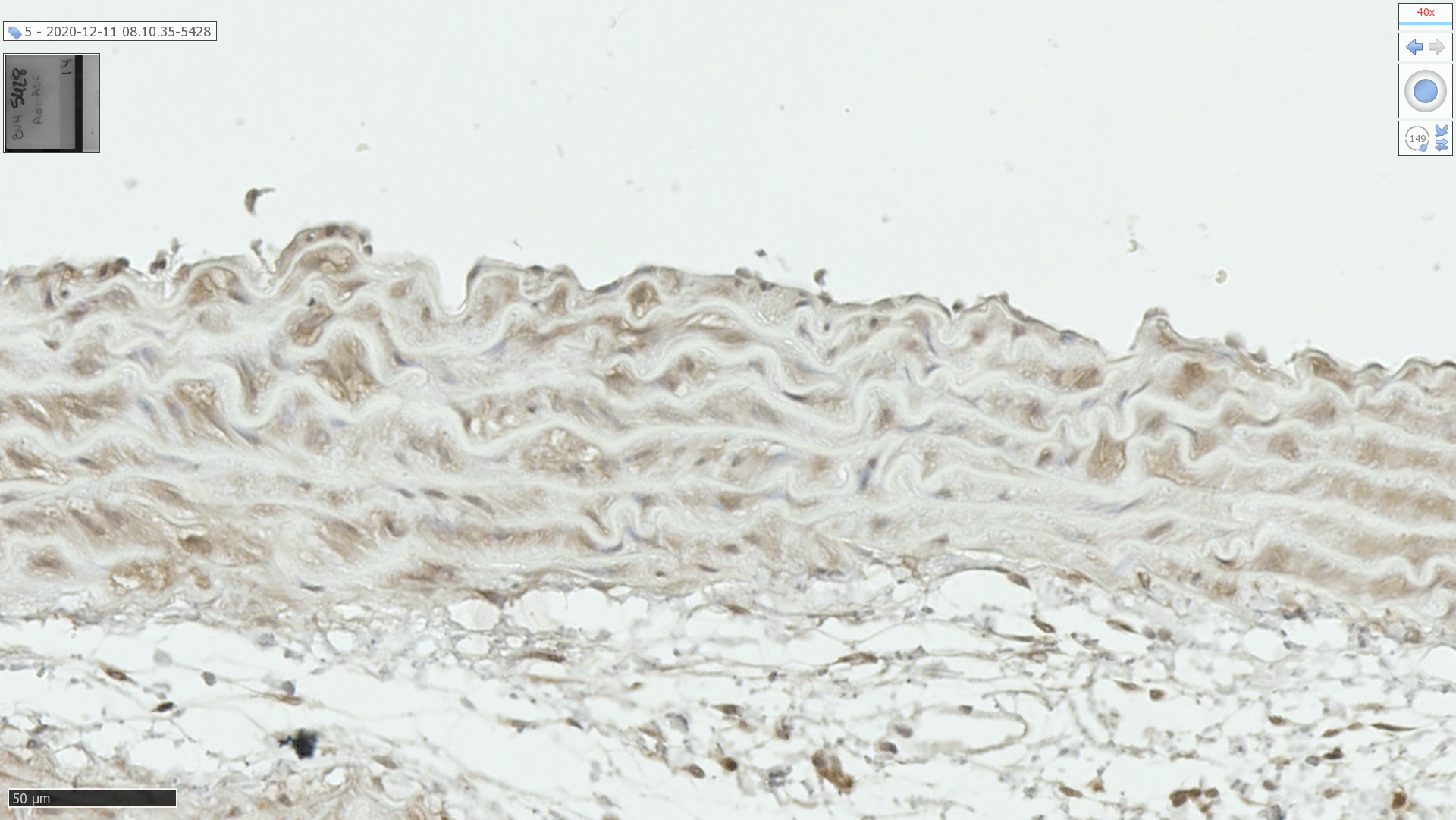

Supplement: Supplementary file 3 — Source Data Fig. 1 [file 44321_2023_9_MOESM3_ESM.zip › Figure 1/1B/vcan-wt.tif]

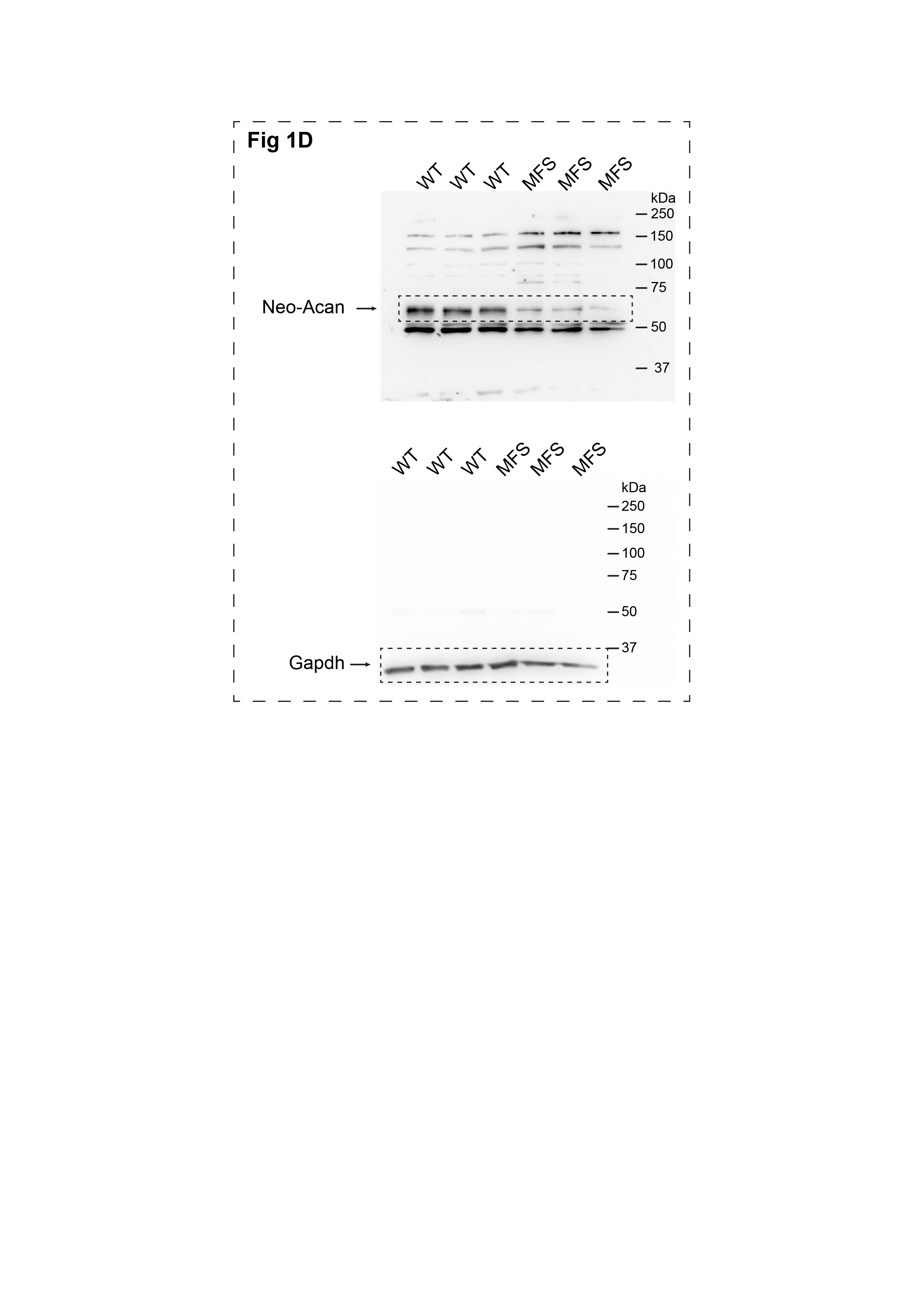

Supplement: Supplementary file 3 — Source Data Fig. 1 [file 44321_2023_9_MOESM3_ESM.zip › Figure 1/1D/cropped-blot.tif]

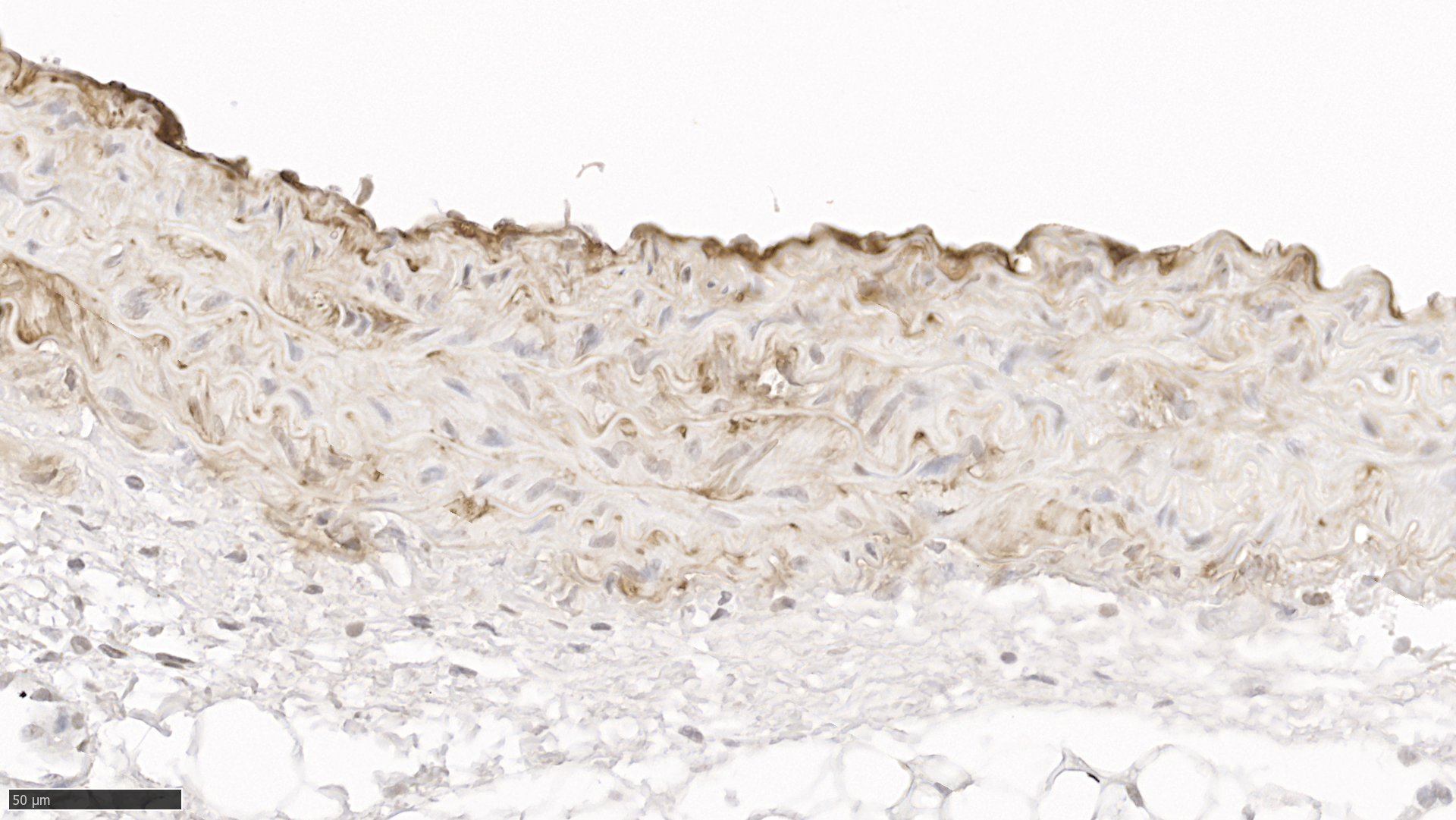

Supplement: Supplementary file 3 — Source Data Fig. 1 [file 44321_2023_9_MOESM3_ESM.zip › Figure 1/1E/acan-mfs.tif]

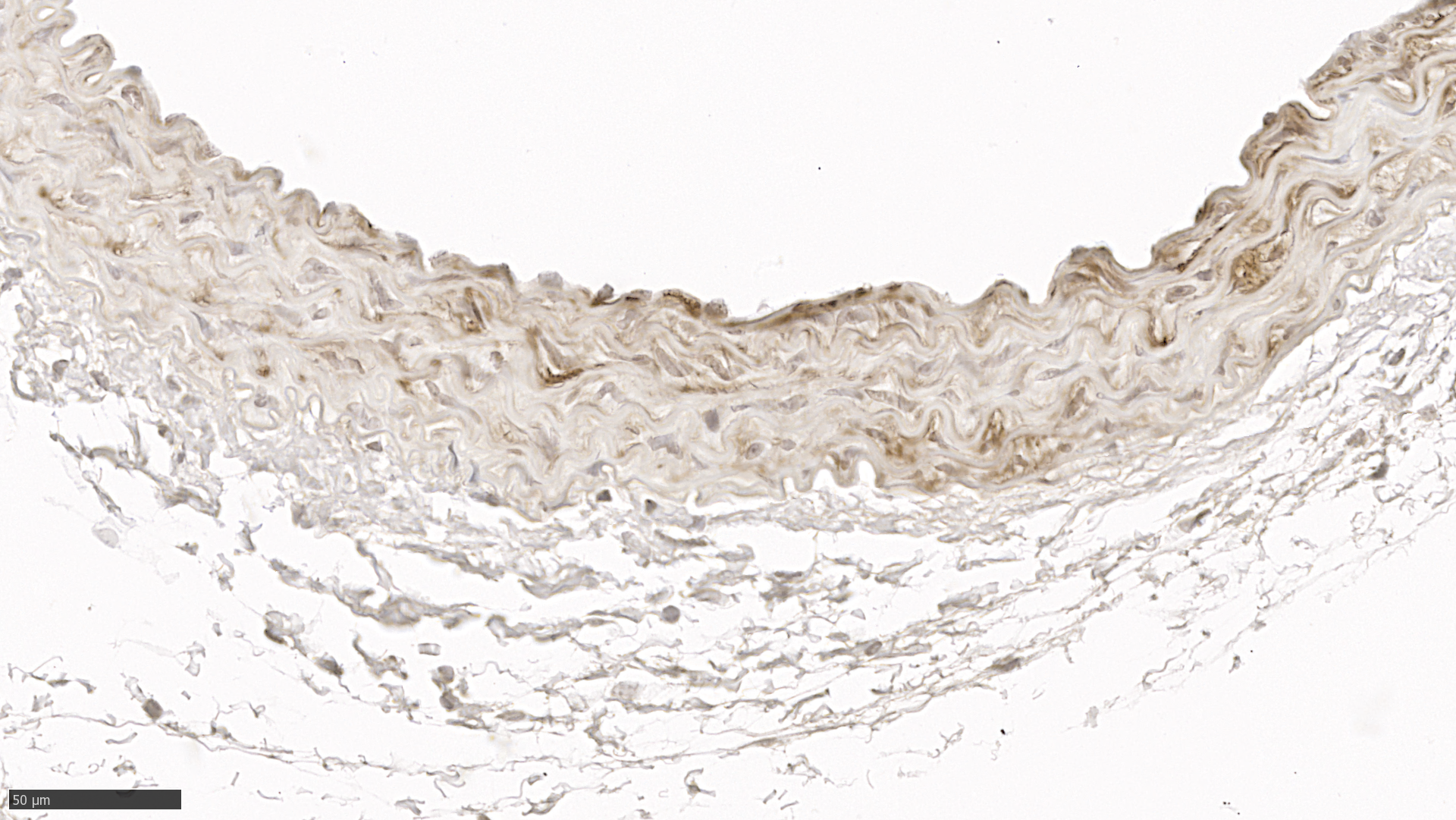

Supplement: Supplementary file 3 — Source Data Fig. 1 [file 44321_2023_9_MOESM3_ESM.zip › Figure 1/1E/acan-wt.tif]

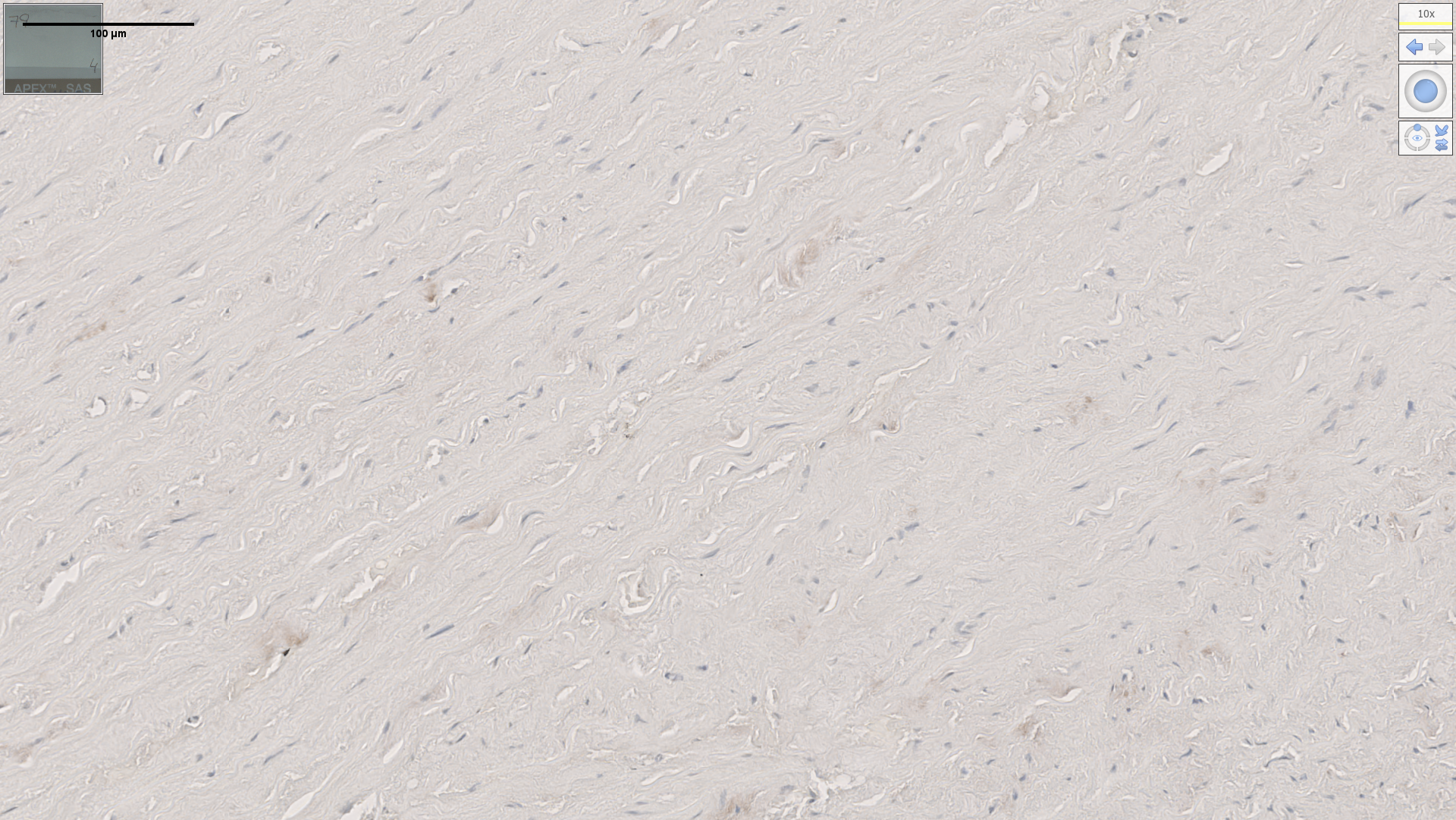

Supplement: Supplementary file 3 — Source Data Fig. 1 [file 44321_2023_9_MOESM3_ESM.zip › Figure 1/1G/ACAN-Control.tif]

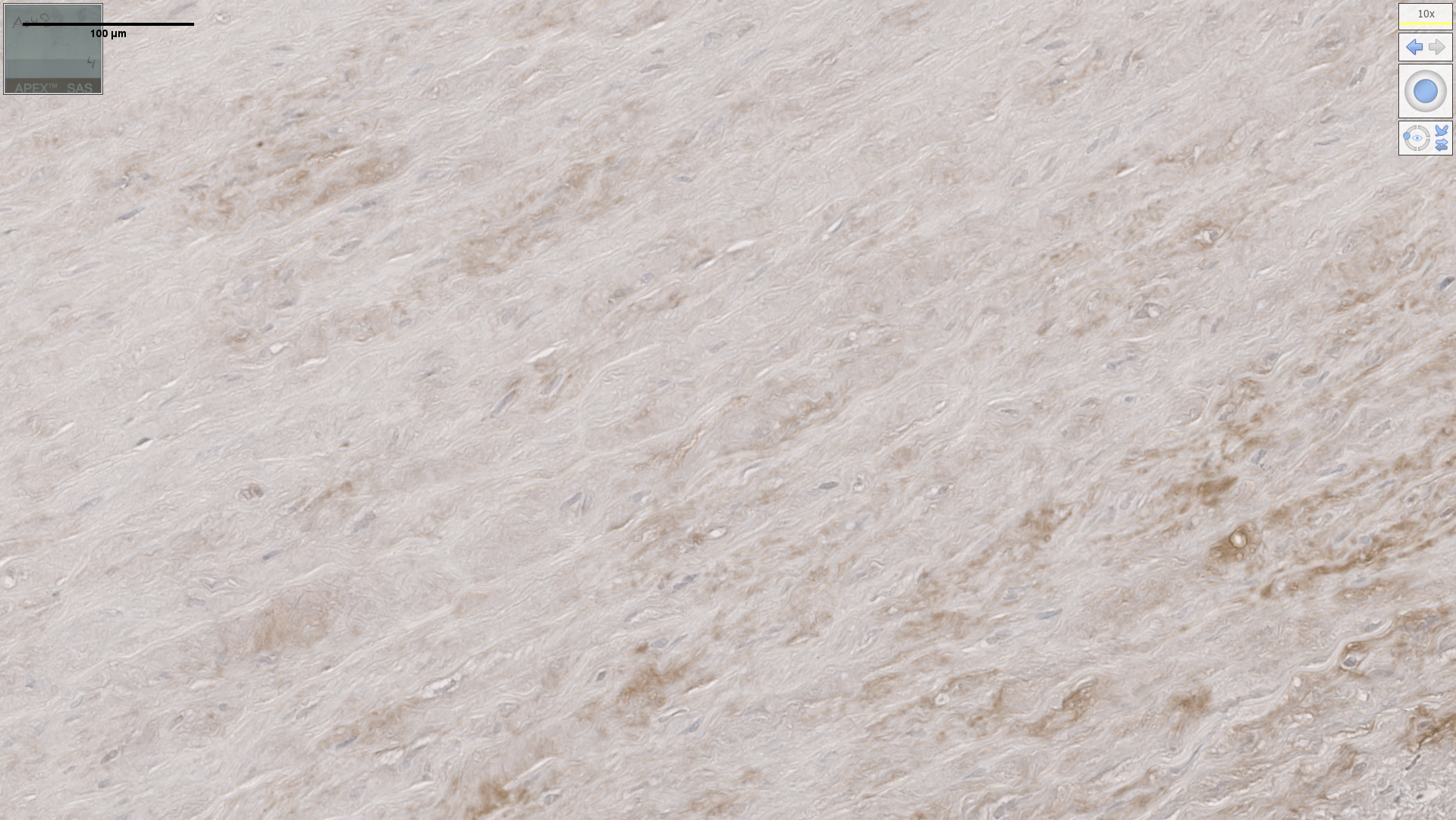

Supplement: Supplementary file 3 — Source Data Fig. 1 [file 44321_2023_9_MOESM3_ESM.zip › Figure 1/1G/ACAN-MFS.tif]

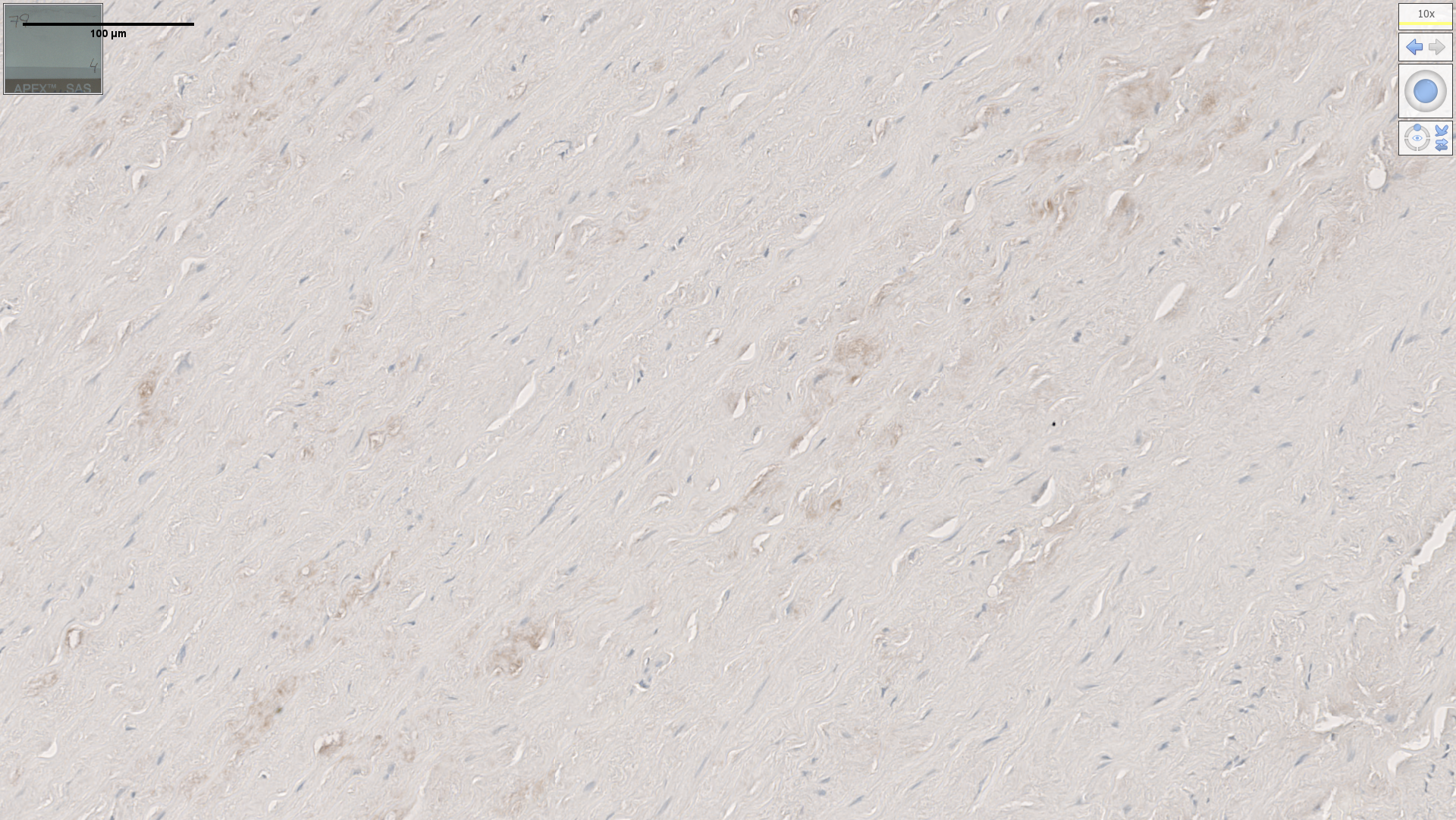

Supplement: Supplementary file 3 — Source Data Fig. 1 [file 44321_2023_9_MOESM3_ESM.zip › Figure 1/1G/VCAN-control.tif]

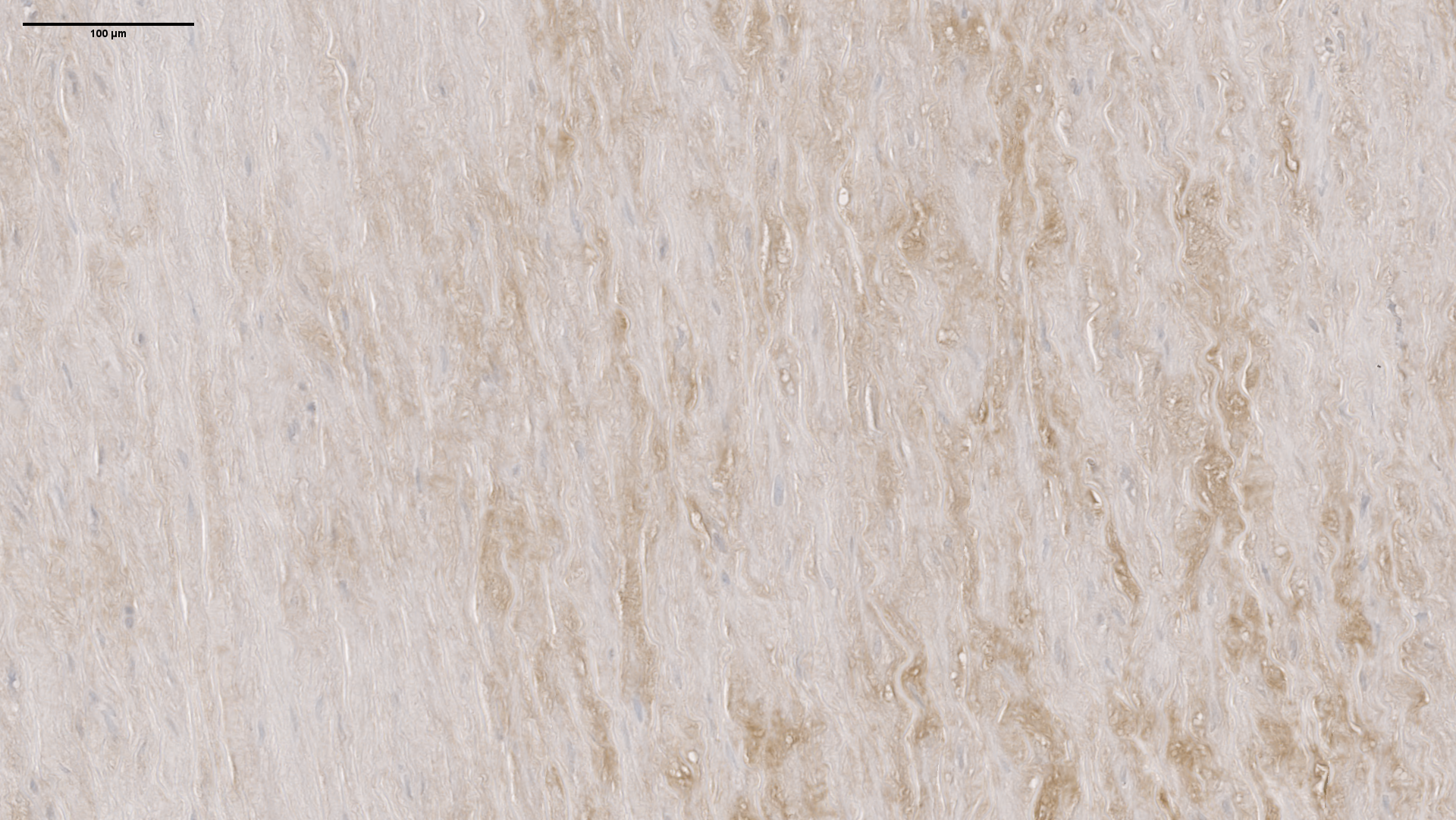

Supplement: Supplementary file 3 — Source Data Fig. 1 [file 44321_2023_9_MOESM3_ESM.zip › Figure 1/1G/VCAN-MFS.tif]

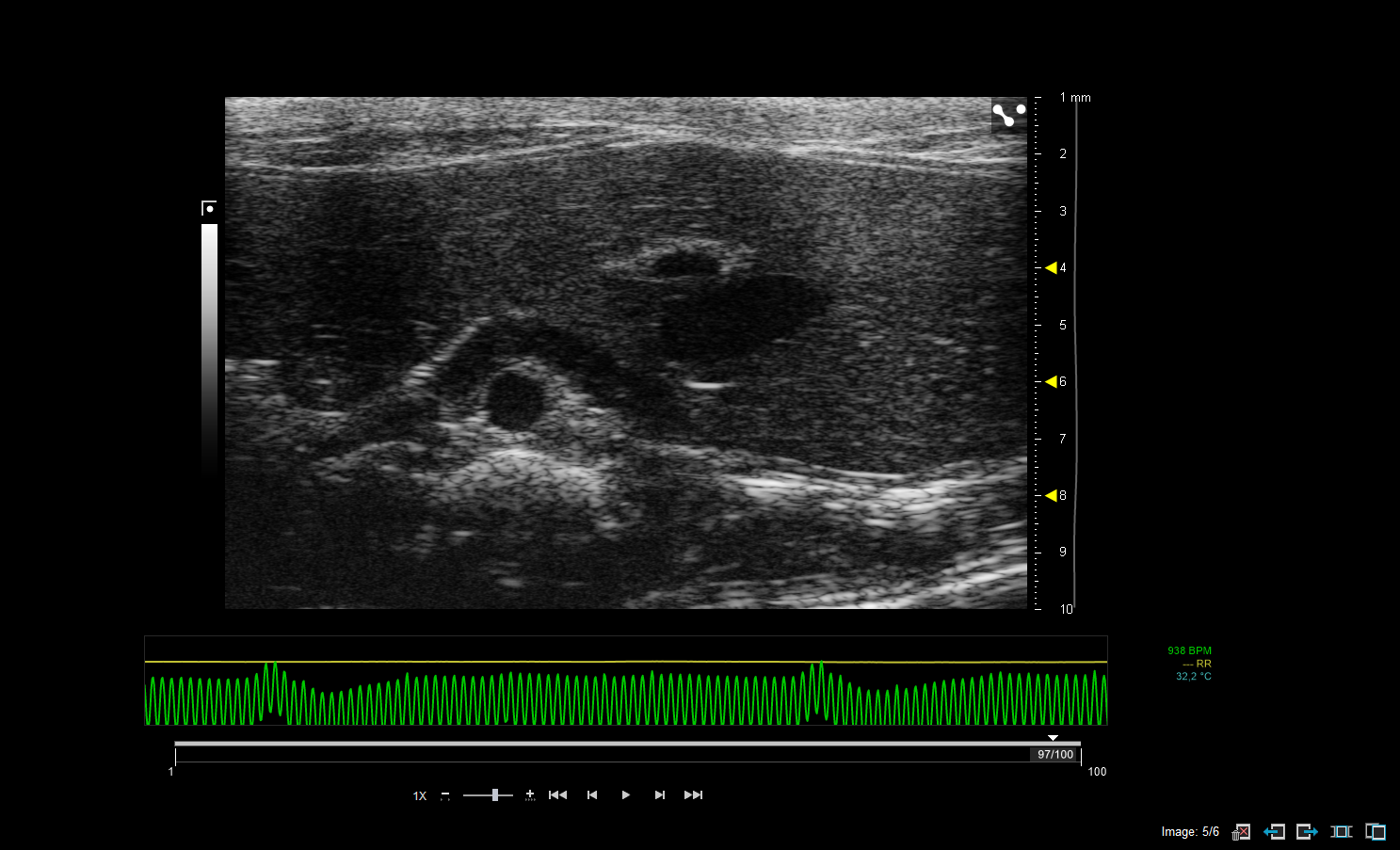

Supplement: Supplementary file 4 — Source Data Fig. 2 [file 44321_2023_9_MOESM4_ESM.zip › Figure 2/2A/abao-hdf.tif]

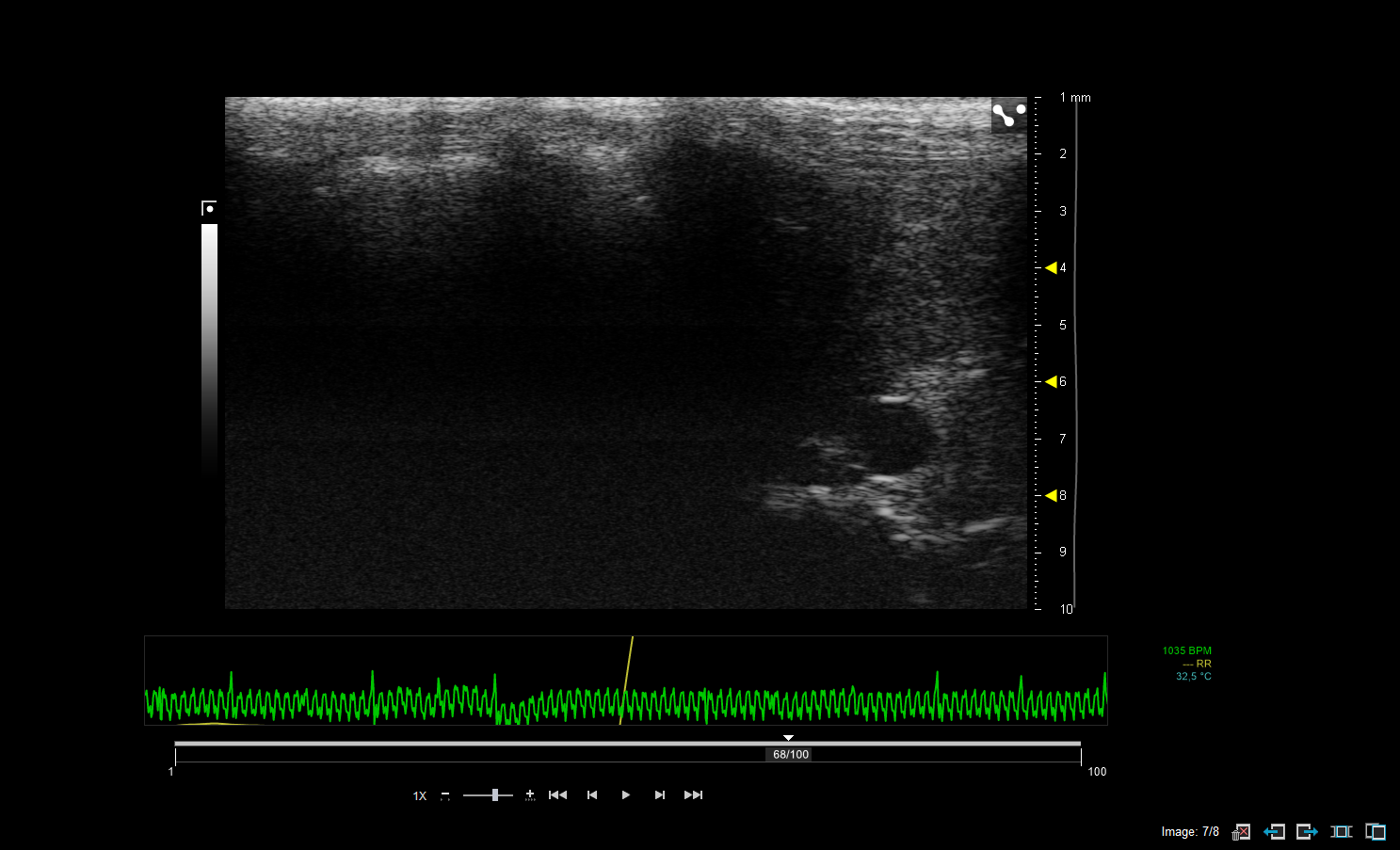

Supplement: Supplementary file 4 — Source Data Fig. 2 [file 44321_2023_9_MOESM4_ESM.zip › Figure 2/2A/abao-mfs.tif]

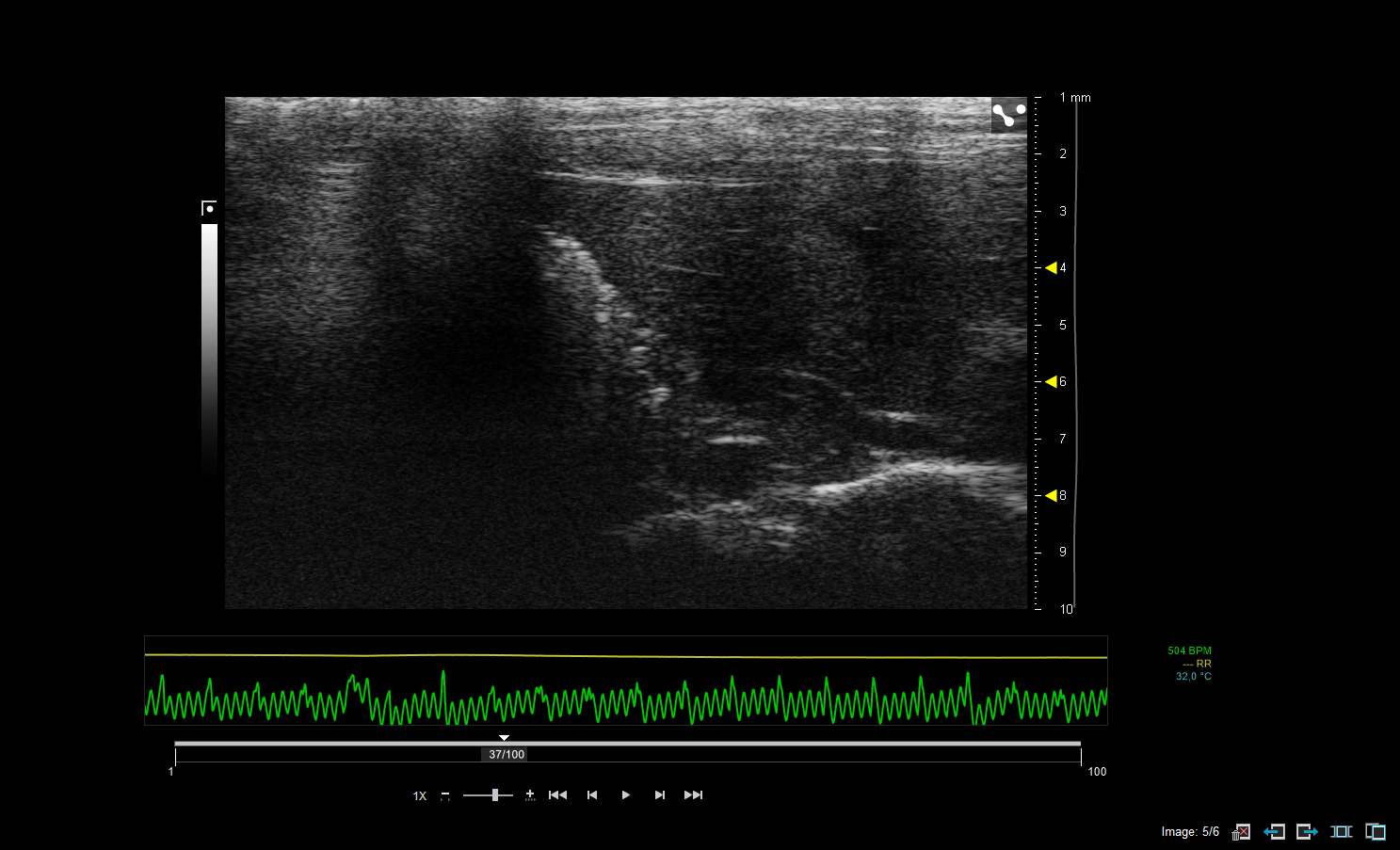

Supplement: Supplementary file 4 — Source Data Fig. 2 [file 44321_2023_9_MOESM4_ESM.zip › Figure 2/2A/abao-mfs-hdf.tif]

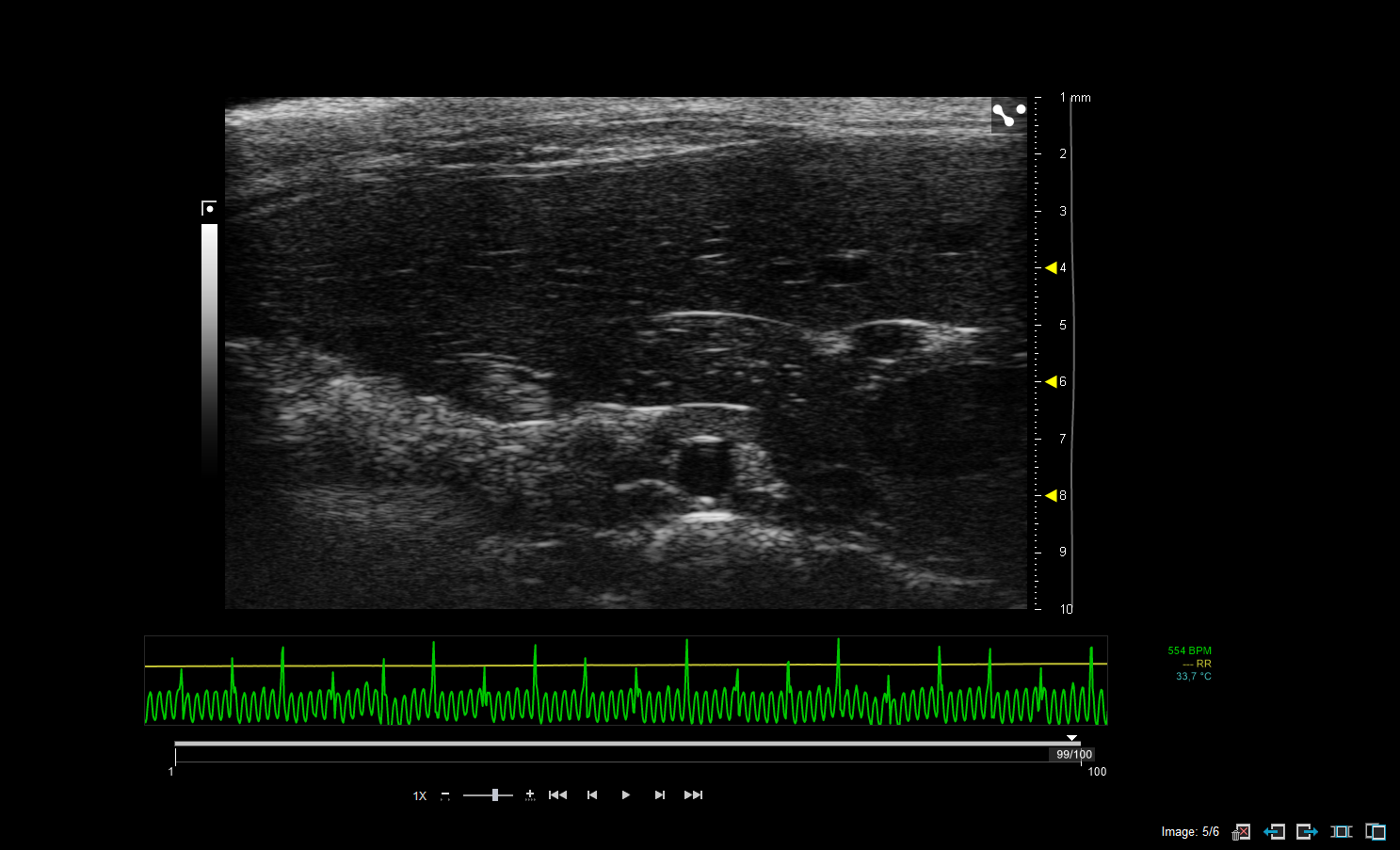

Supplement: Supplementary file 4 — Source Data Fig. 2 [file 44321_2023_9_MOESM4_ESM.zip › Figure 2/2A/abao-wt.tif]

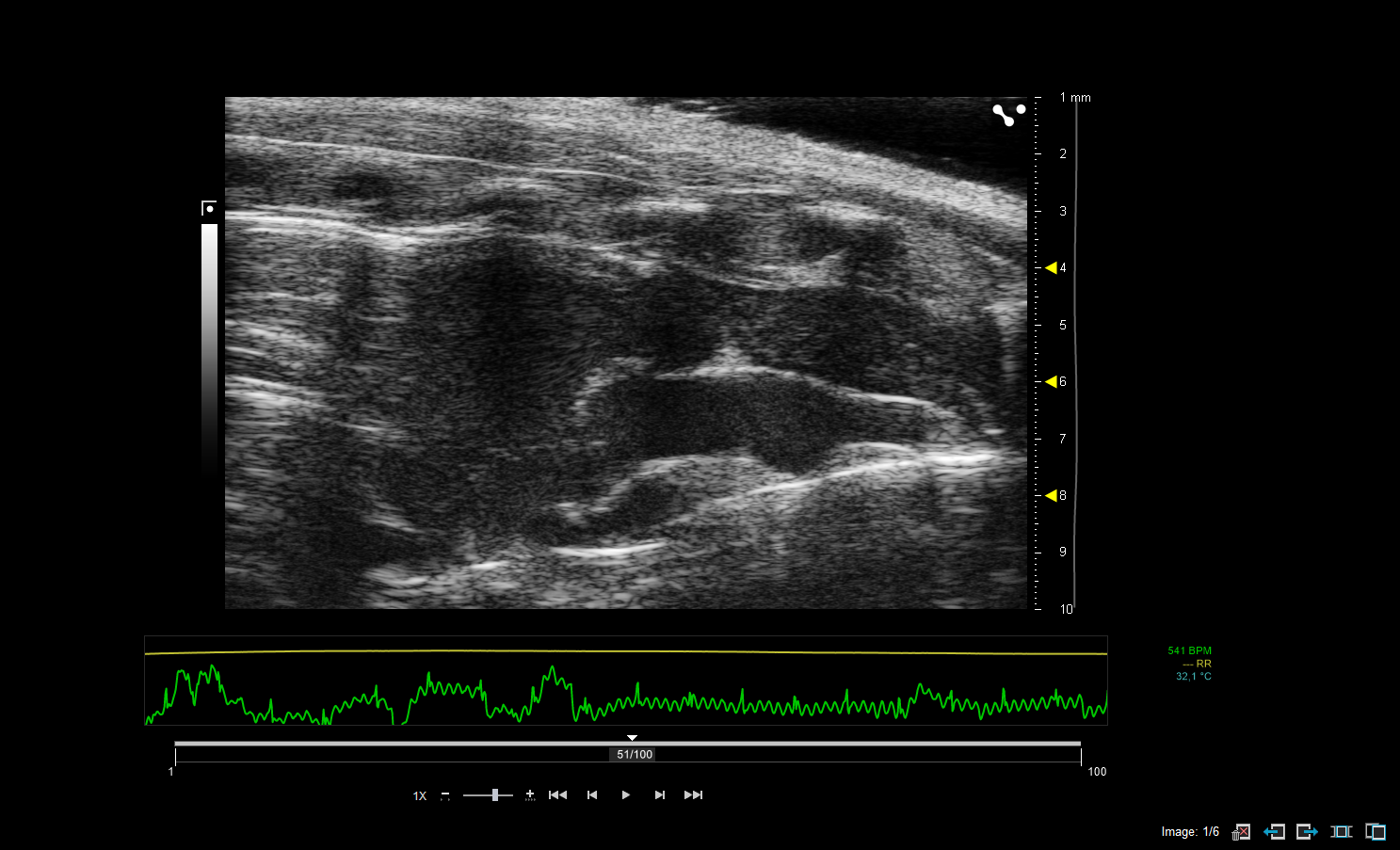

Supplement: Supplementary file 4 — Source Data Fig. 2 [file 44321_2023_9_MOESM4_ESM.zip › Figure 2/2A/asao-hdf.tif]

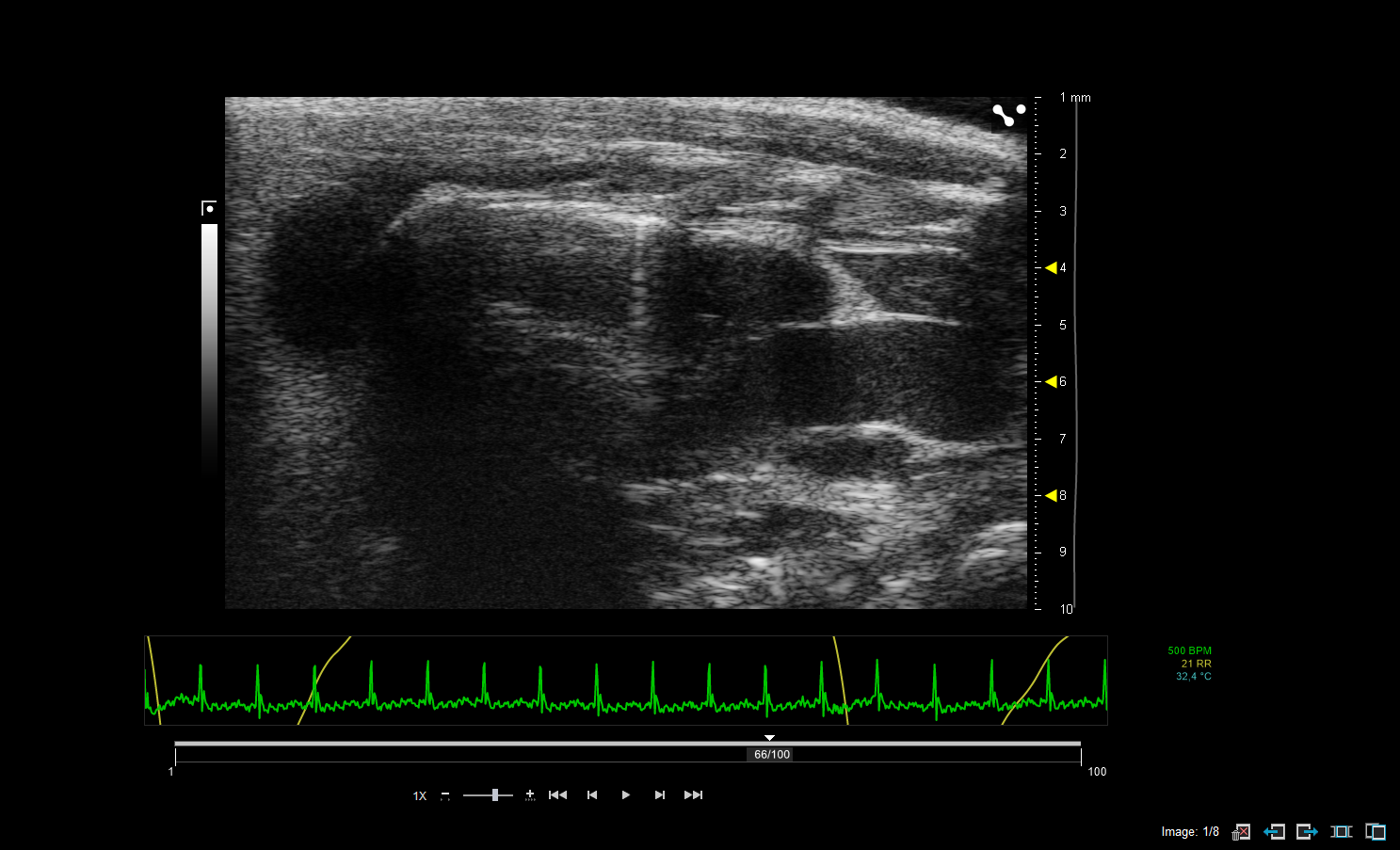

Supplement: Supplementary file 4 — Source Data Fig. 2 [file 44321_2023_9_MOESM4_ESM.zip › Figure 2/2A/asao-mfs.tif]

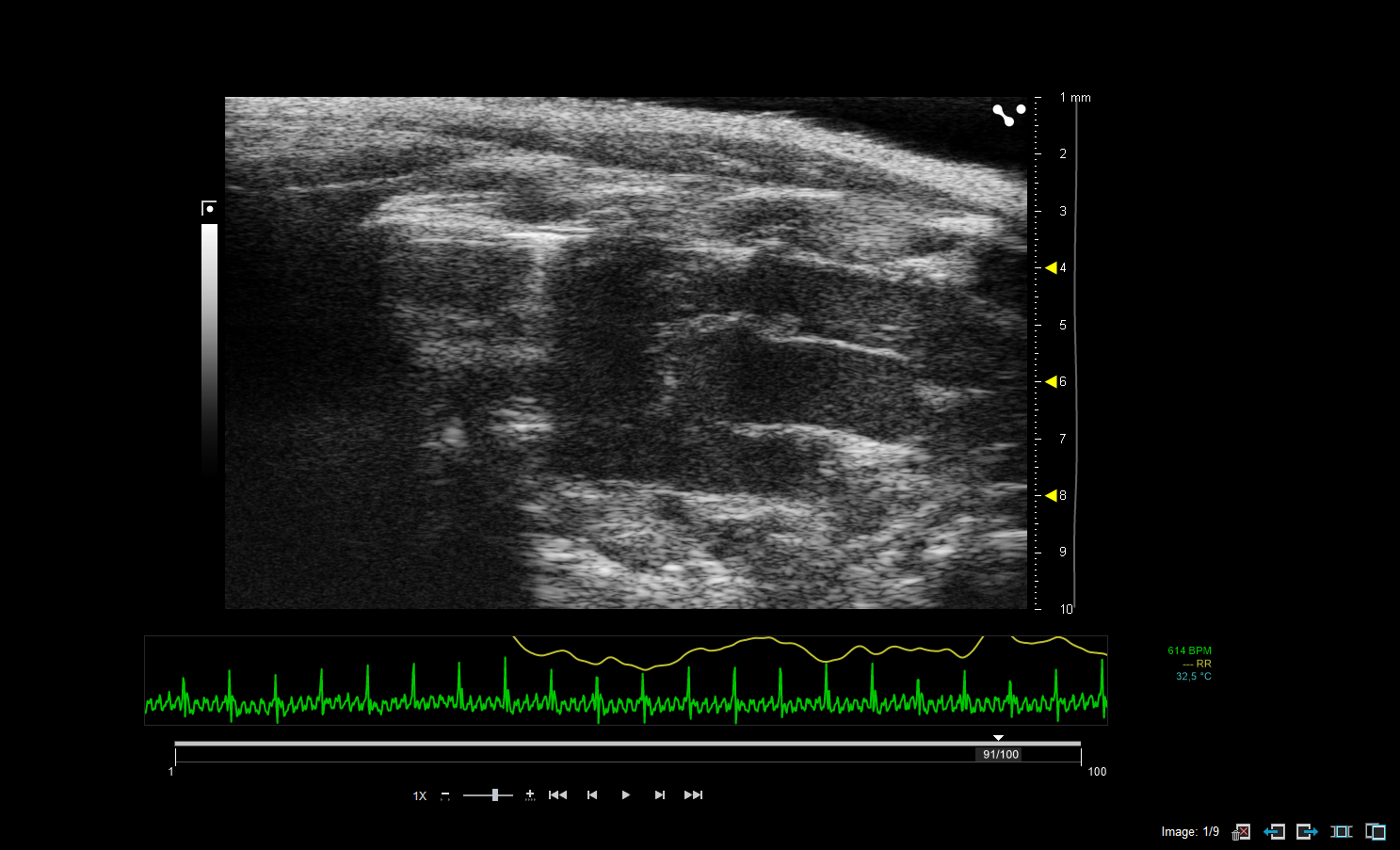

Supplement: Supplementary file 4 — Source Data Fig. 2 [file 44321_2023_9_MOESM4_ESM.zip › Figure 2/2A/asao-mfs-hdf.tif]

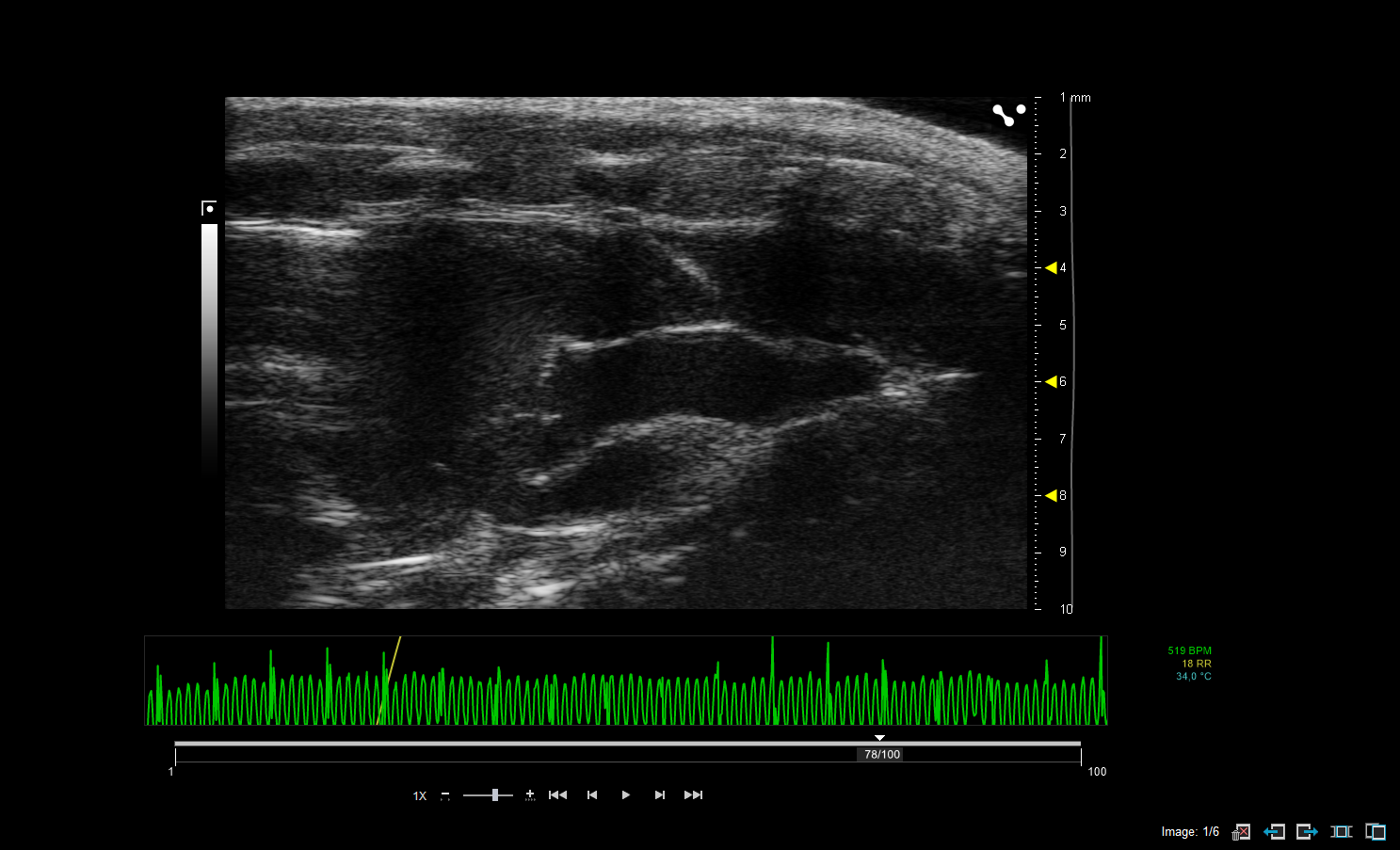

Supplement: Supplementary file 4 — Source Data Fig. 2 [file 44321_2023_9_MOESM4_ESM.zip › Figure 2/2A/asao-wt.tif]

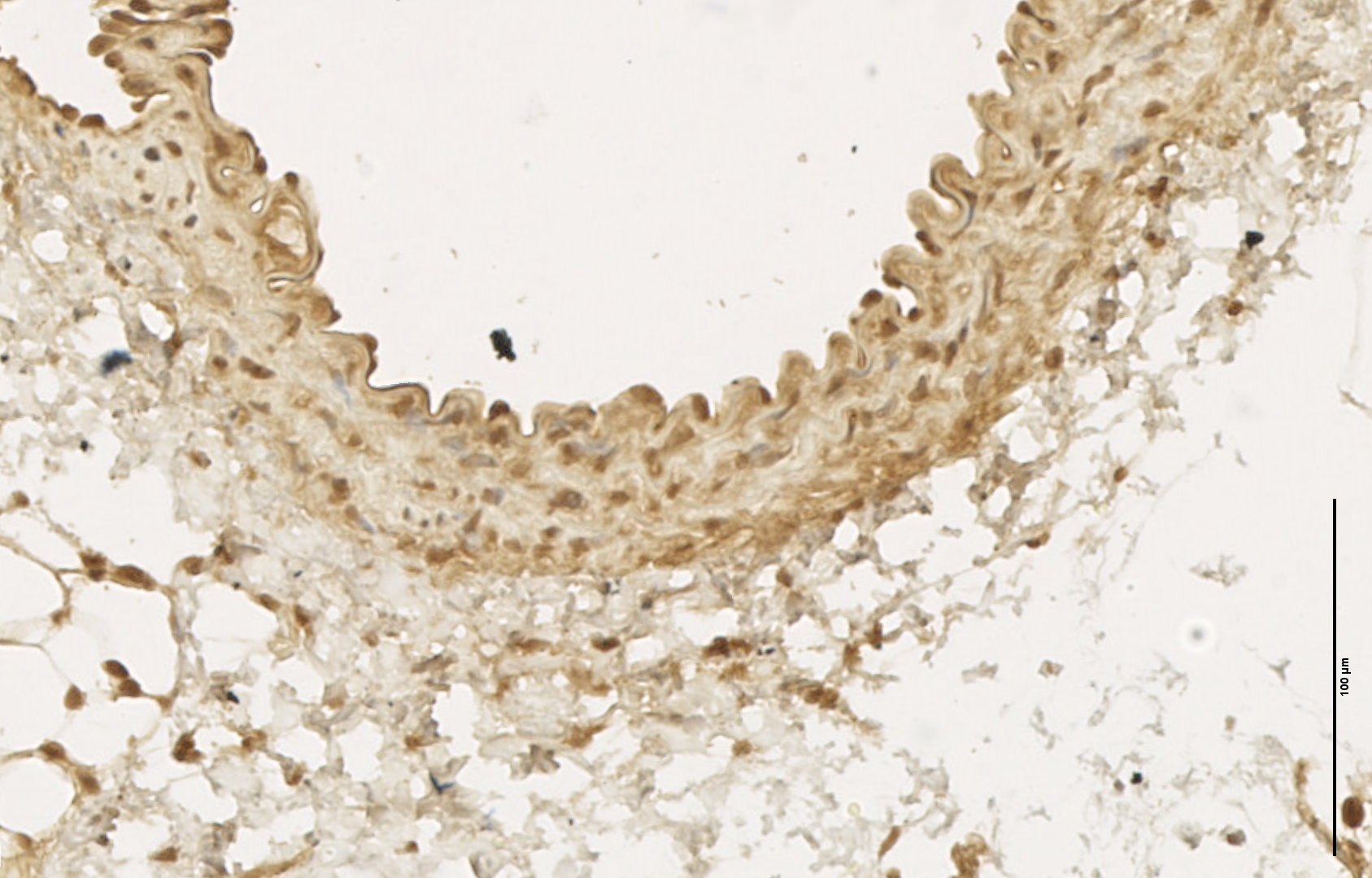

Supplement: Supplementary file 6 — Source Data Fig. 4 [file 44321_2023_9_MOESM6_ESM.zip › Figure 4/4C/ihq-gfp-abao-mfs-acan.tif]

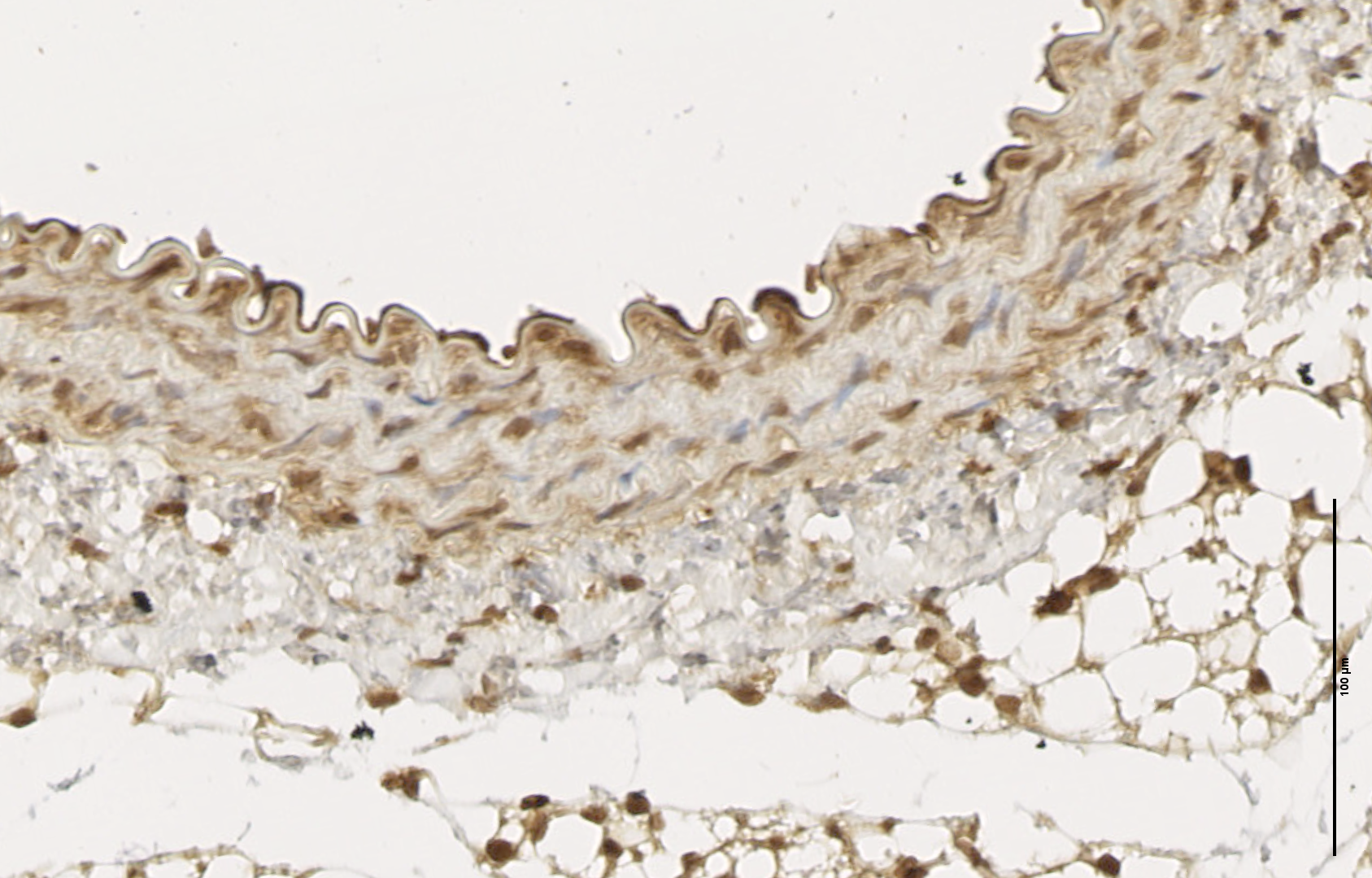

Supplement: Supplementary file 6 — Source Data Fig. 4 [file 44321_2023_9_MOESM6_ESM.zip › Figure 4/4C/ihq-gfp-abao-mfs-scr.tif]

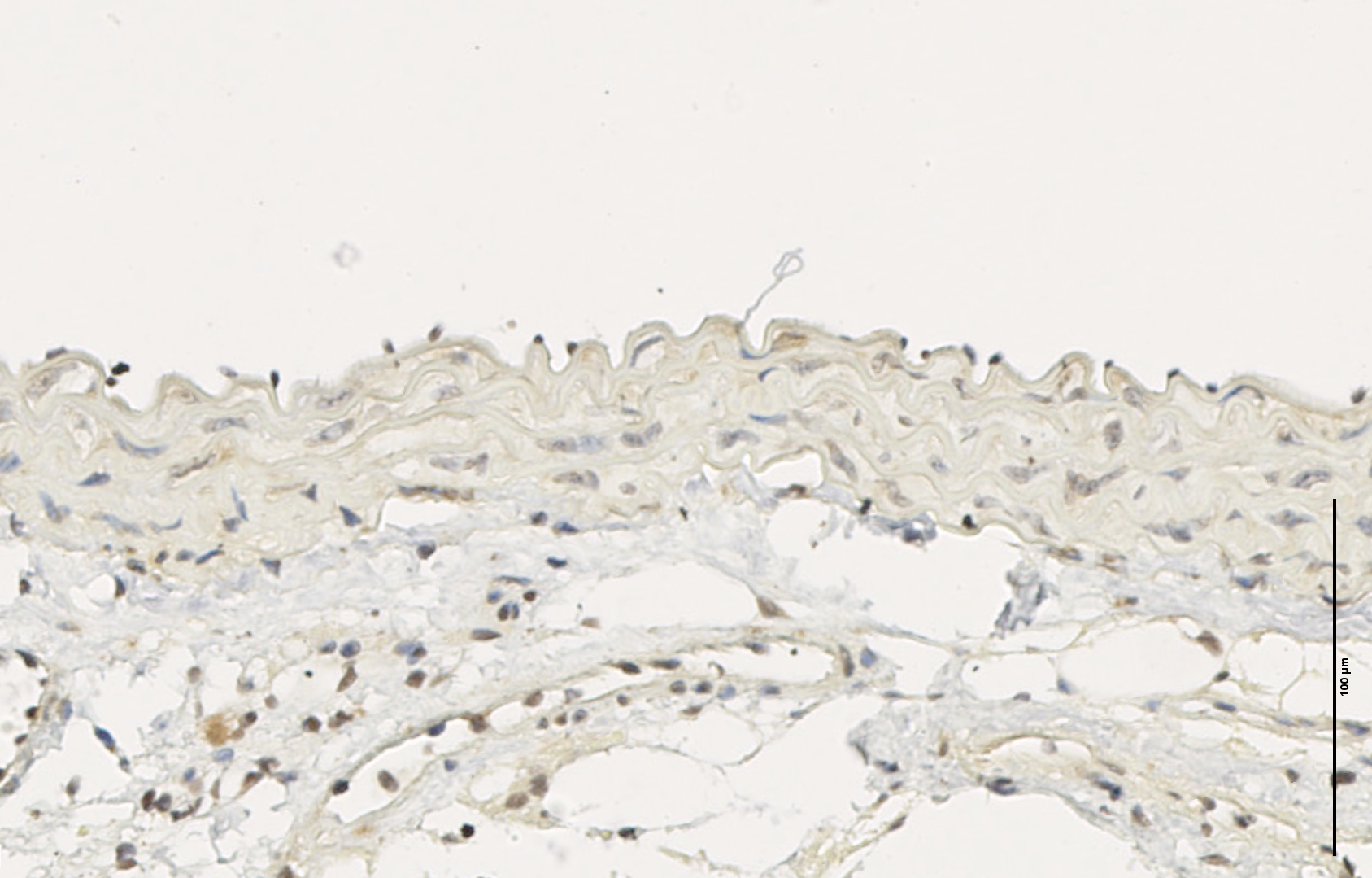

Supplement: Supplementary file 6 — Source Data Fig. 4 [file 44321_2023_9_MOESM6_ESM.zip › Figure 4/4C/ihq-gfp-abao-uninfected.tif]

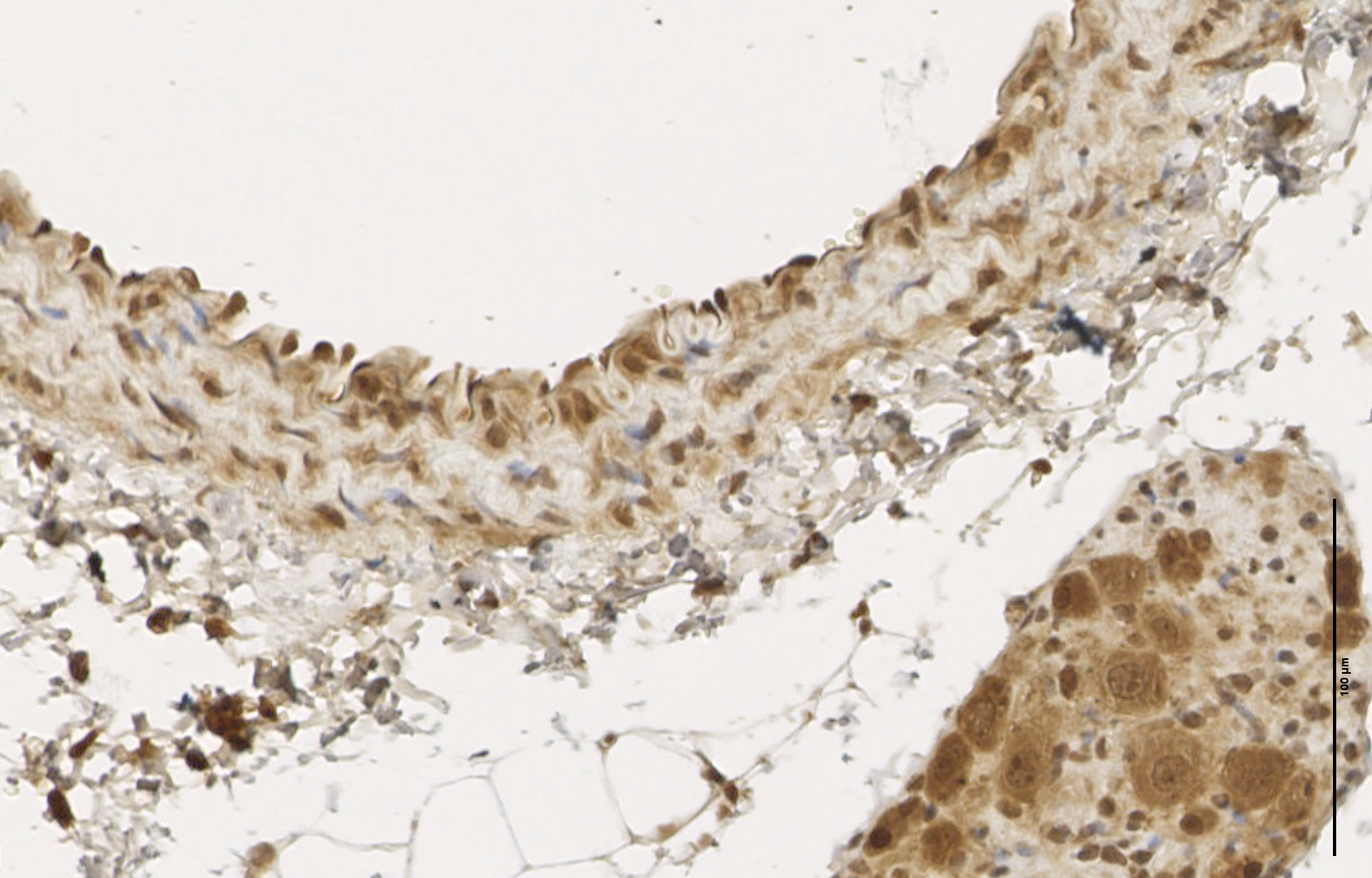

Supplement: Supplementary file 6 — Source Data Fig. 4 [file 44321_2023_9_MOESM6_ESM.zip › Figure 4/4C/ihq-gfp-abao-wt-acan.tif]

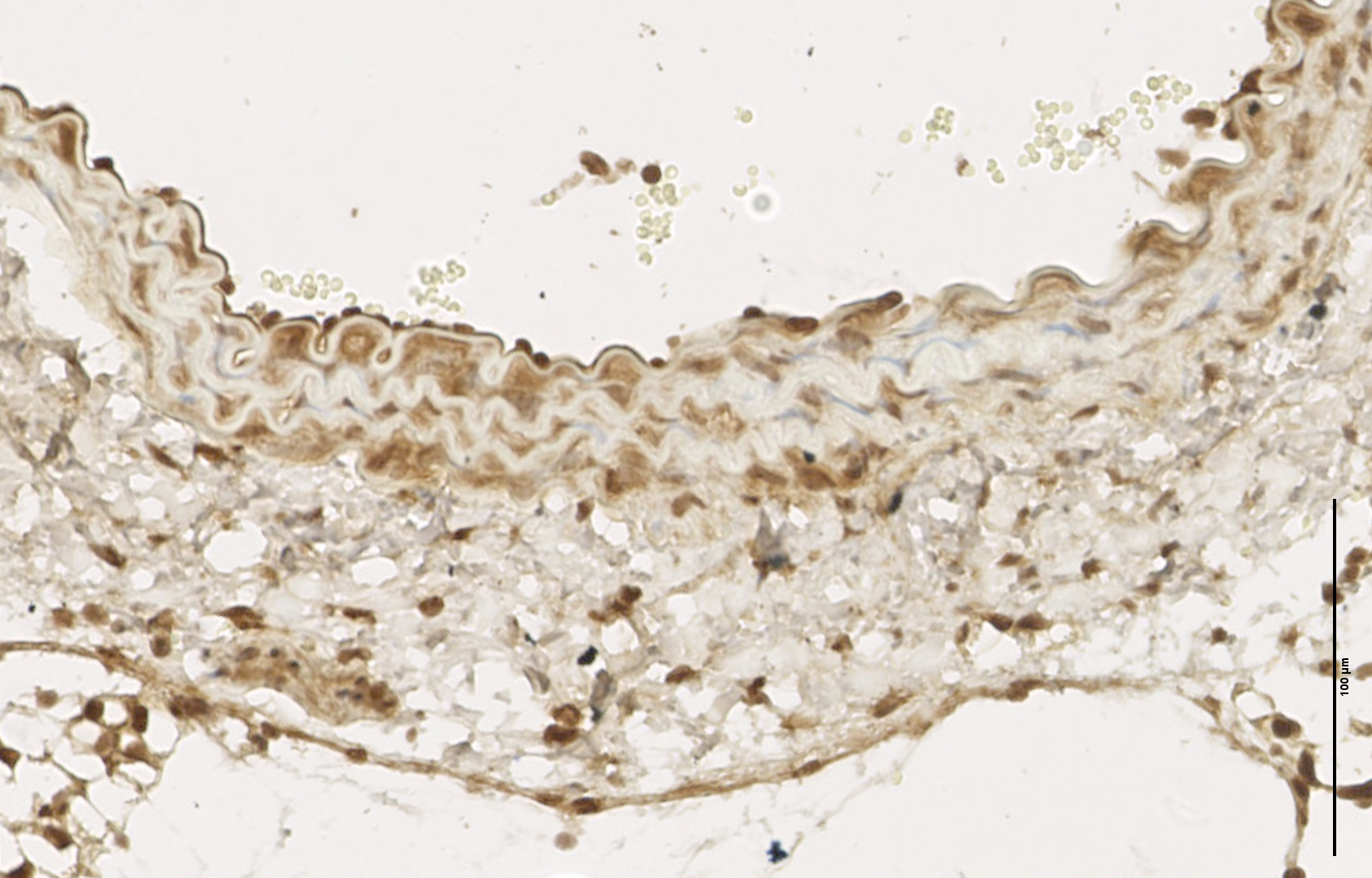

Supplement: Supplementary file 6 — Source Data Fig. 4 [file 44321_2023_9_MOESM6_ESM.zip › Figure 4/4C/ihq-gfp-abao-wt-scr.tif]

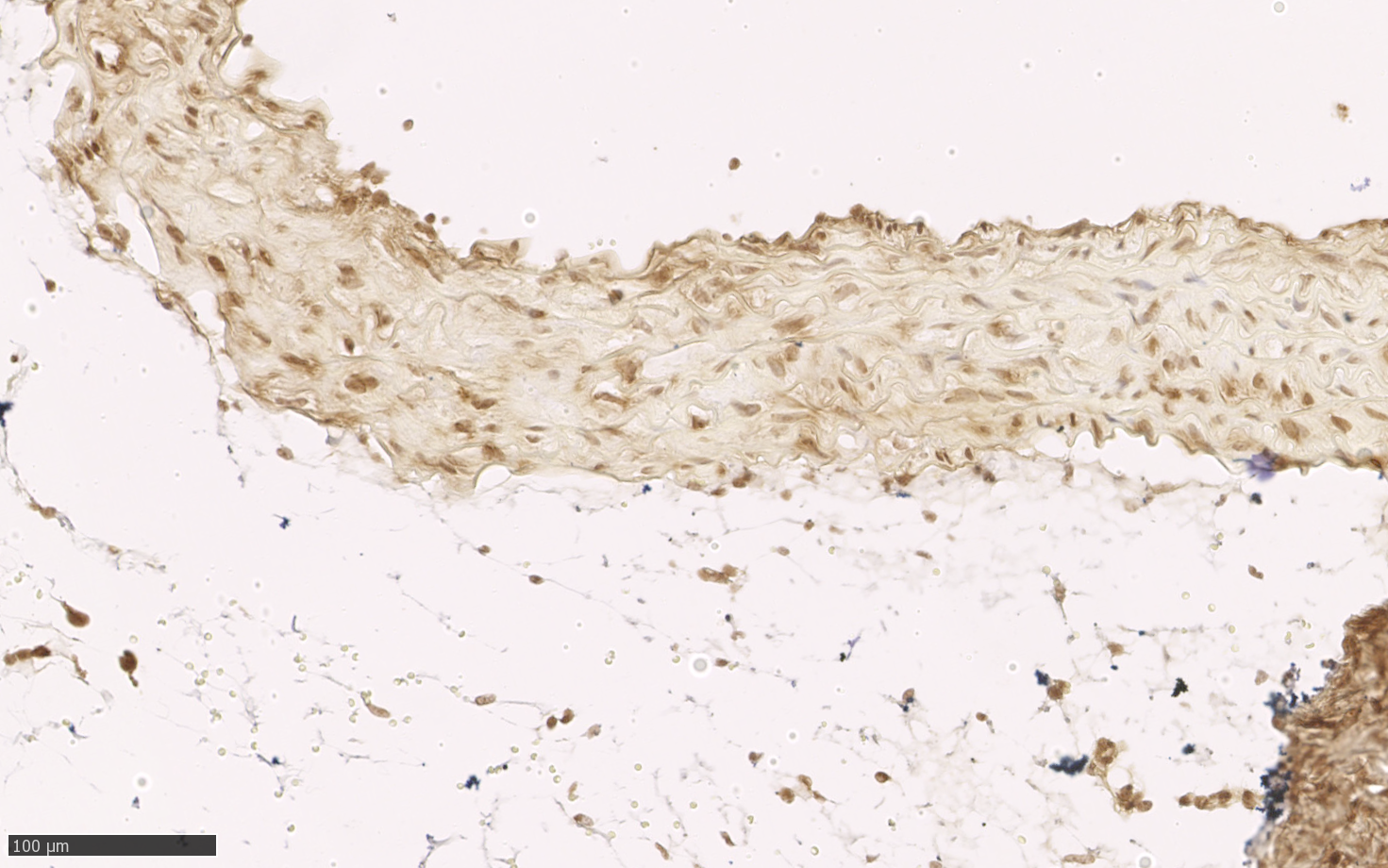

Supplement: Supplementary file 6 — Source Data Fig. 4 [file 44321_2023_9_MOESM6_ESM.zip › Figure 4/4C/ihq-gfp-asao-mfs-acan.tif]

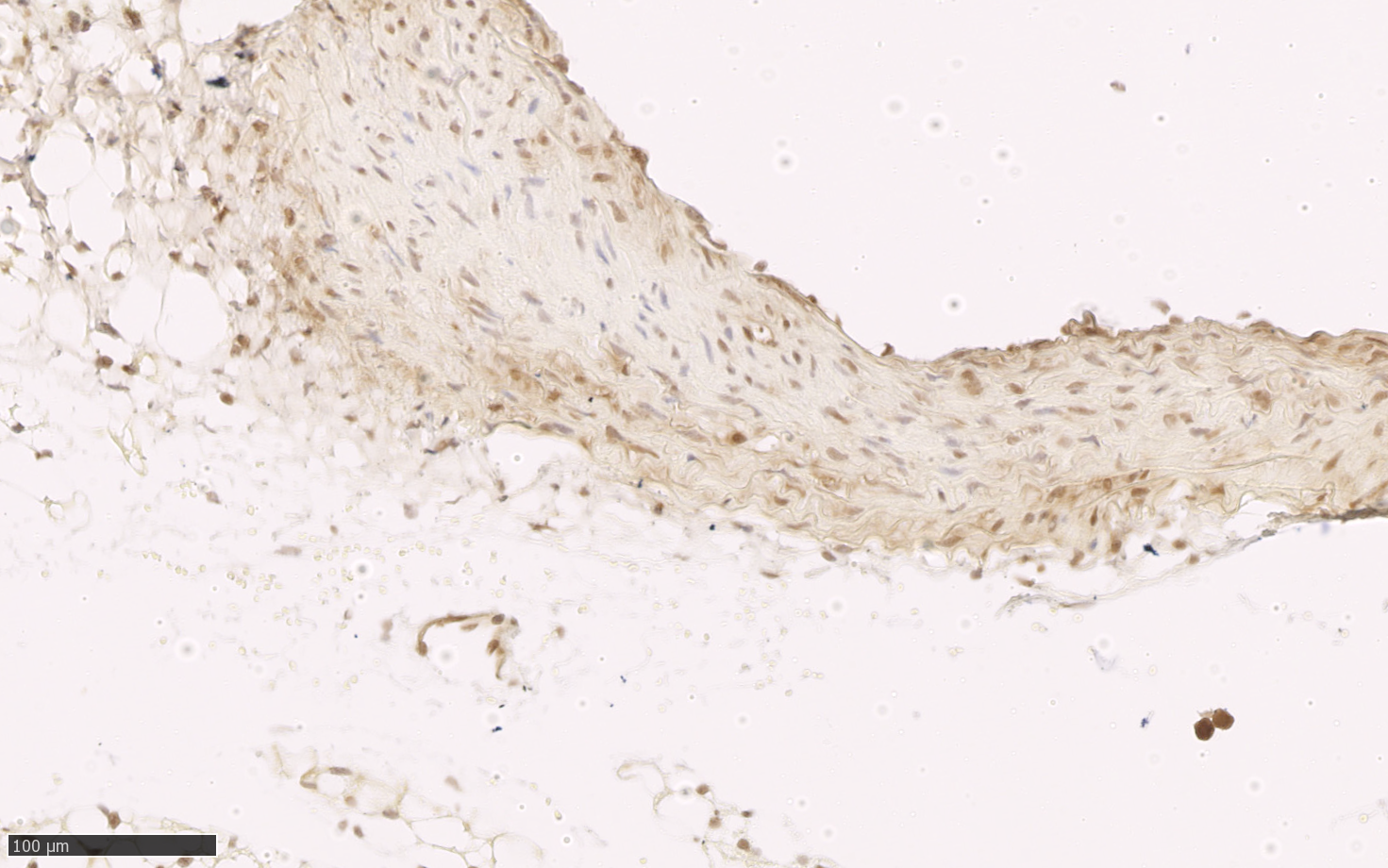

Supplement: Supplementary file 6 — Source Data Fig. 4 [file 44321_2023_9_MOESM6_ESM.zip › Figure 4/4C/ihq-gfp-asao-mfs-scr.tif]

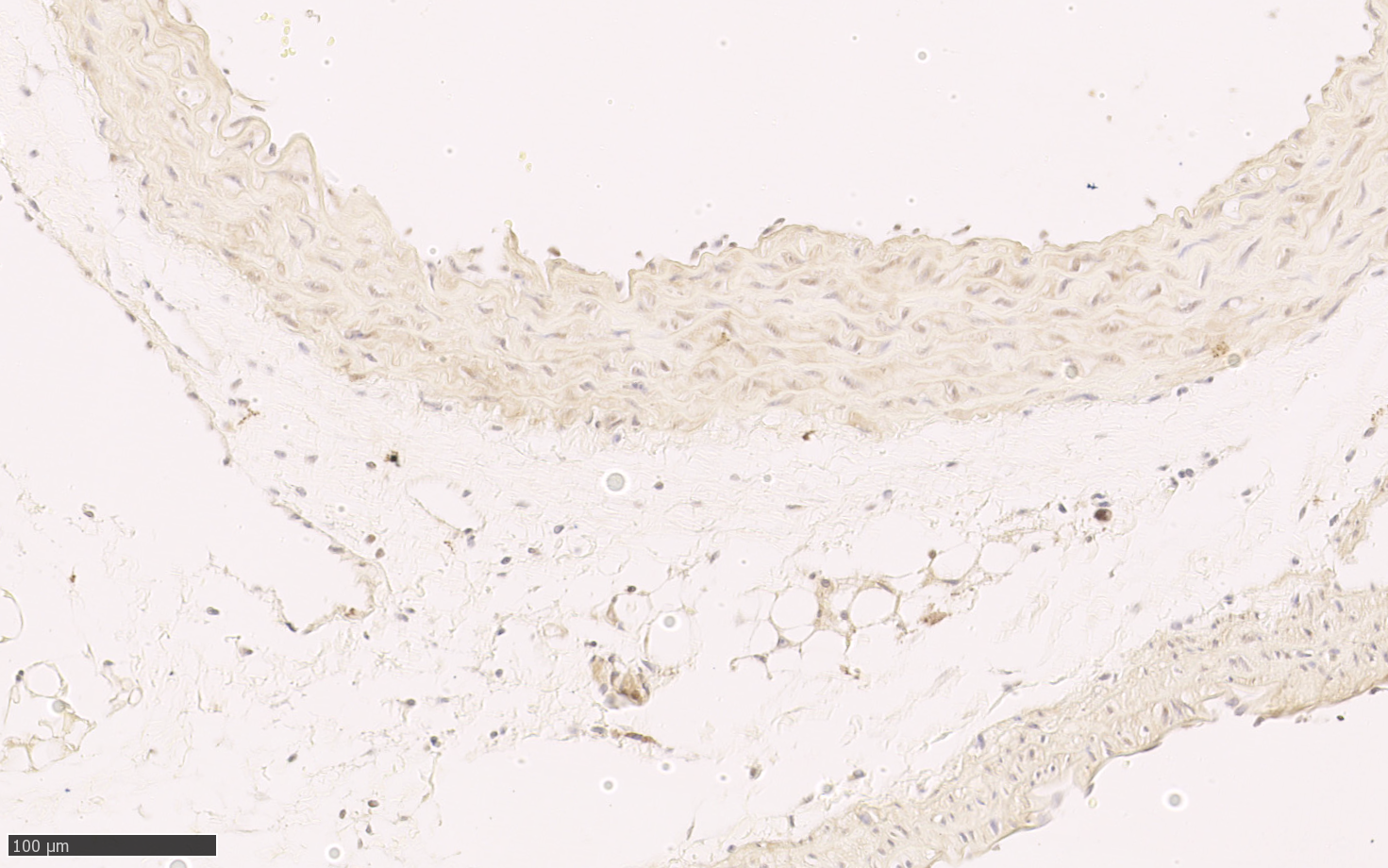

Supplement: Supplementary file 6 — Source Data Fig. 4 [file 44321_2023_9_MOESM6_ESM.zip › Figure 4/4C/ihq-gfp-asao-non-infected.tif]

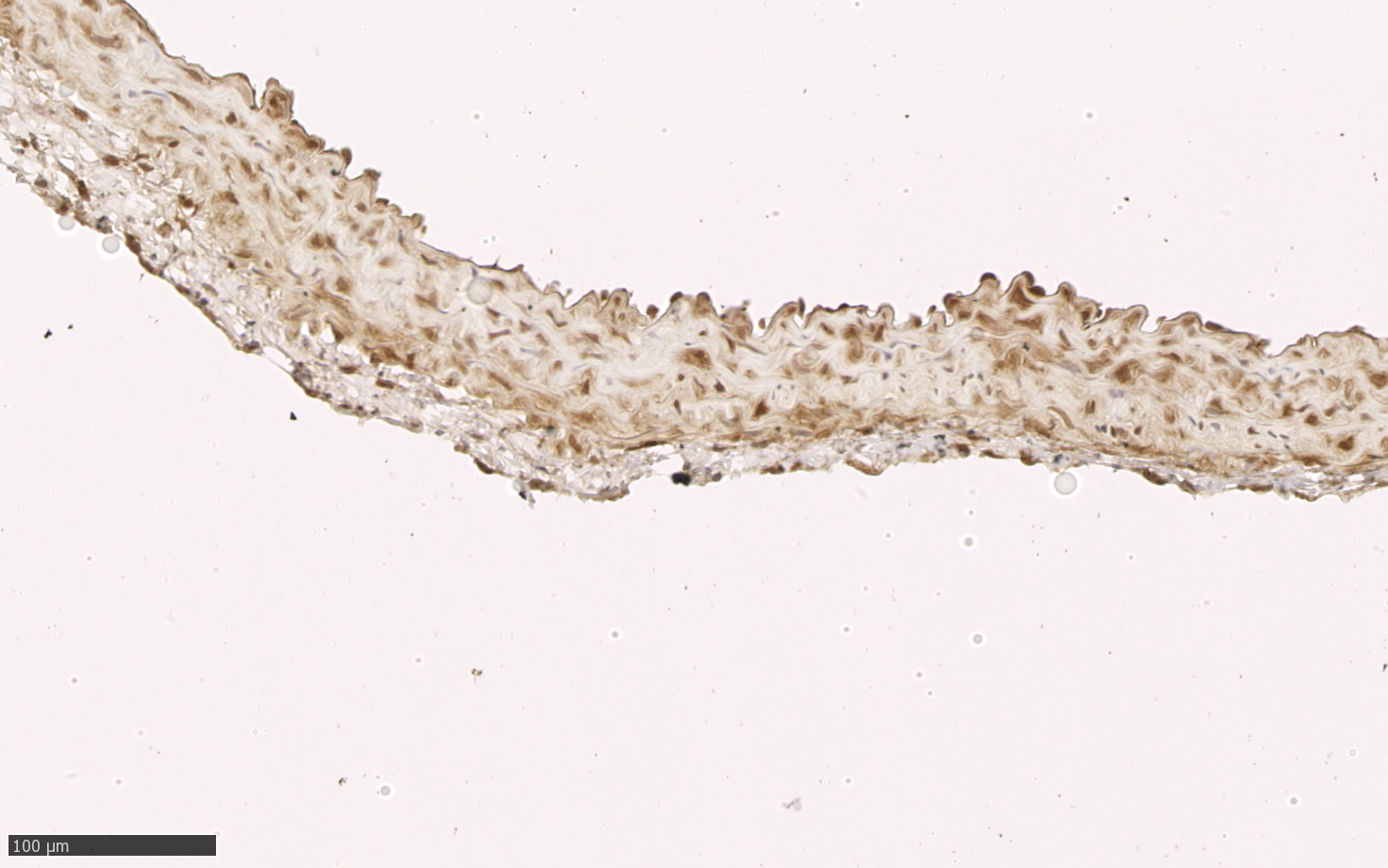

Supplement: Supplementary file 6 — Source Data Fig. 4 [file 44321_2023_9_MOESM6_ESM.zip › Figure 4/4C/ihq-gfp-asao-wt-acan.tif]

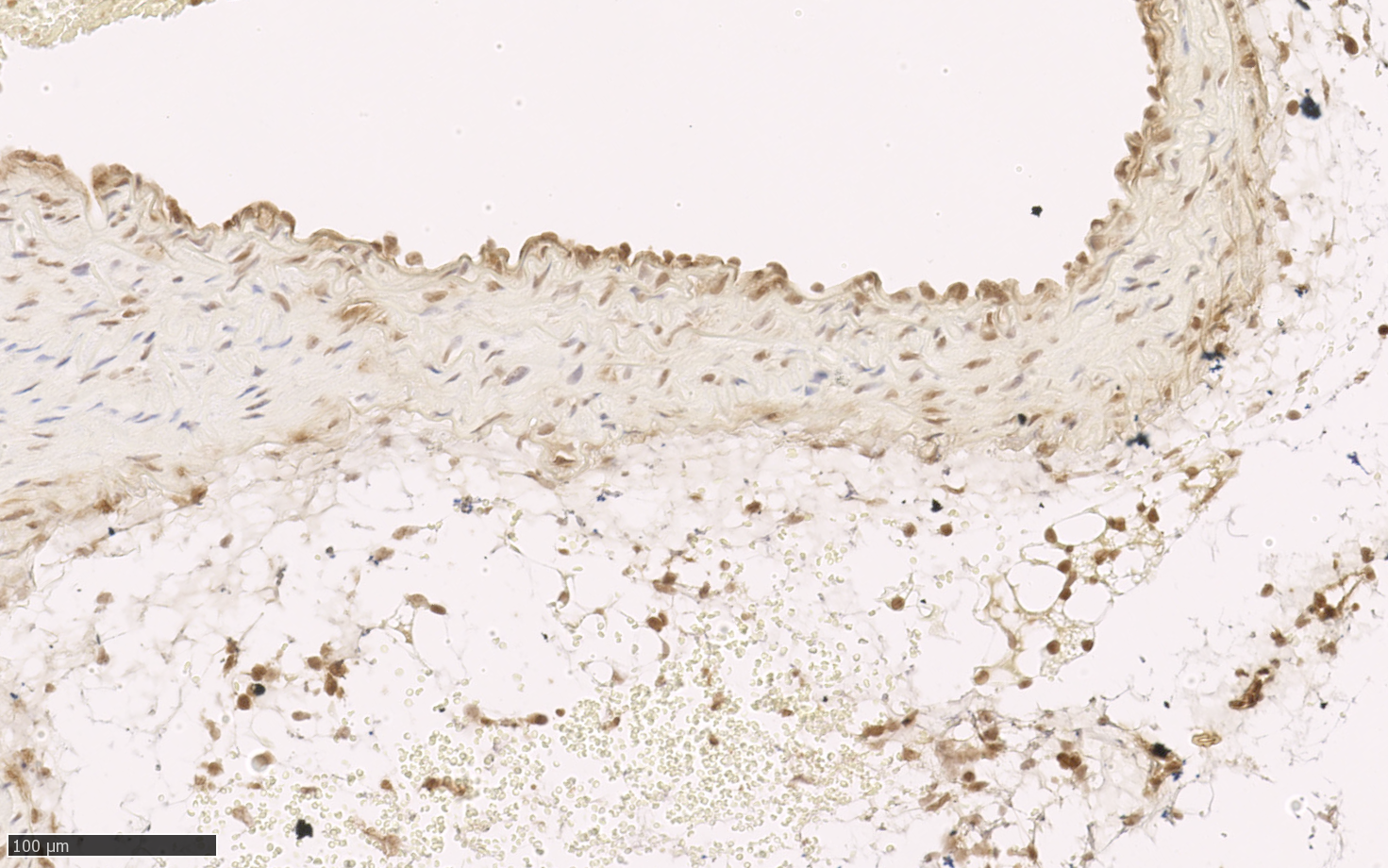

Supplement: Supplementary file 6 — Source Data Fig. 4 [file 44321_2023_9_MOESM6_ESM.zip › Figure 4/4C/ihq-gfp-asao-wt-scr.tif]

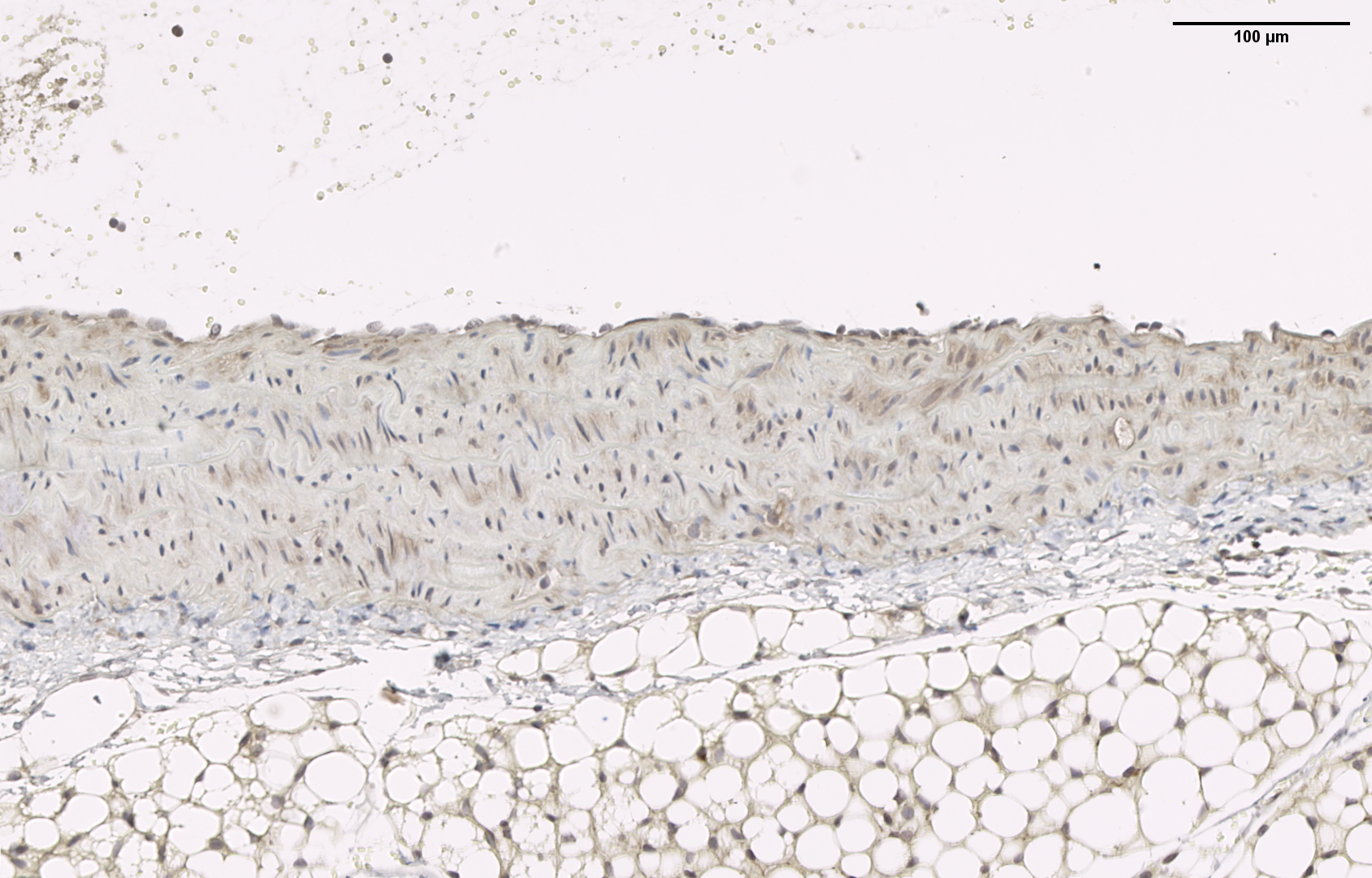

Supplement: Supplementary file 6 — Source Data Fig. 4 [file 44321_2023_9_MOESM6_ESM.zip › Figure 4/4D/ihq-acan-mfs-acan.tif]

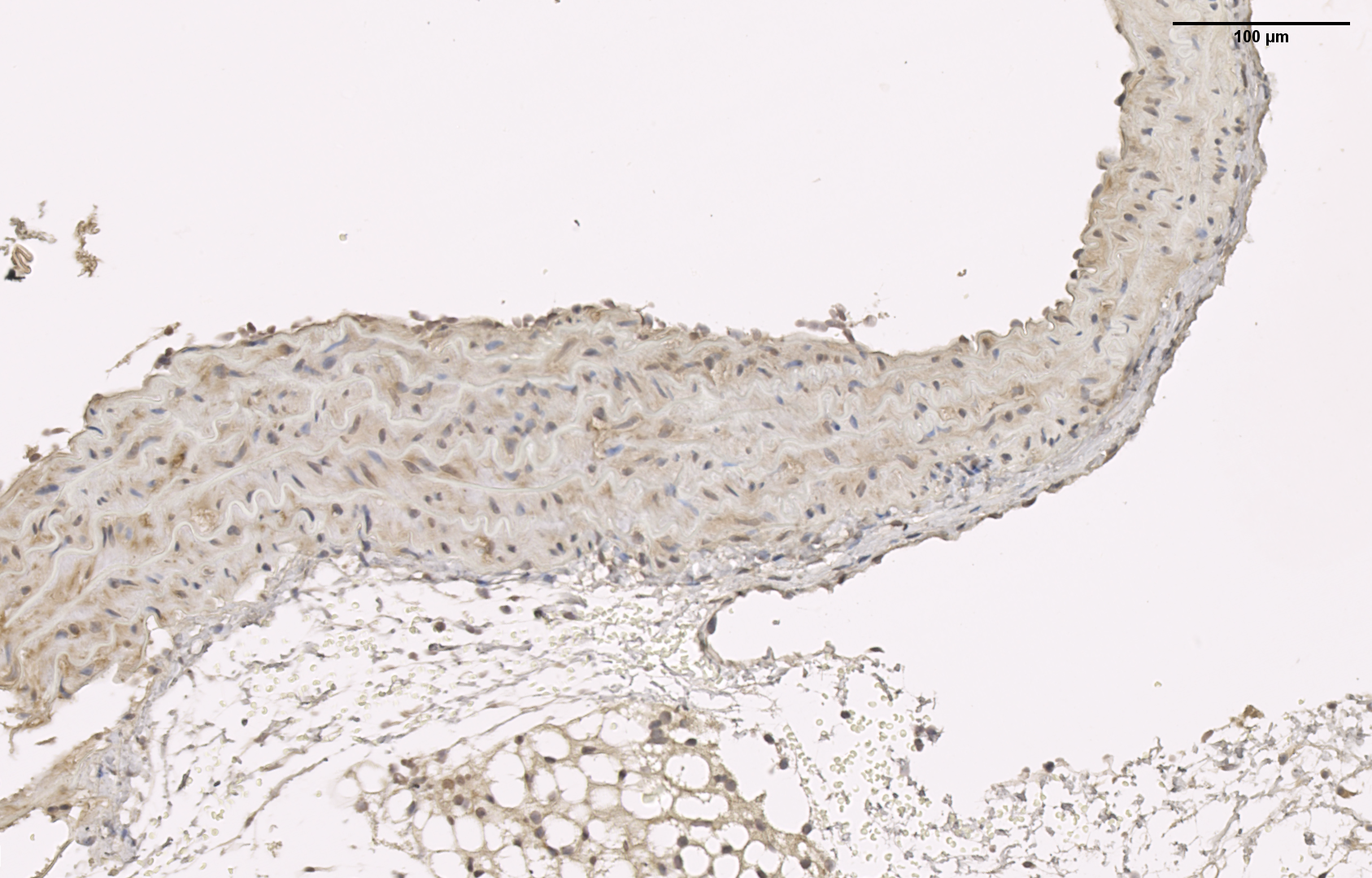

Supplement: Supplementary file 6 — Source Data Fig. 4 [file 44321_2023_9_MOESM6_ESM.zip › Figure 4/4D/ihq-acan-mfs-scr.tif]

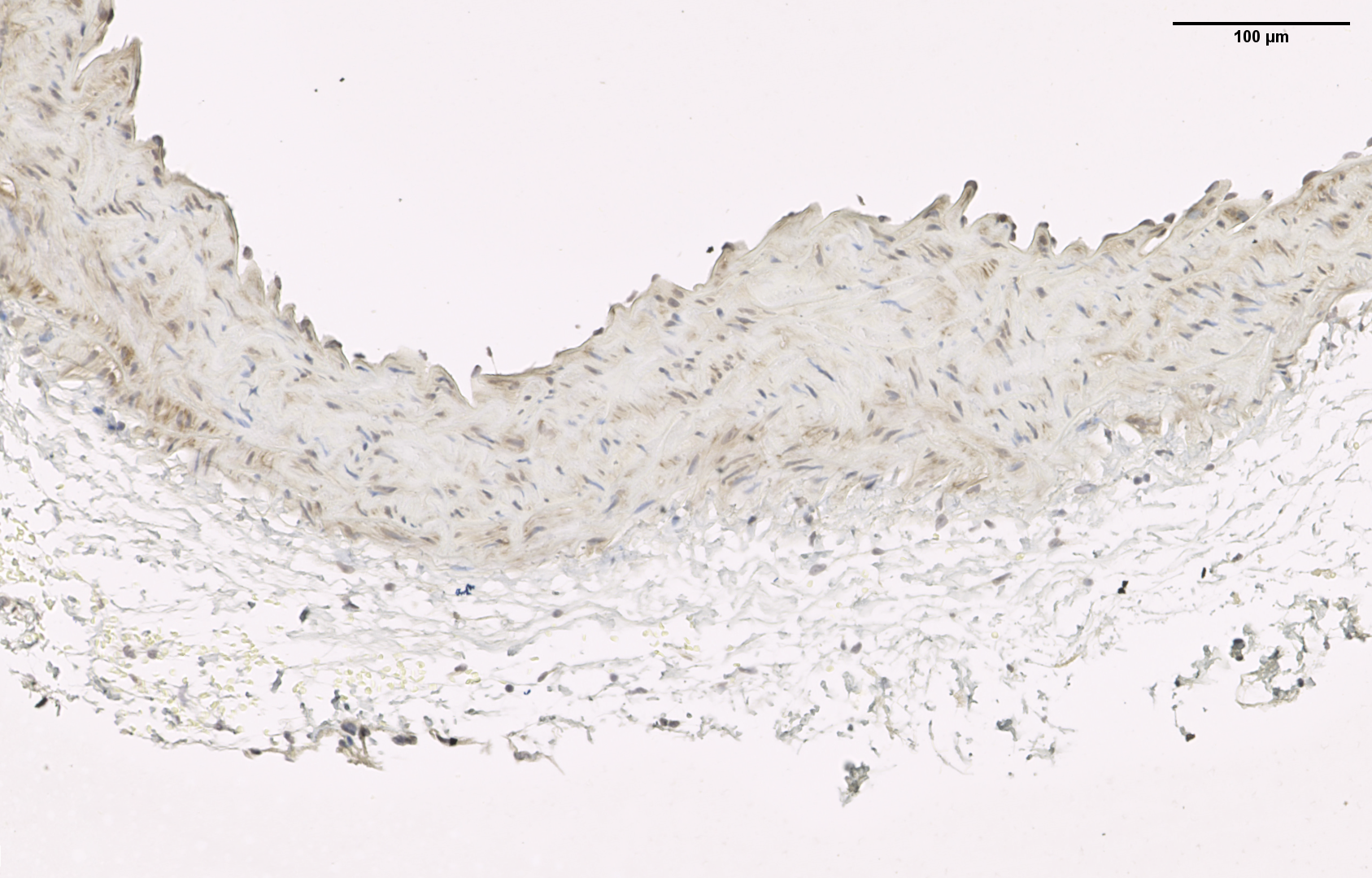

Supplement: Supplementary file 6 — Source Data Fig. 4 [file 44321_2023_9_MOESM6_ESM.zip › Figure 4/4D/ihq-acan-wt-acan.tif]

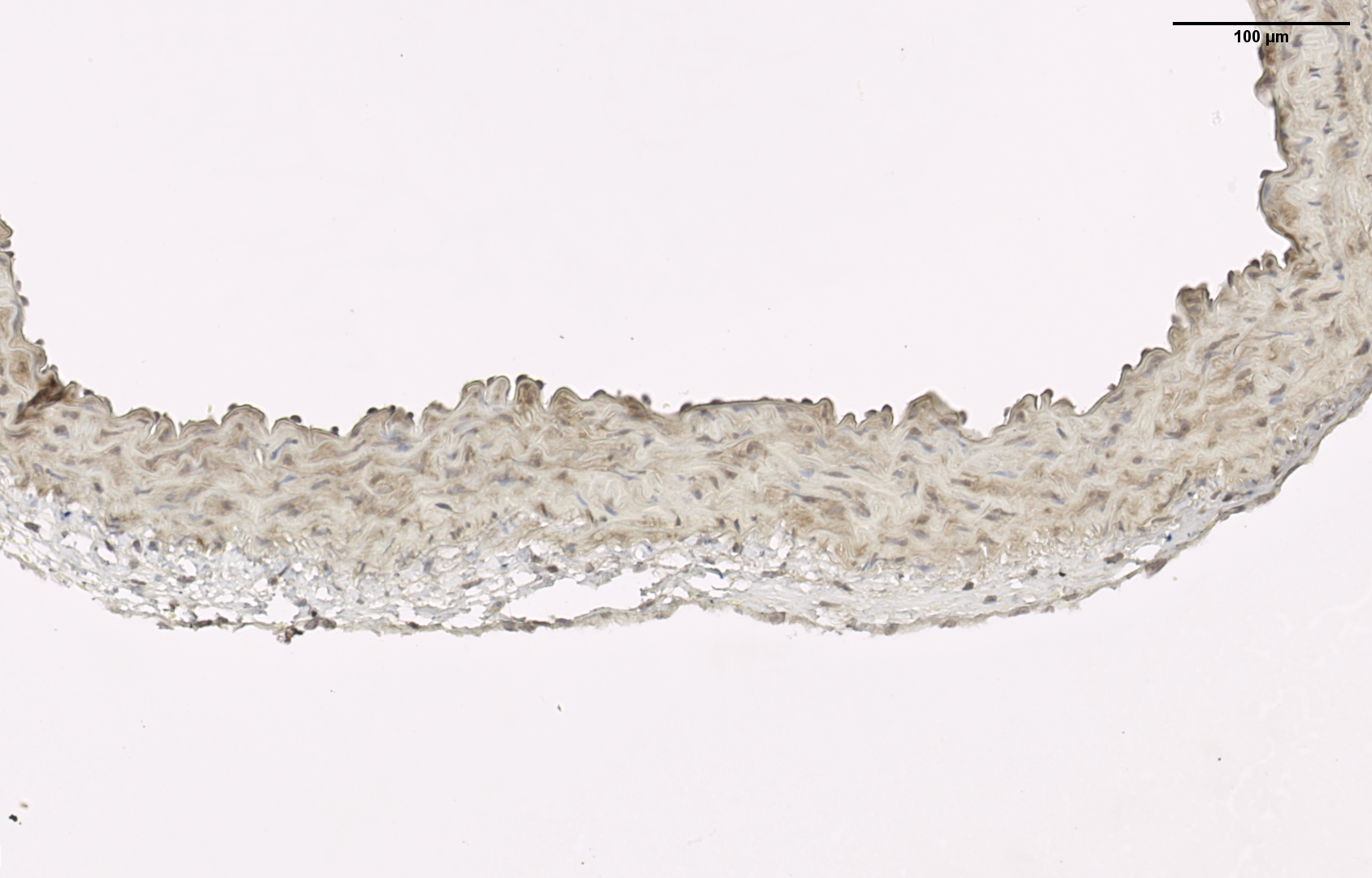

Supplement: Supplementary file 6 — Source Data Fig. 4 [file 44321_2023_9_MOESM6_ESM.zip › Figure 4/4D/ihq-acan-wt-scr.tif]

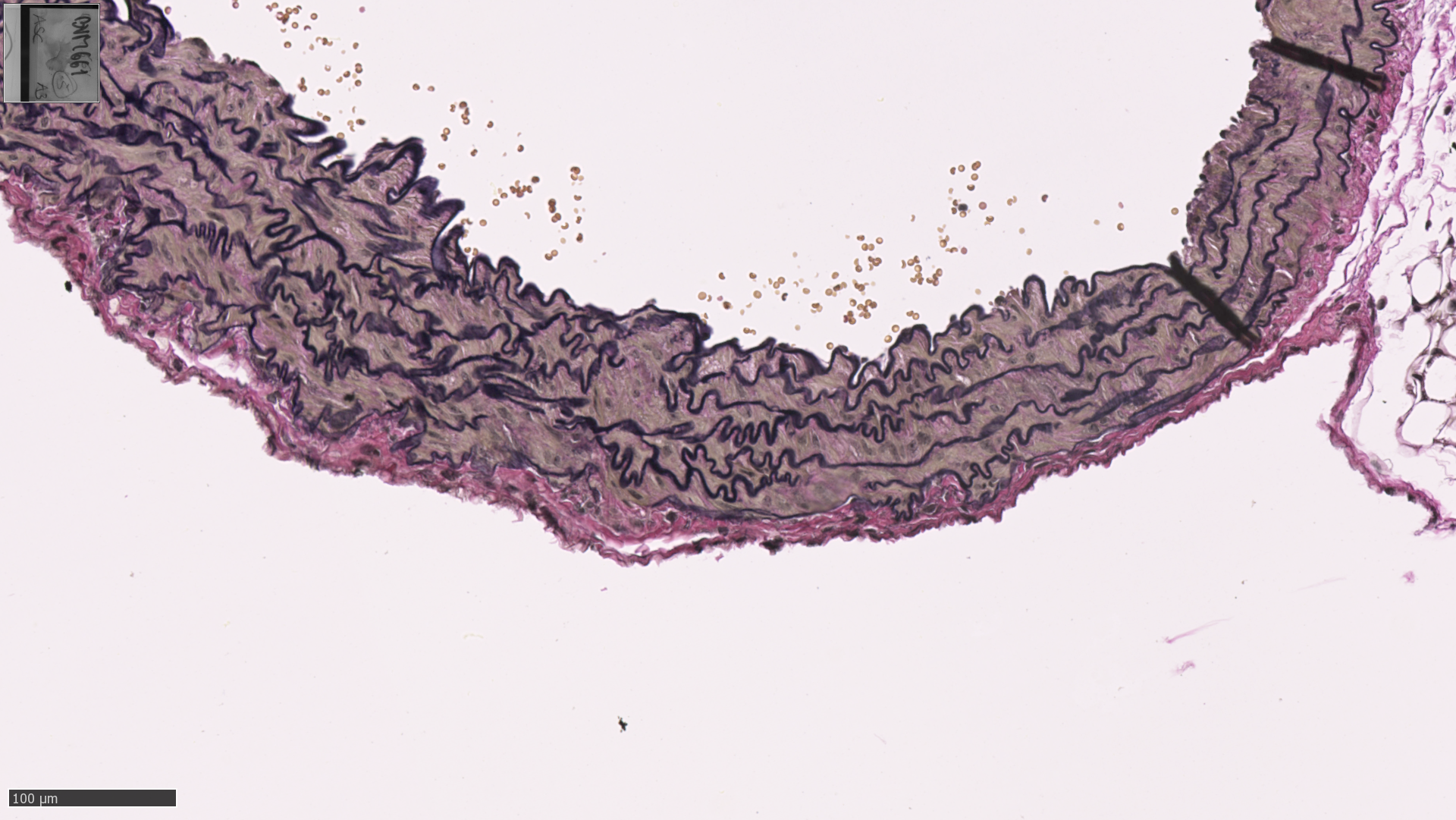

Supplement: Supplementary file 7 — Source Data Fig. 5 [file 44321_2023_9_MOESM7_ESM.zip › Figure 5/5A/evg-mfs-acan.tif]

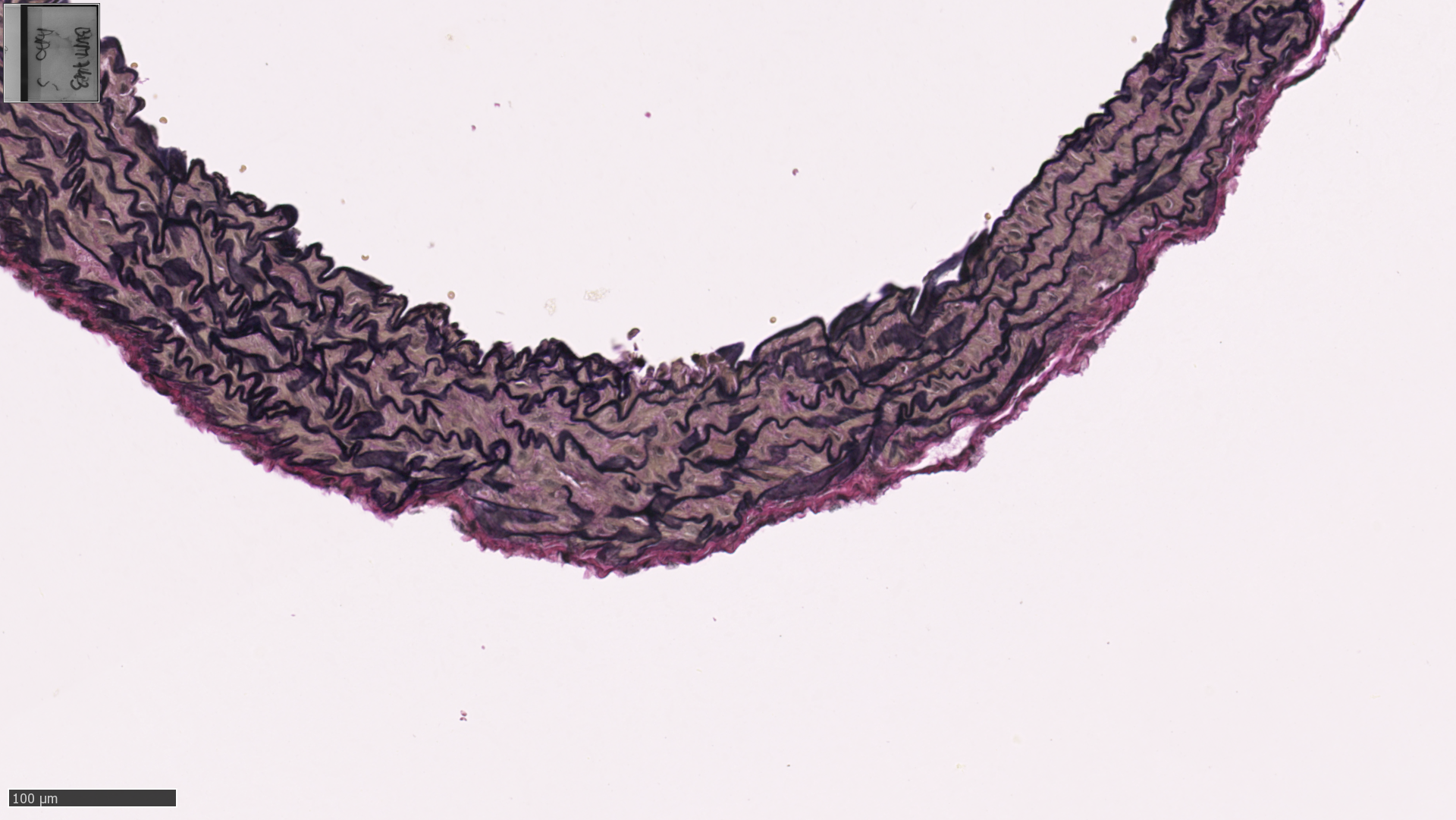

Supplement: Supplementary file 7 — Source Data Fig. 5 [file 44321_2023_9_MOESM7_ESM.zip › Figure 5/5A/evg-mfs-scr.tif]

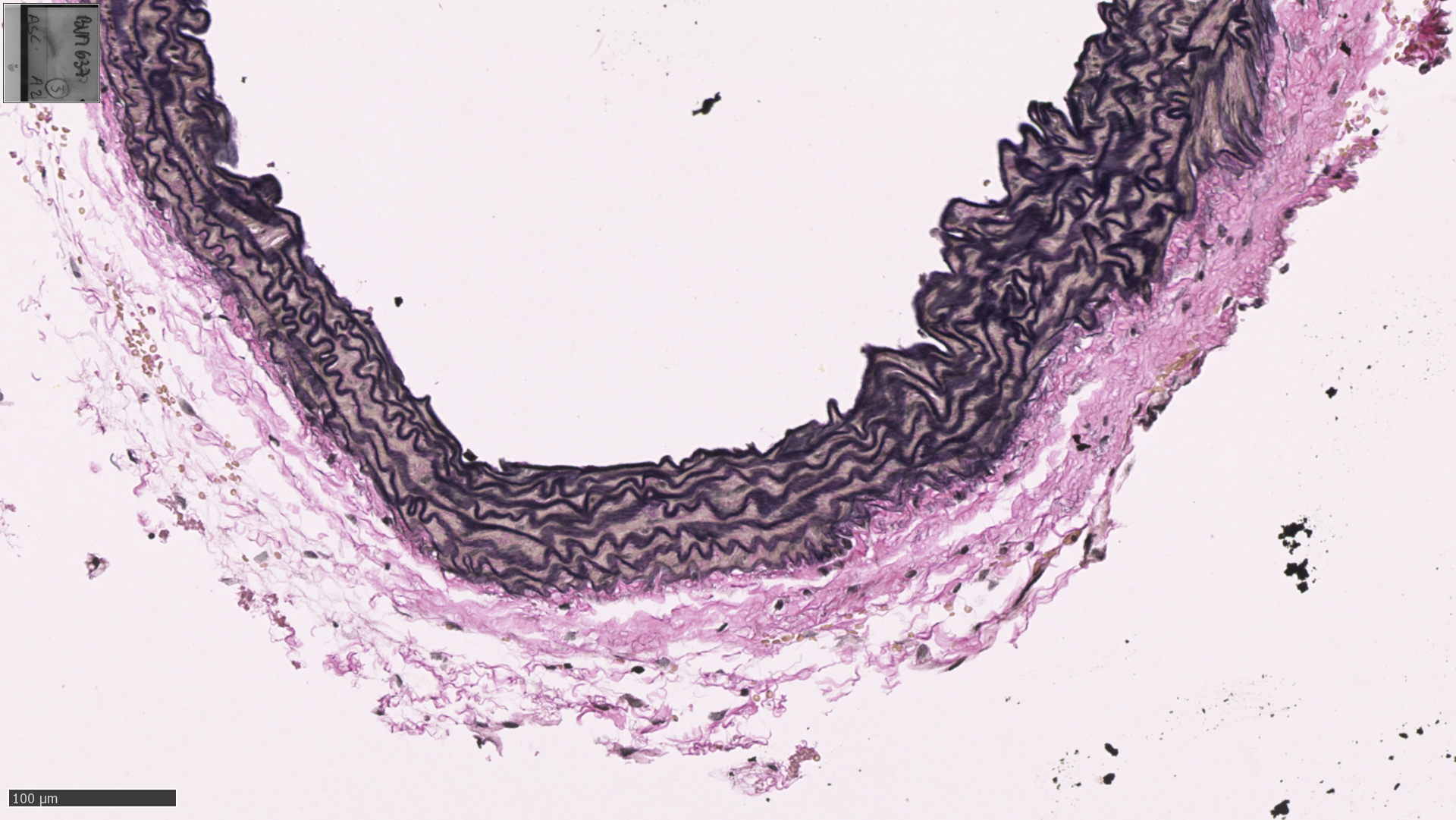

Supplement: Supplementary file 7 — Source Data Fig. 5 [file 44321_2023_9_MOESM7_ESM.zip › Figure 5/5A/evg-wt-acan.tif]

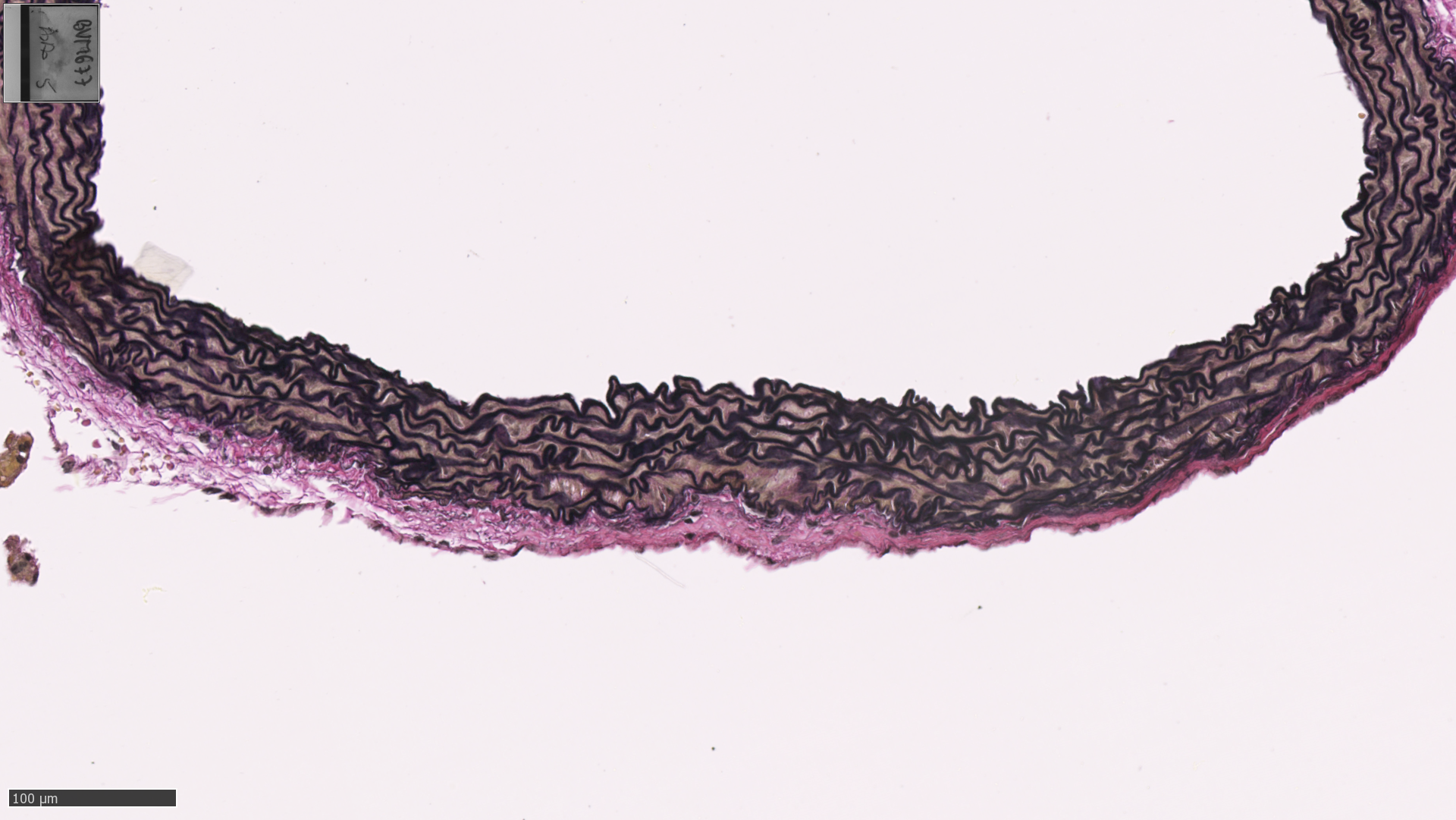

Supplement: Supplementary file 7 — Source Data Fig. 5 [file 44321_2023_9_MOESM7_ESM.zip › Figure 5/5A/evg-wt-scr.tif]

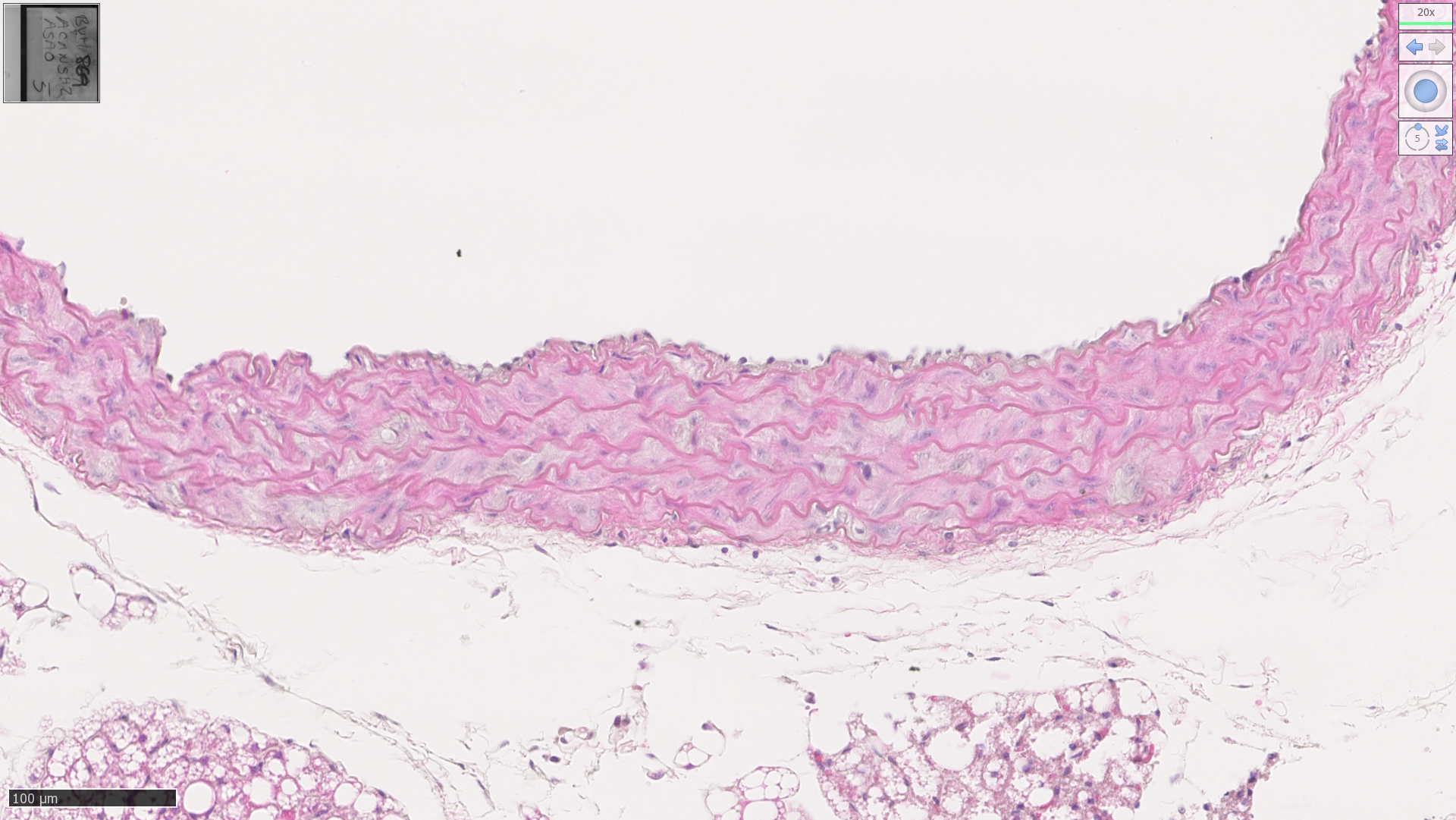

Supplement: Supplementary file 7 — Source Data Fig. 5 [file 44321_2023_9_MOESM7_ESM.zip › Figure 5/5A/h&e-mfs-acan.tif]

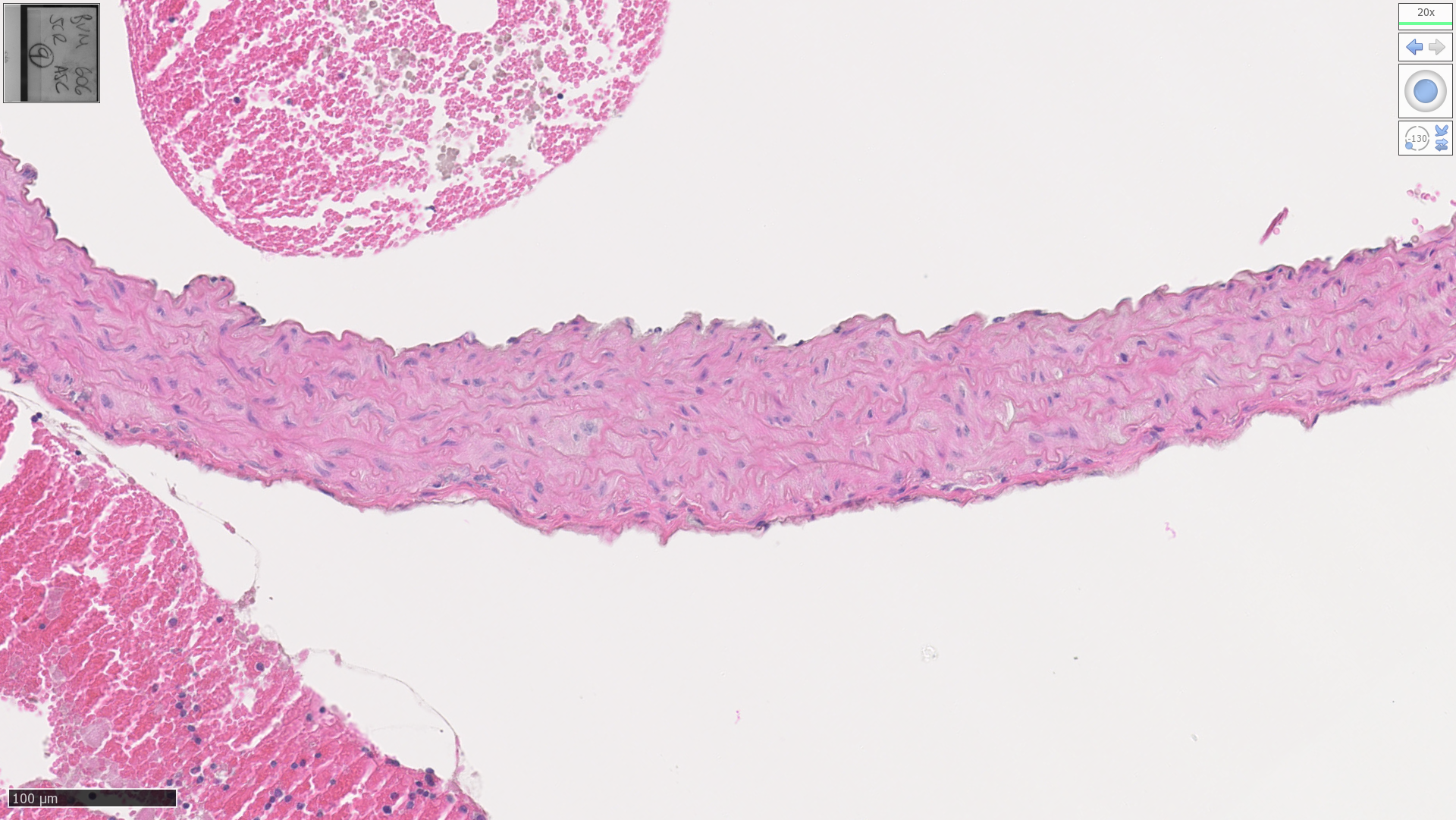

Supplement: Supplementary file 7 — Source Data Fig. 5 [file 44321_2023_9_MOESM7_ESM.zip › Figure 5/5A/h&e-mfs-scr.tif]

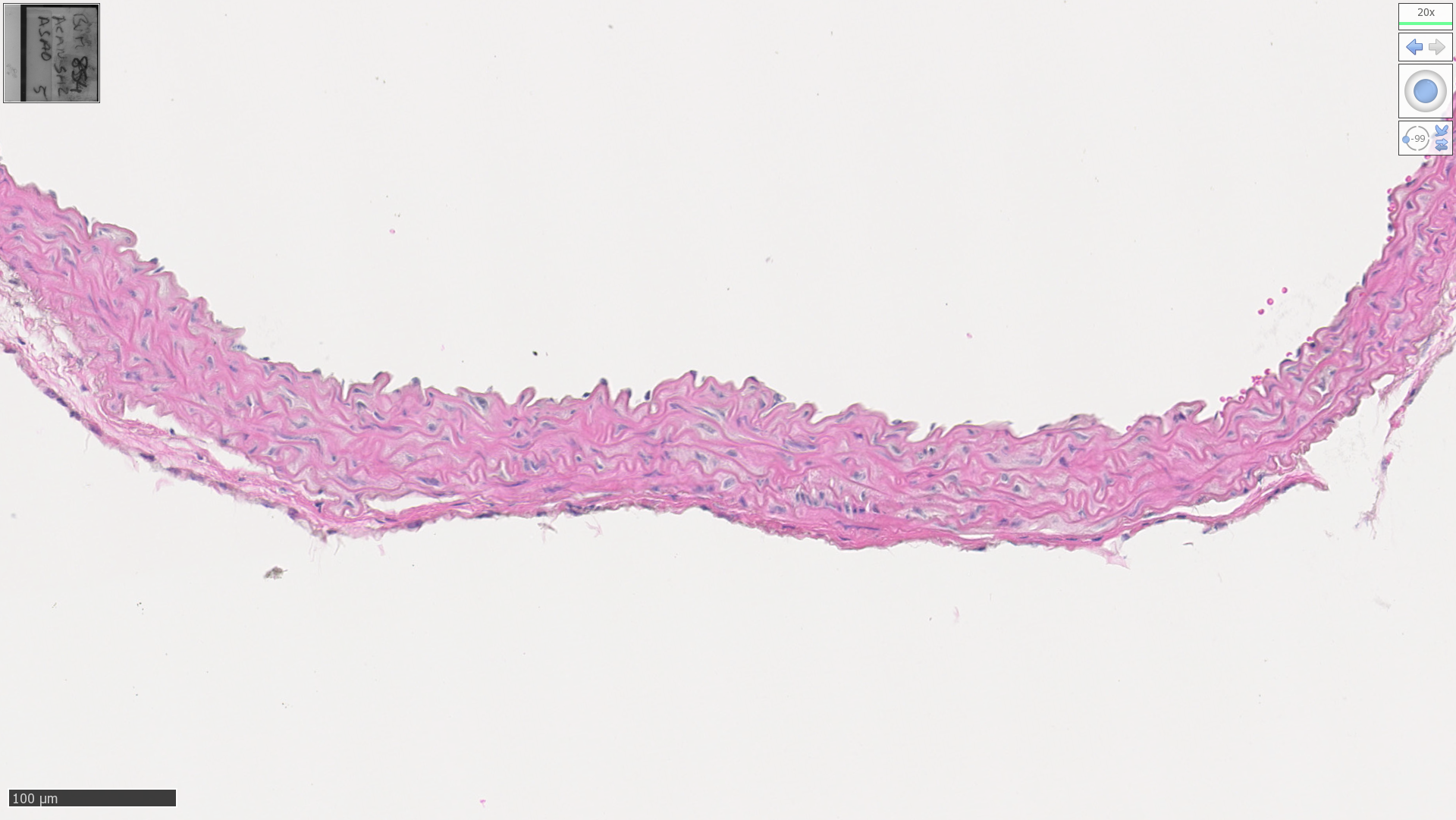

Supplement: Supplementary file 7 — Source Data Fig. 5 [file 44321_2023_9_MOESM7_ESM.zip › Figure 5/5A/h&e-wt-acan.tif]

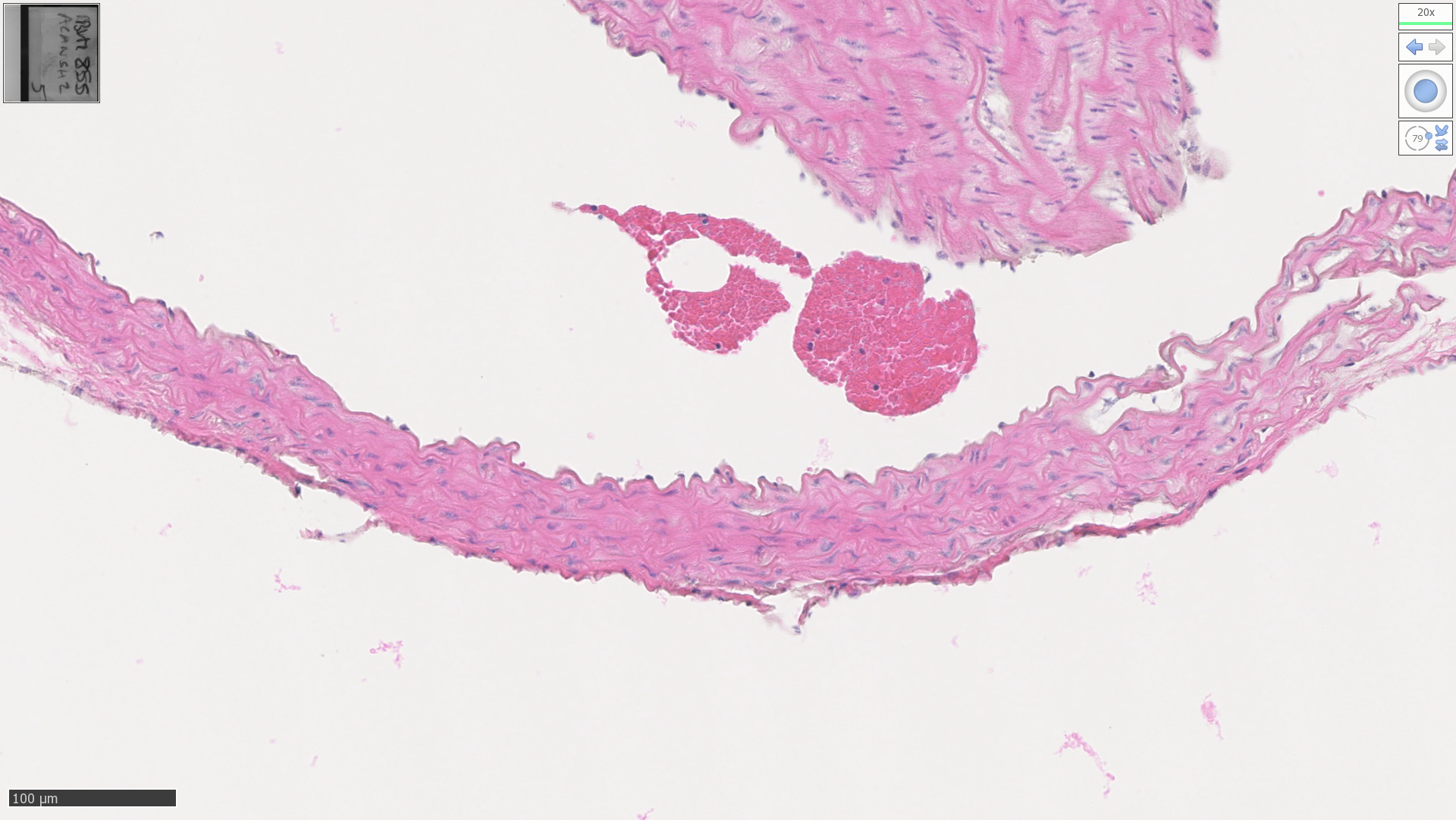

Supplement: Supplementary file 7 — Source Data Fig. 5 [file 44321_2023_9_MOESM7_ESM.zip › Figure 5/5A/h&e-wt-scr.tif]

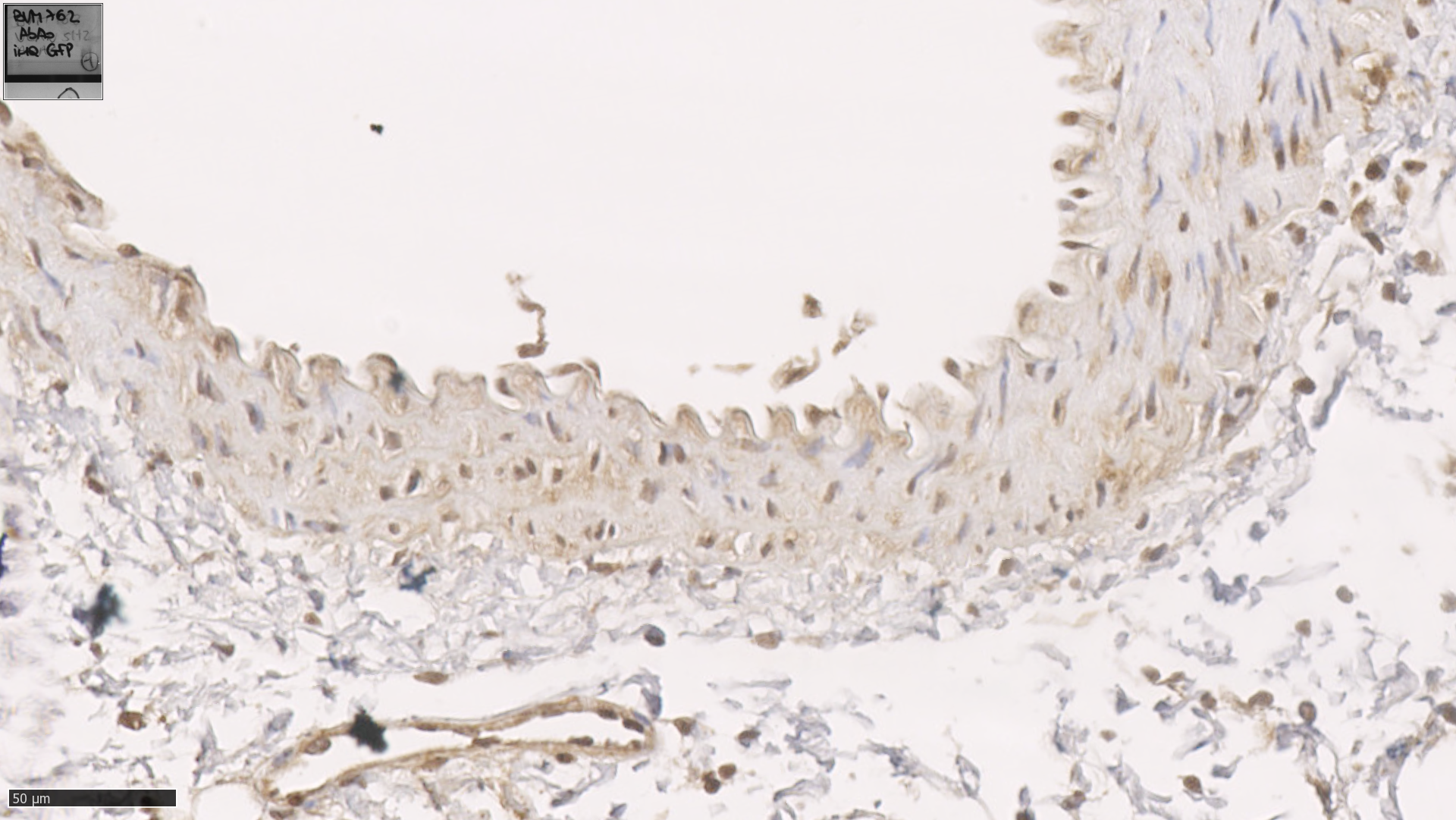

Supplement: Supplementary file 8 — Source Data Fig. 6 [file 44321_2023_9_MOESM8_ESM.zip › Figure 6/6C/ihq-gfp-abao-mfs-scr.tif]

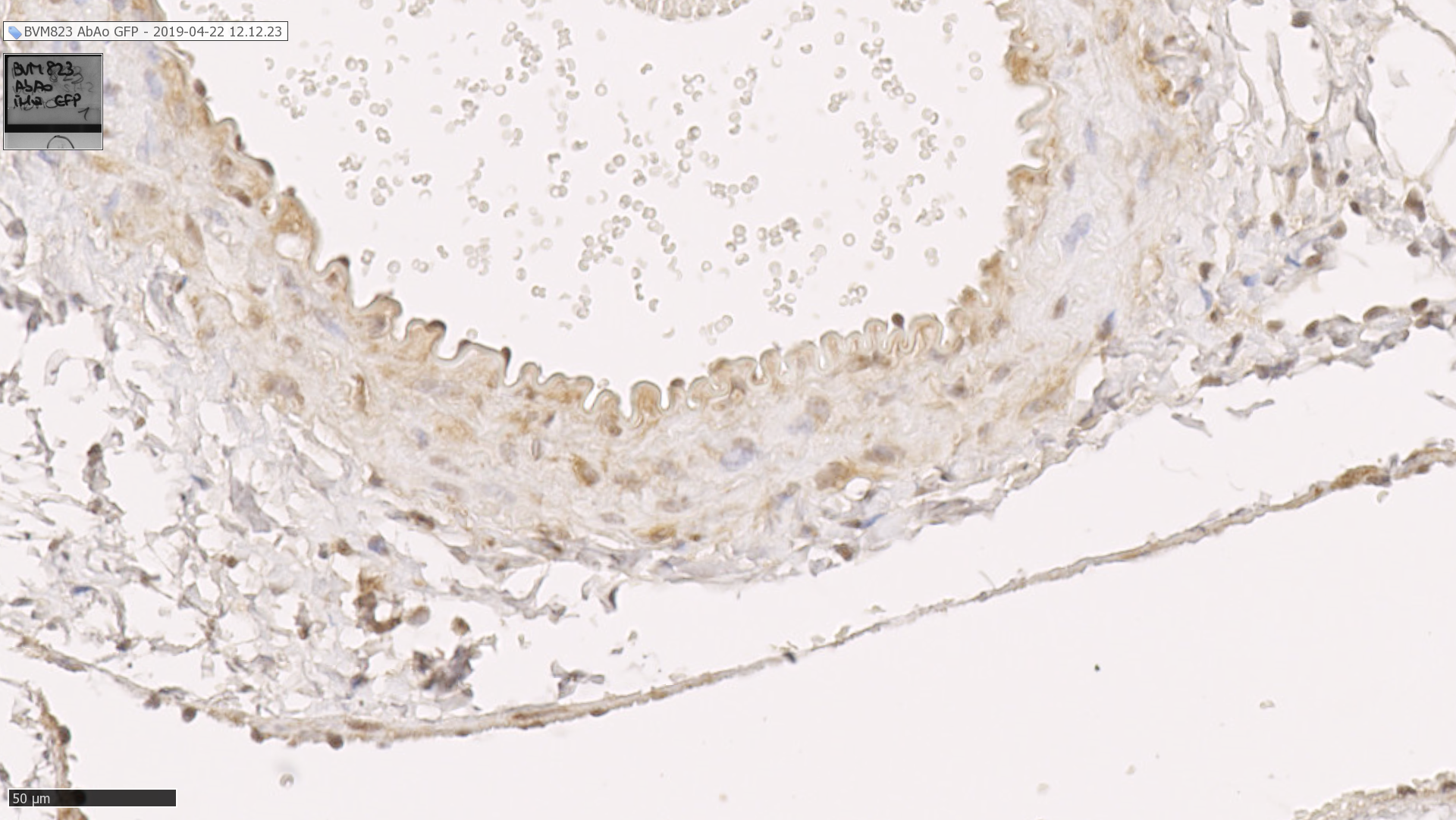

Supplement: Supplementary file 8 — Source Data Fig. 6 [file 44321_2023_9_MOESM8_ESM.zip › Figure 6/6C/ihq-gfp-abao-mfs-vcan.tif]

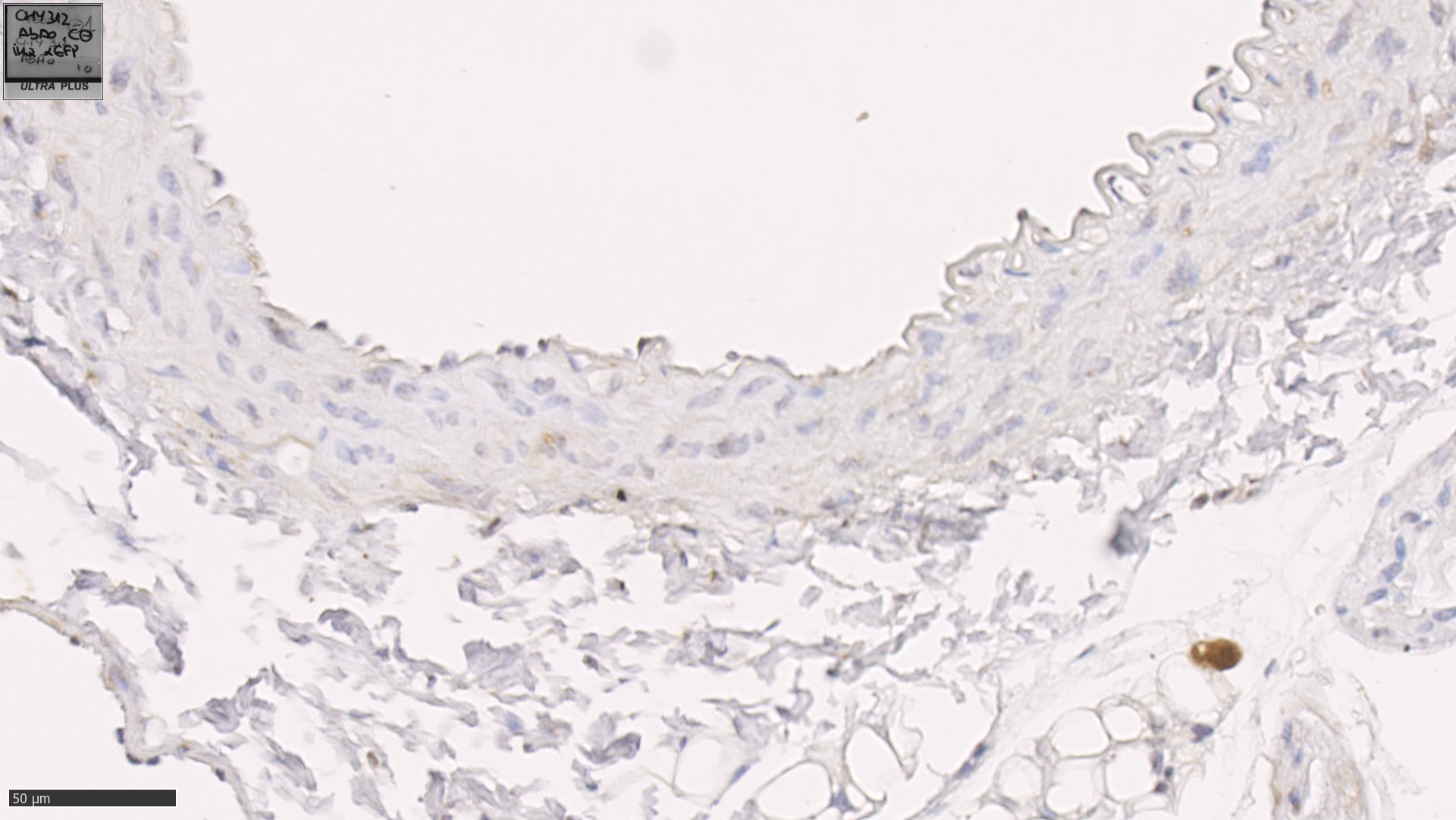

Supplement: Supplementary file 8 — Source Data Fig. 6 [file 44321_2023_9_MOESM8_ESM.zip › Figure 6/6C/ihq-gfp-abao-uninfected.tif]

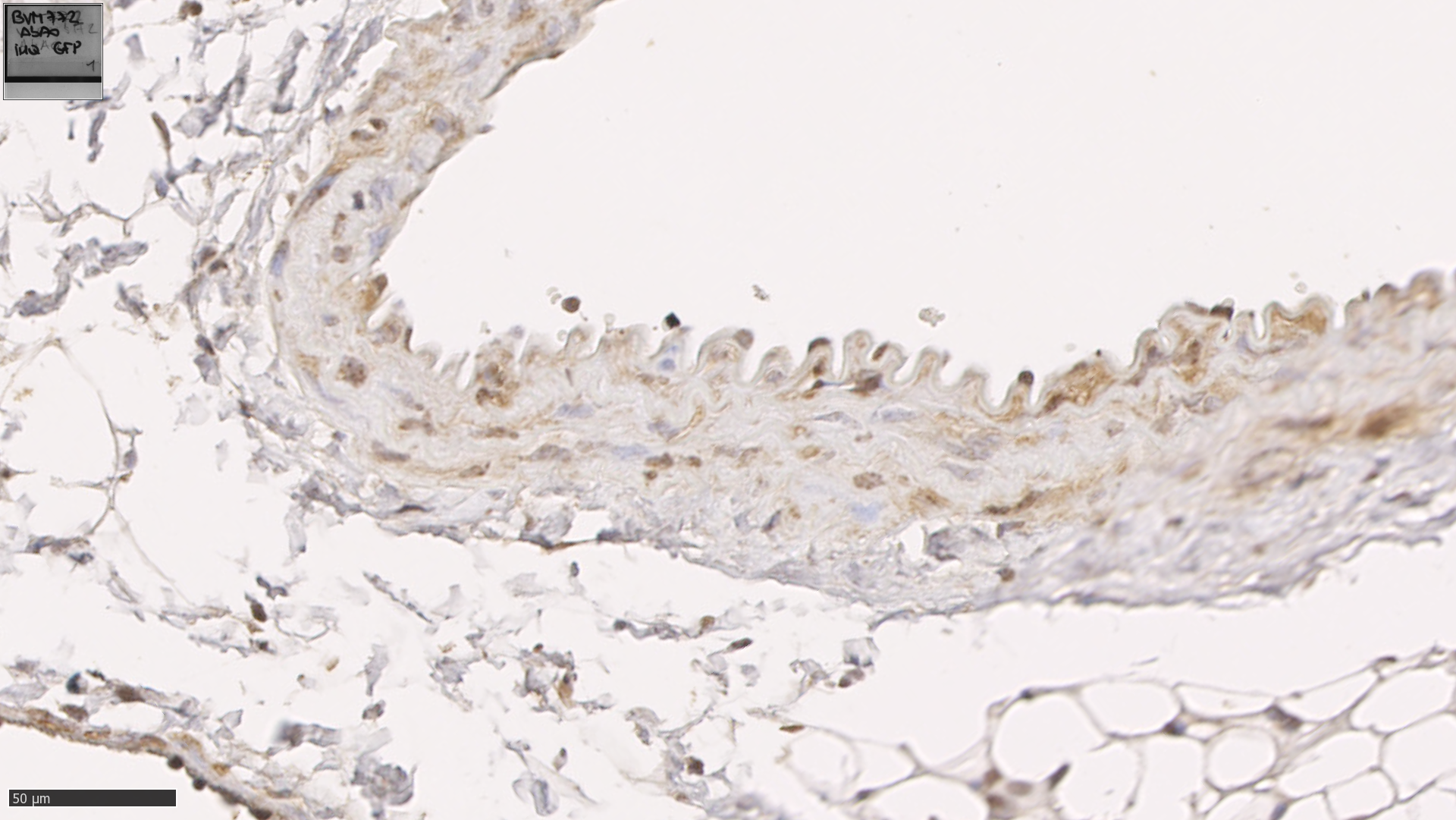

Supplement: Supplementary file 8 — Source Data Fig. 6 [file 44321_2023_9_MOESM8_ESM.zip › Figure 6/6C/ihq-gfp-abao-wt-scr.tif]

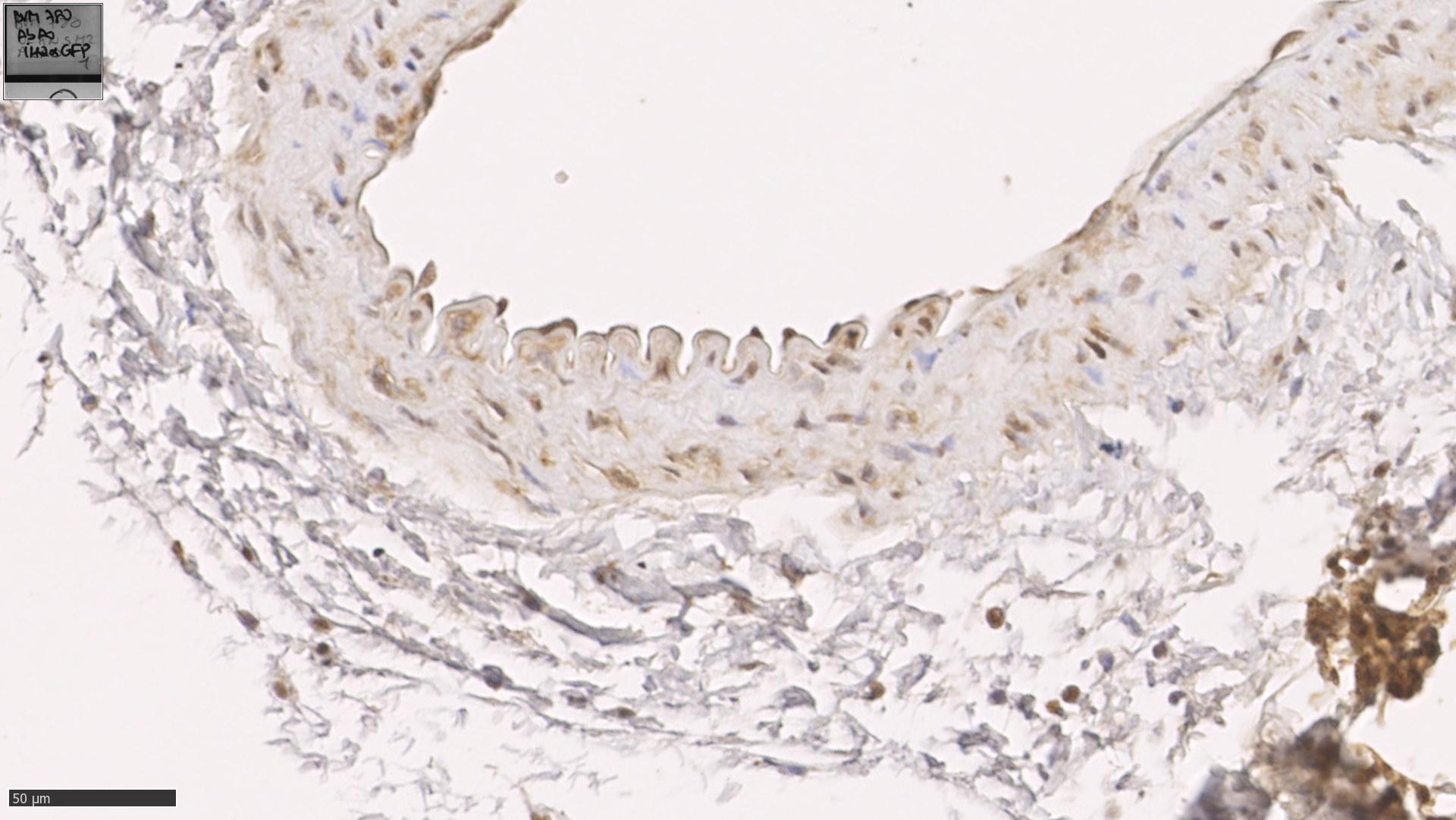

Supplement: Supplementary file 8 — Source Data Fig. 6 [file 44321_2023_9_MOESM8_ESM.zip › Figure 6/6C/ihq-gfp-abao-wt-vcan.tif]

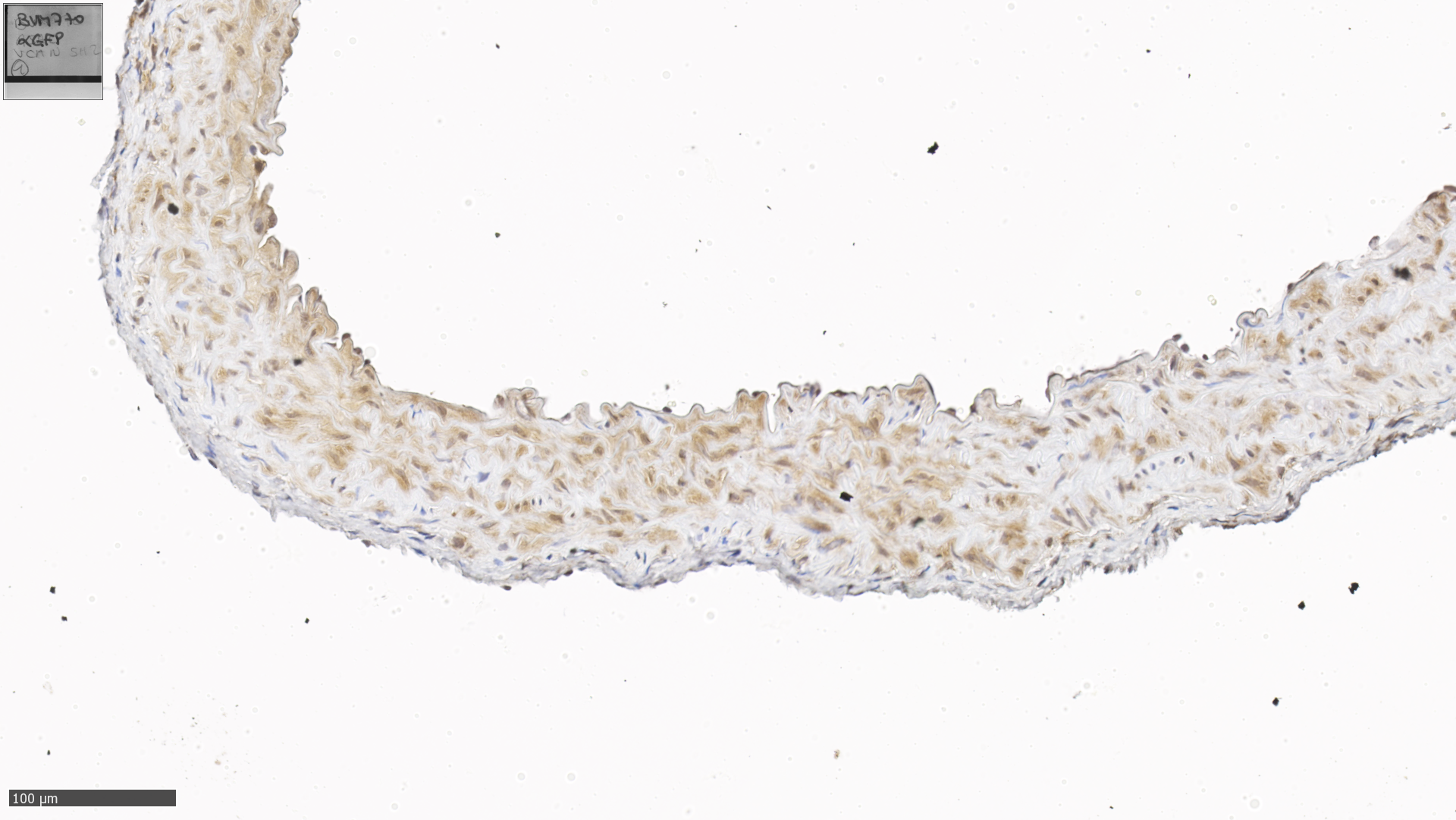

Supplement: Supplementary file 8 — Source Data Fig. 6 [file 44321_2023_9_MOESM8_ESM.zip › Figure 6/6C/ihq-gfp-asao-mfs-scr.tif]

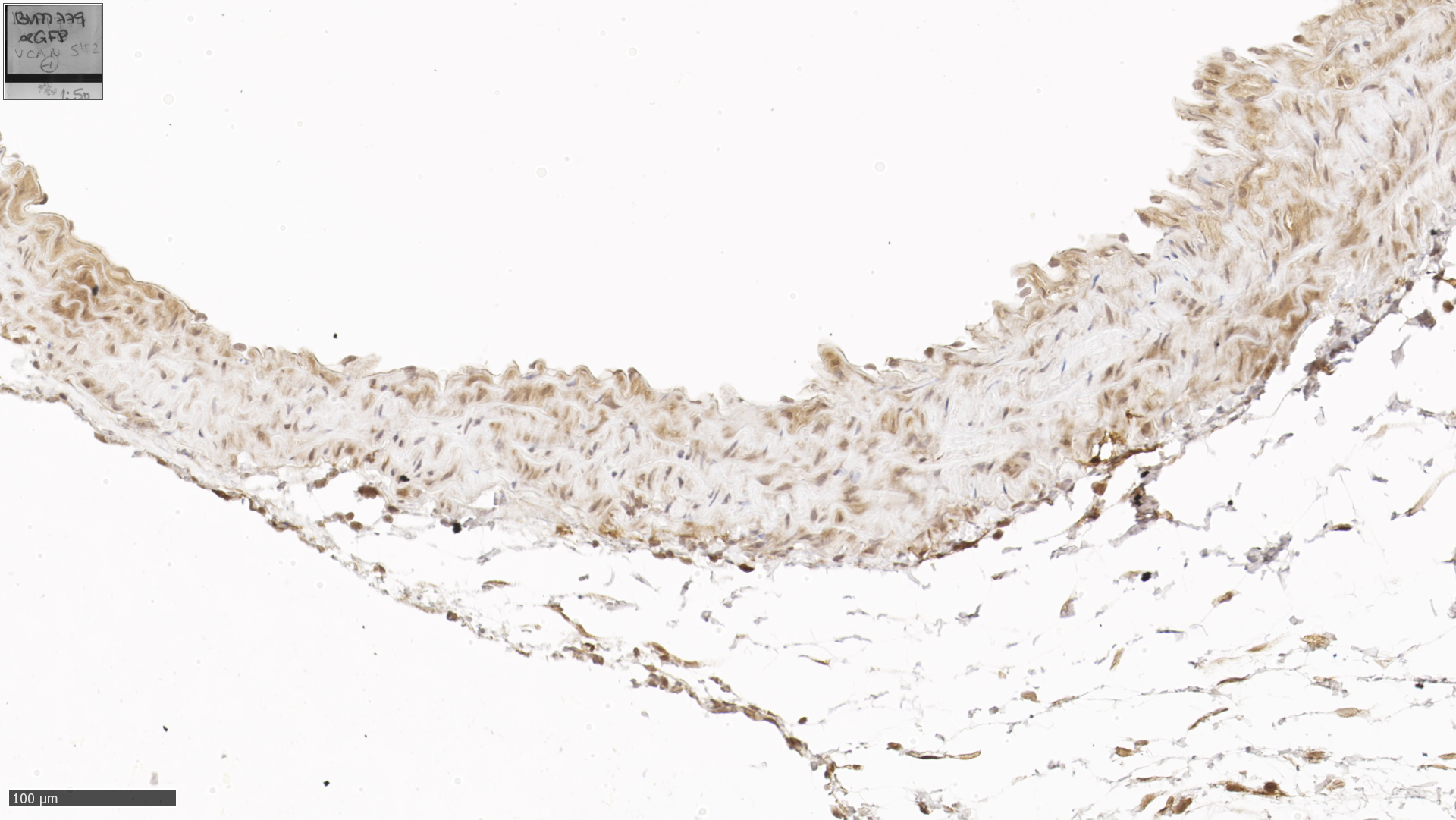

Supplement: Supplementary file 8 — Source Data Fig. 6 [file 44321_2023_9_MOESM8_ESM.zip › Figure 6/6C/ihq-gfp-asao-mfs-vcan.tif]

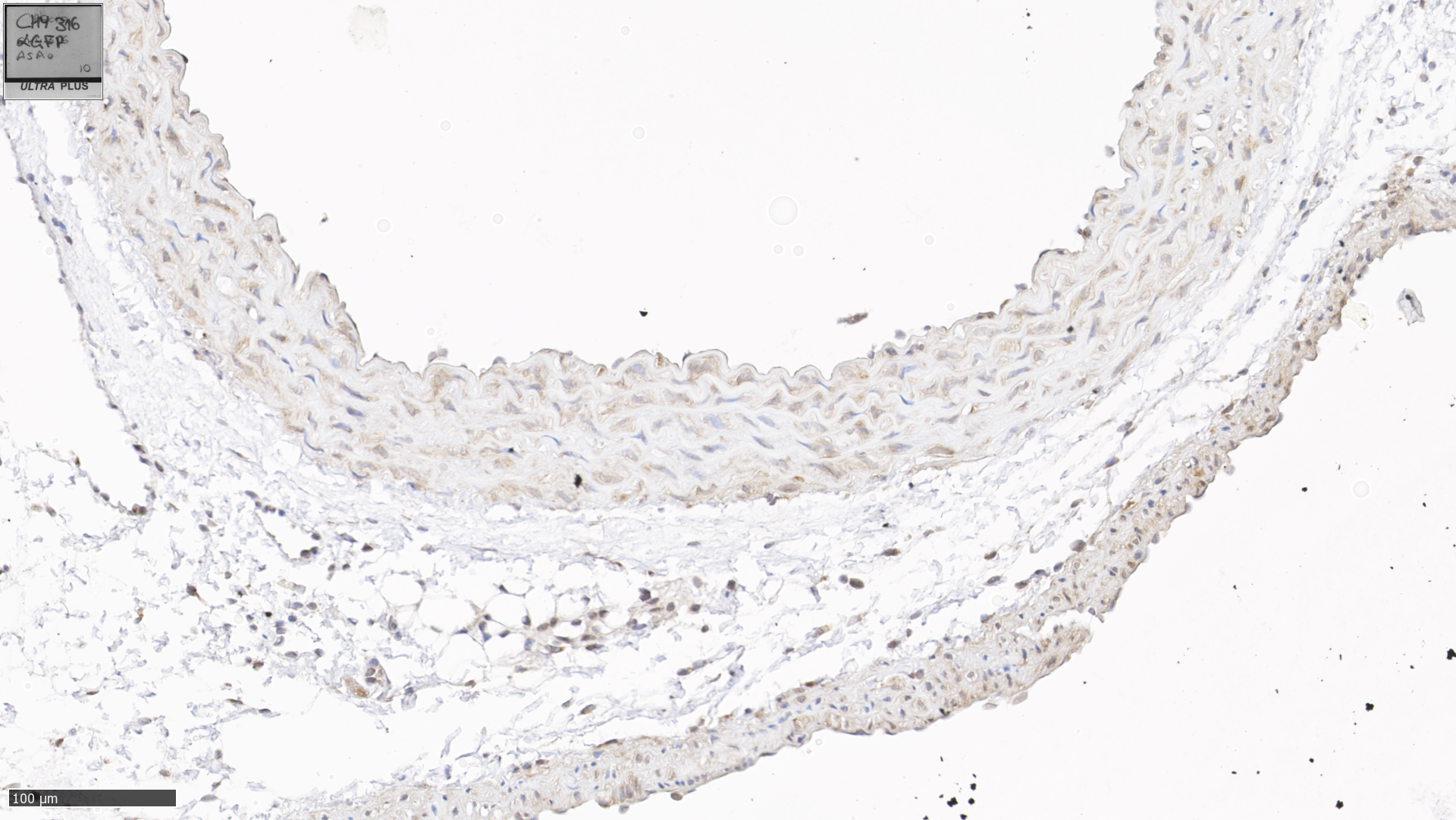

Supplement: Supplementary file 8 — Source Data Fig. 6 [file 44321_2023_9_MOESM8_ESM.zip › Figure 6/6C/ihq-gfp-asao-uninfected.tif]

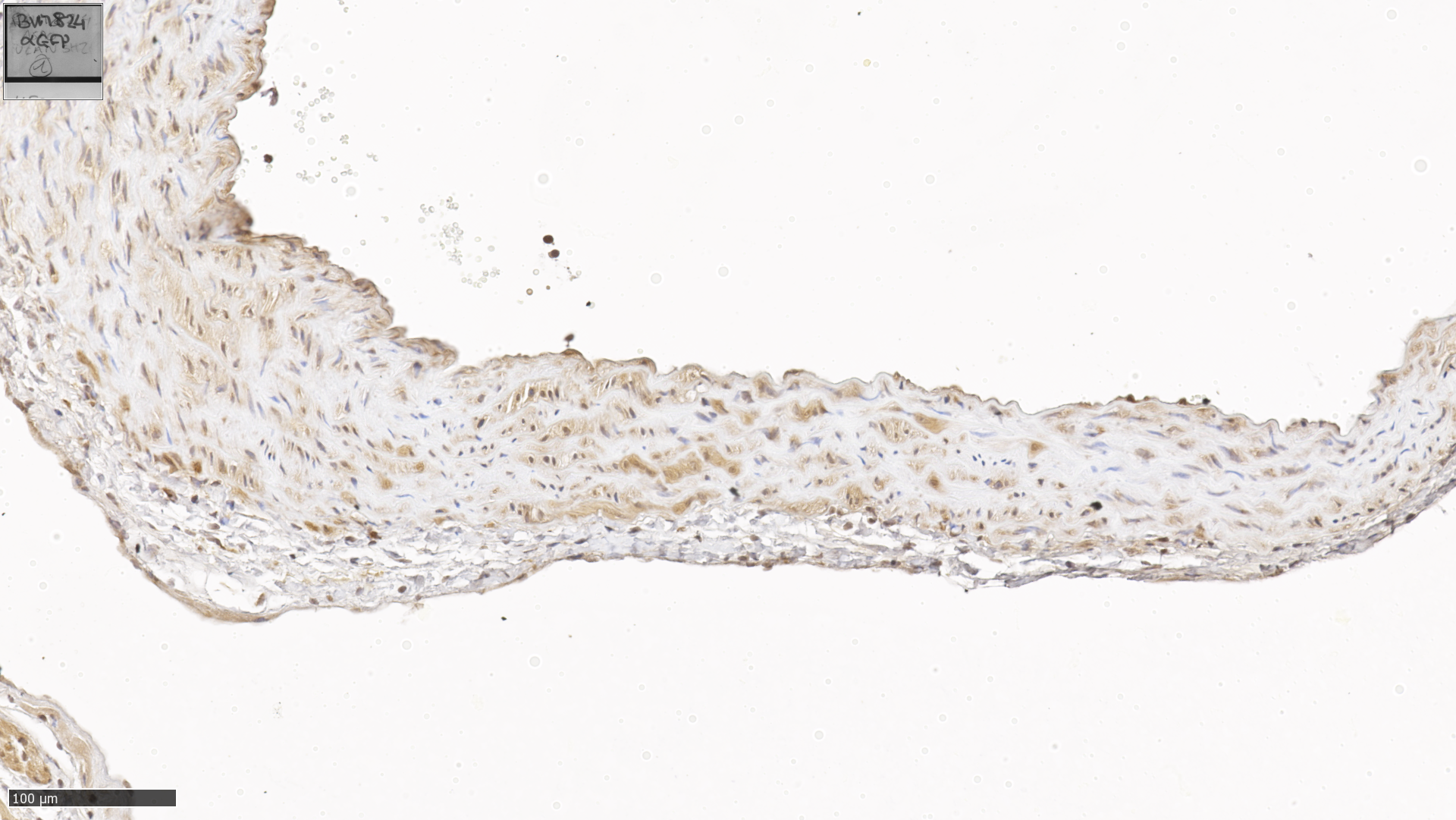

Supplement: Supplementary file 8 — Source Data Fig. 6 [file 44321_2023_9_MOESM8_ESM.zip › Figure 6/6C/ihq-gfp-asao-wt-scr.tif]

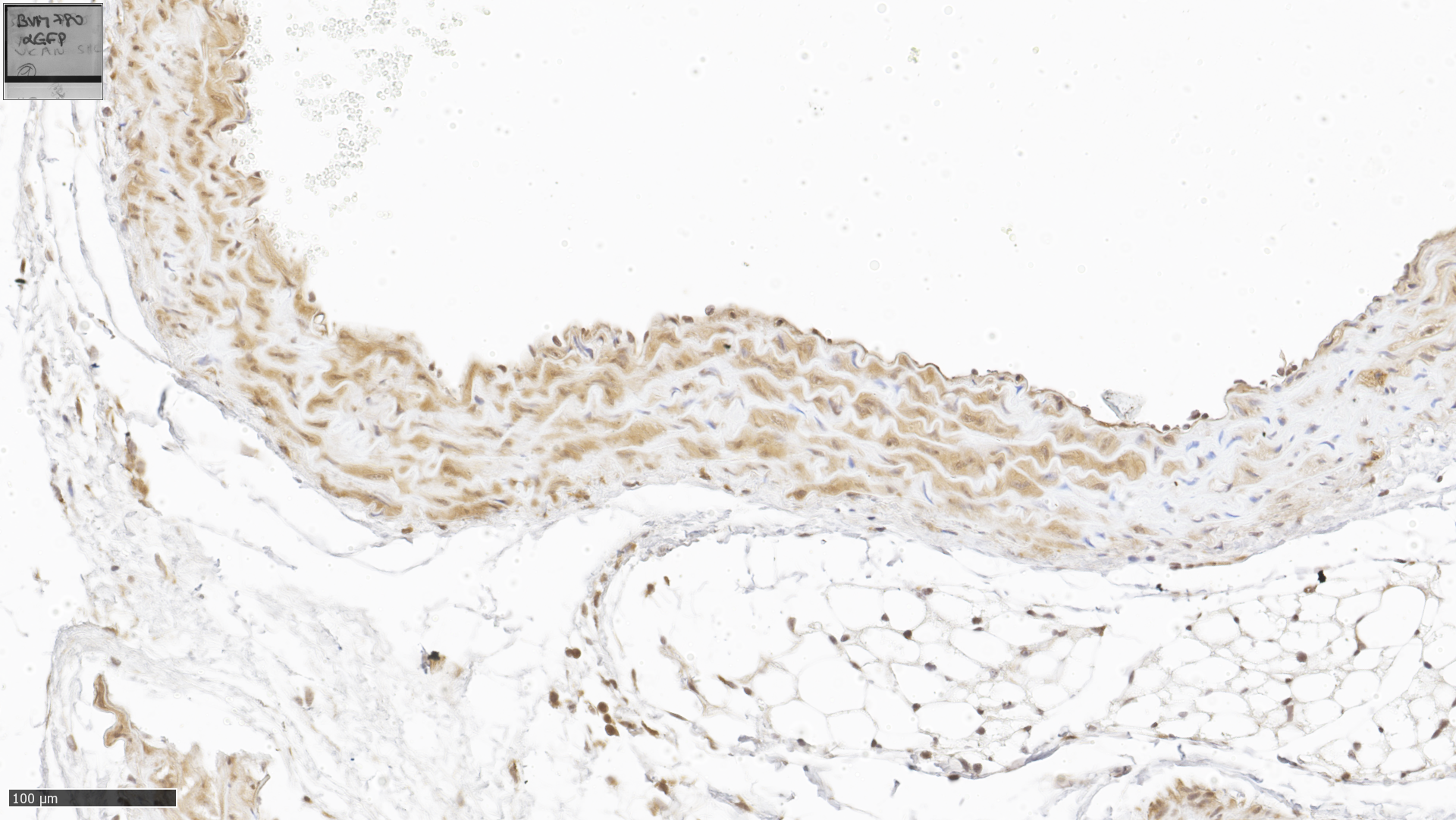

Supplement: Supplementary file 8 — Source Data Fig. 6 [file 44321_2023_9_MOESM8_ESM.zip › Figure 6/6C/ihq-gfp-asao-wt-vcan.tif]

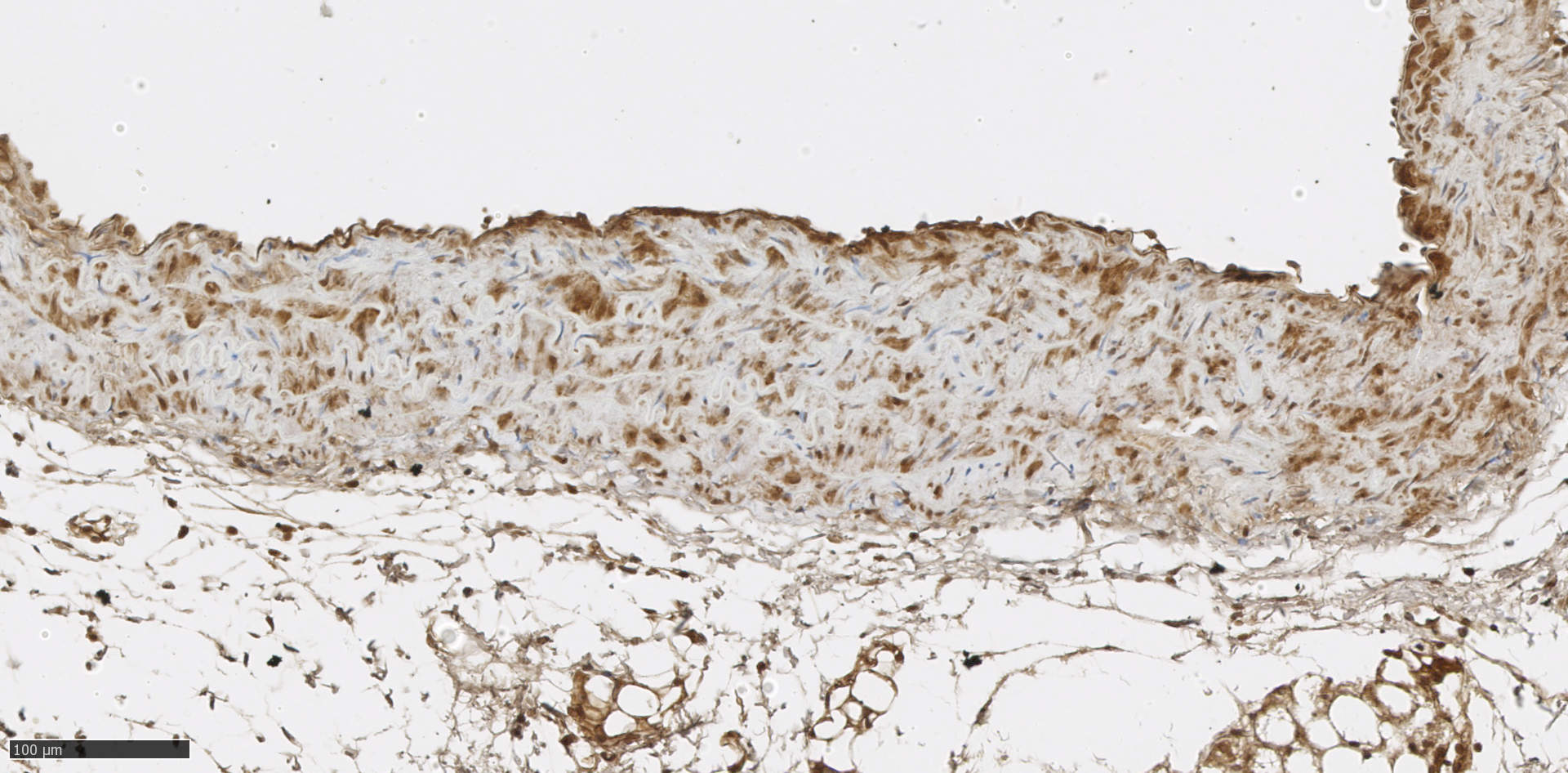

Supplement: Supplementary file 8 — Source Data Fig. 6 [file 44321_2023_9_MOESM8_ESM.zip › Figure 6/6D/ihq-vcan-mfs-scr.tif]

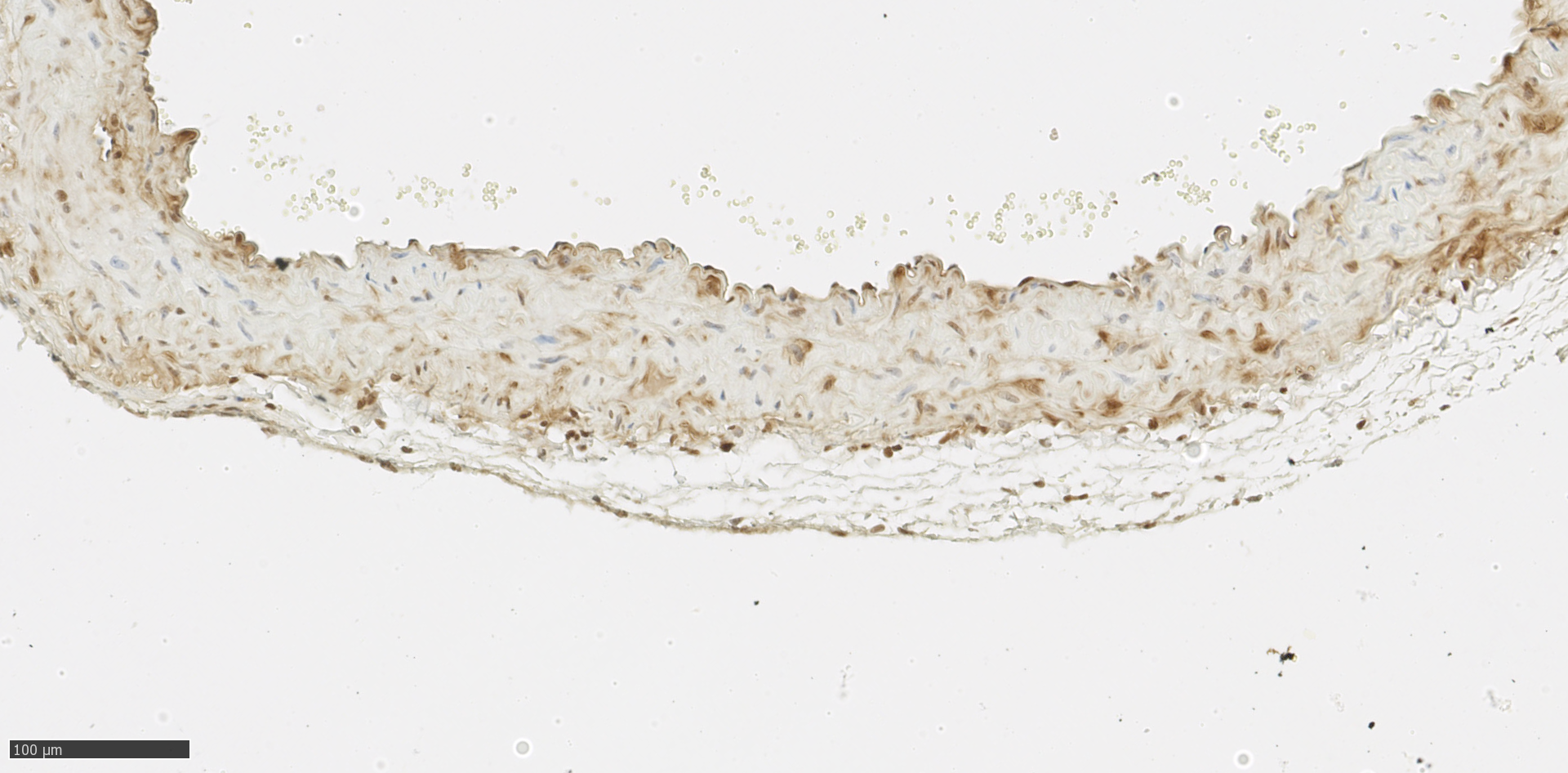

Supplement: Supplementary file 8 — Source Data Fig. 6 [file 44321_2023_9_MOESM8_ESM.zip › Figure 6/6D/ihq-vcan-mfs-vcan.tif]

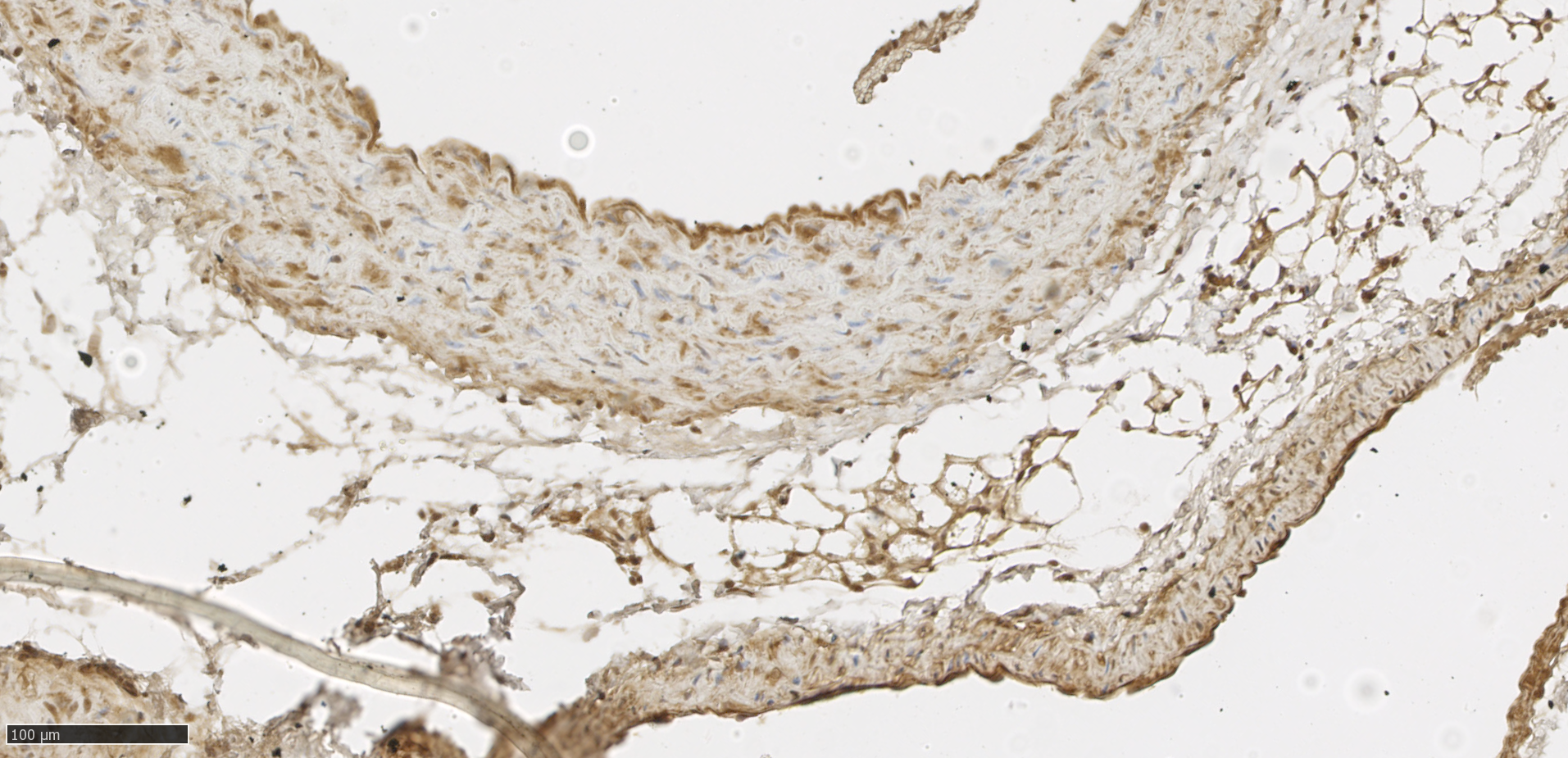

Supplement: Supplementary file 8 — Source Data Fig. 6 [file 44321_2023_9_MOESM8_ESM.zip › Figure 6/6D/ihq-vcan-wt-scr.tif]

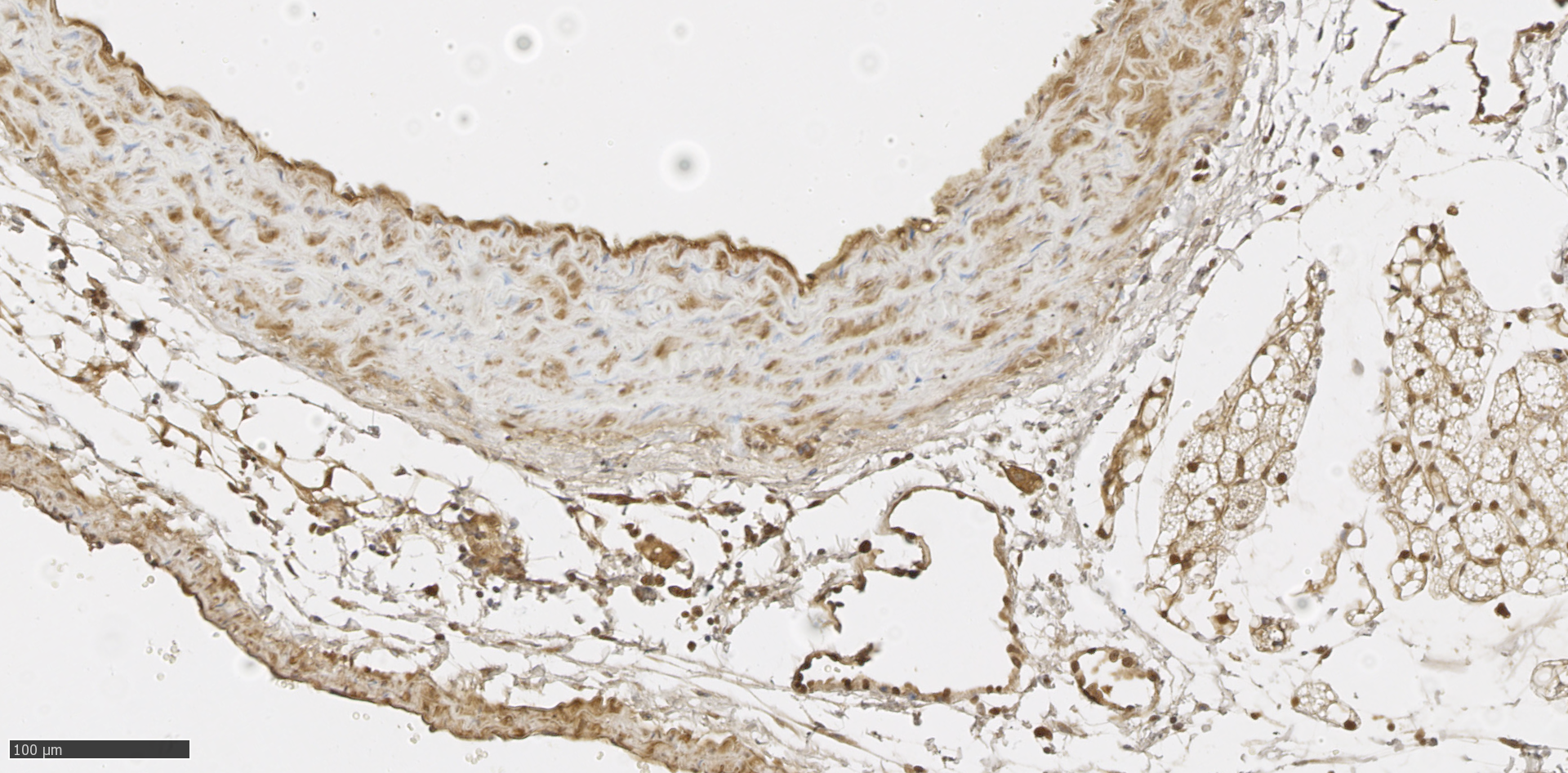

Supplement: Supplementary file 8 — Source Data Fig. 6 [file 44321_2023_9_MOESM8_ESM.zip › Figure 6/6D/ihq-vcan-wt-vcan.tif]

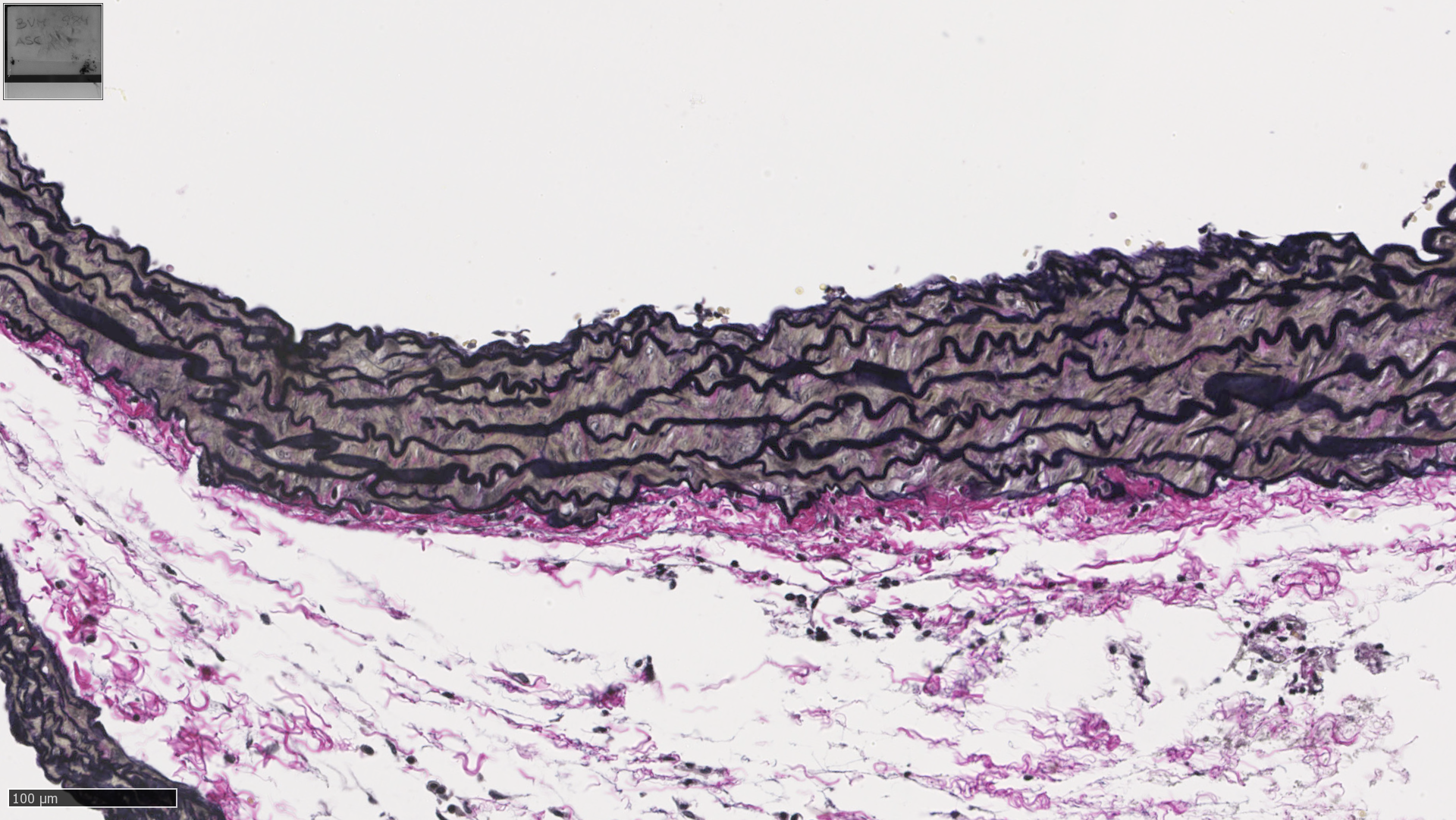

Supplement: Supplementary file 9 — Source Data Fig. 7 [file 44321_2023_9_MOESM9_ESM.zip › Figure 7/7A/evg-mfs-scr.tif]

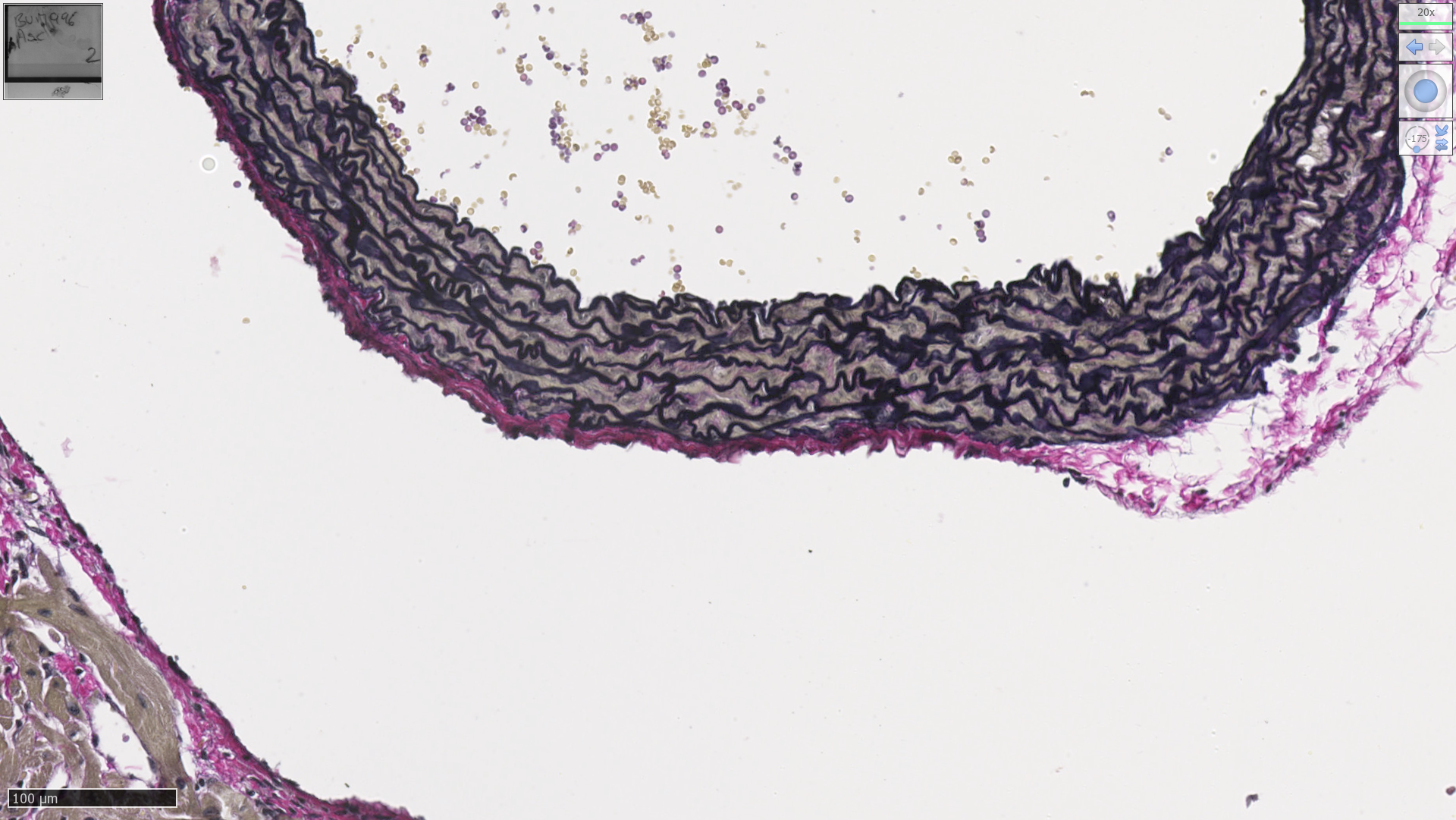

Supplement: Supplementary file 9 — Source Data Fig. 7 [file 44321_2023_9_MOESM9_ESM.zip › Figure 7/7A/evg-mfs-vcan.tif]

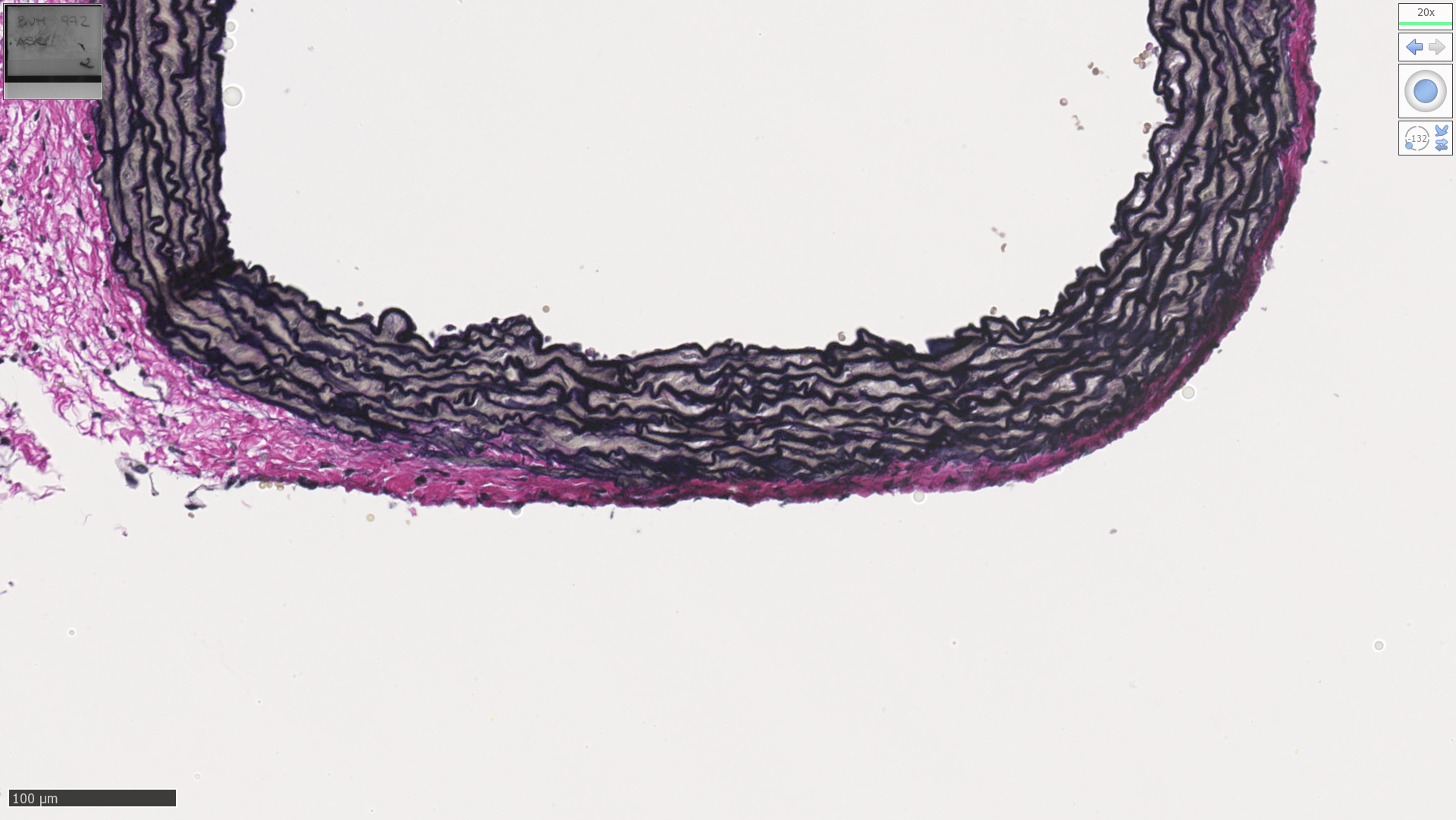

Supplement: Supplementary file 9 — Source Data Fig. 7 [file 44321_2023_9_MOESM9_ESM.zip › Figure 7/7A/evg-wt-scr.tif]

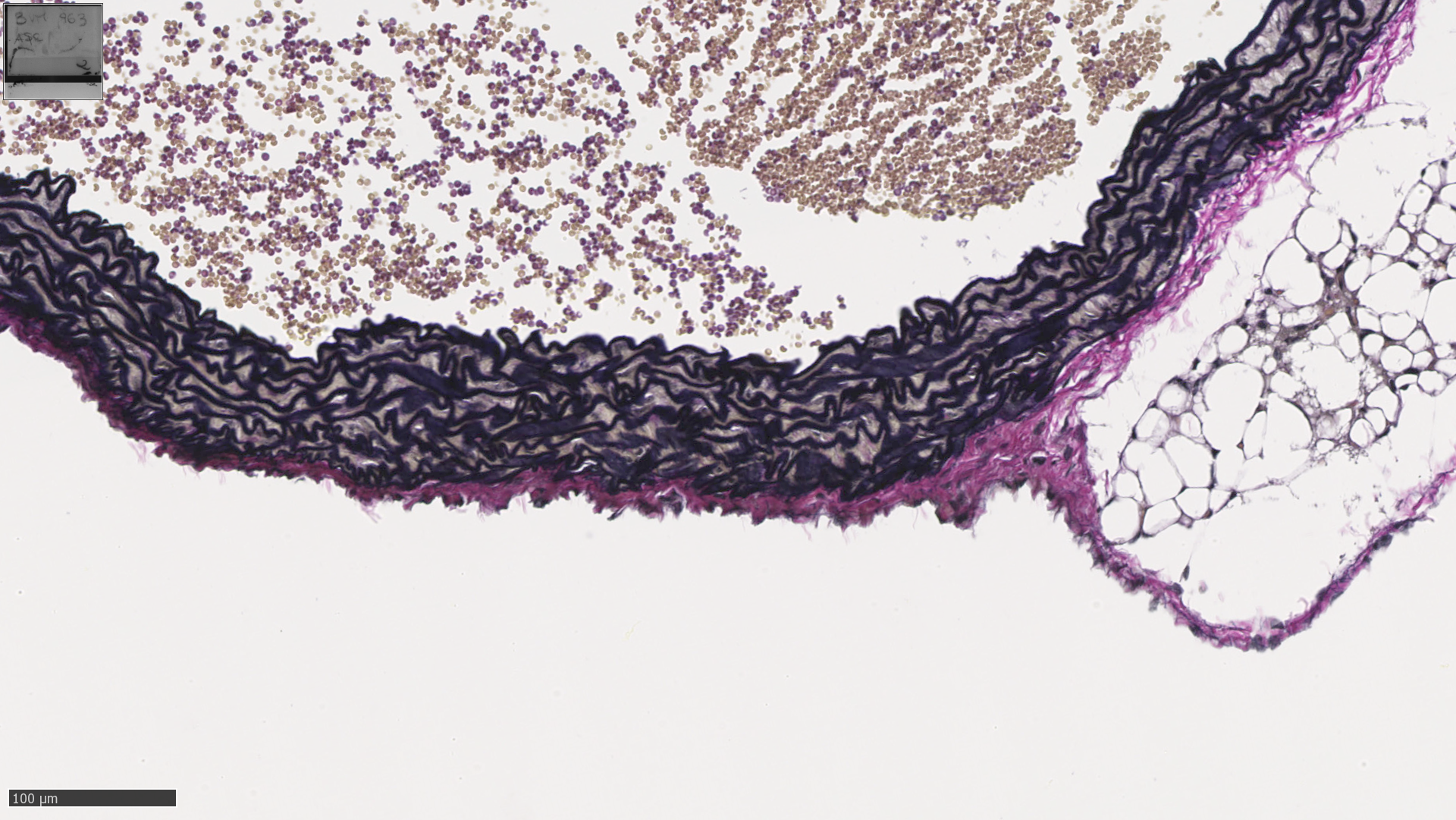

Supplement: Supplementary file 9 — Source Data Fig. 7 [file 44321_2023_9_MOESM9_ESM.zip › Figure 7/7A/evg-wt-vcan.tif]

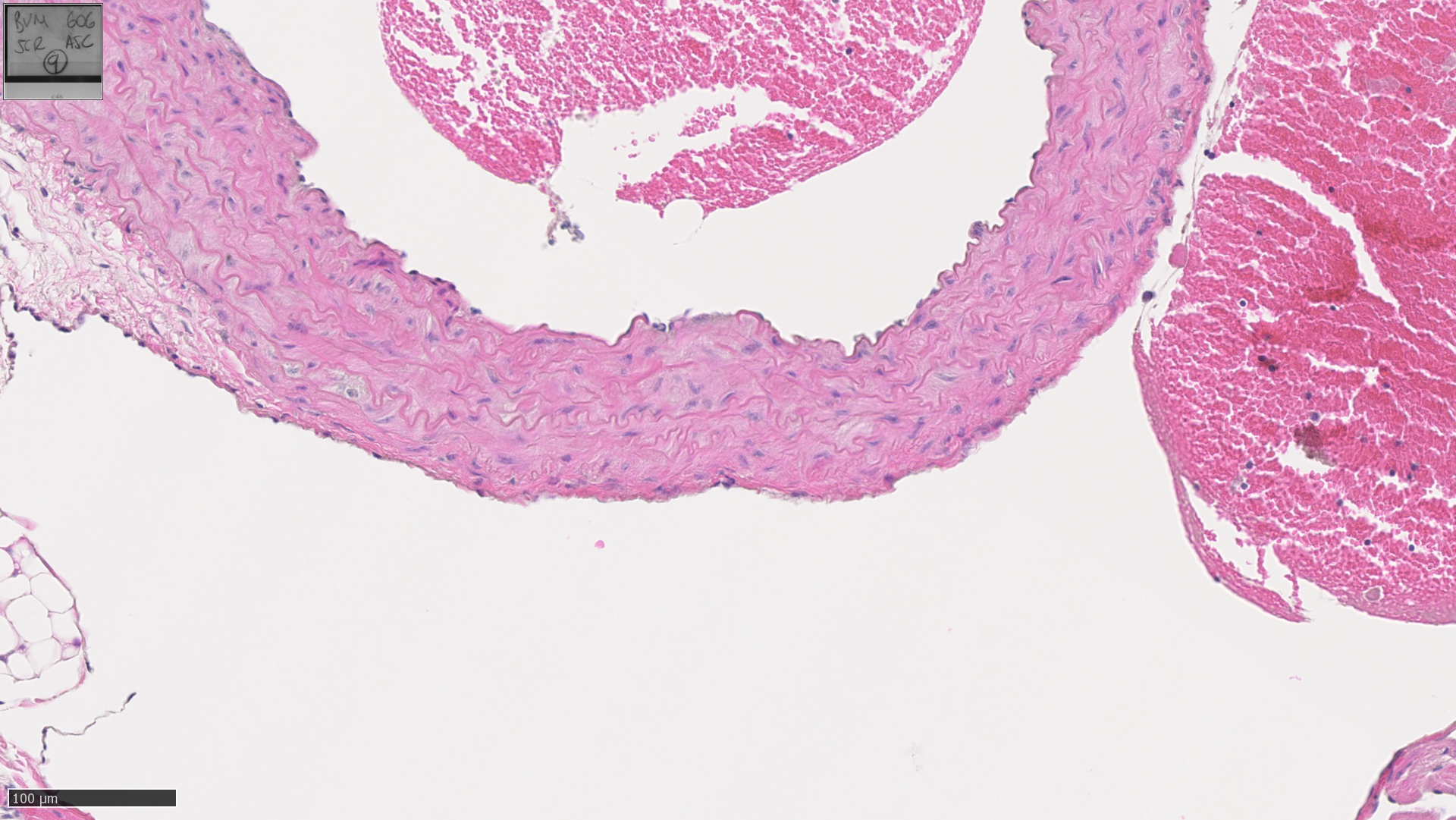

Supplement: Supplementary file 9 — Source Data Fig. 7 [file 44321_2023_9_MOESM9_ESM.zip › Figure 7/7A/h&e-mfs-scr.tif]

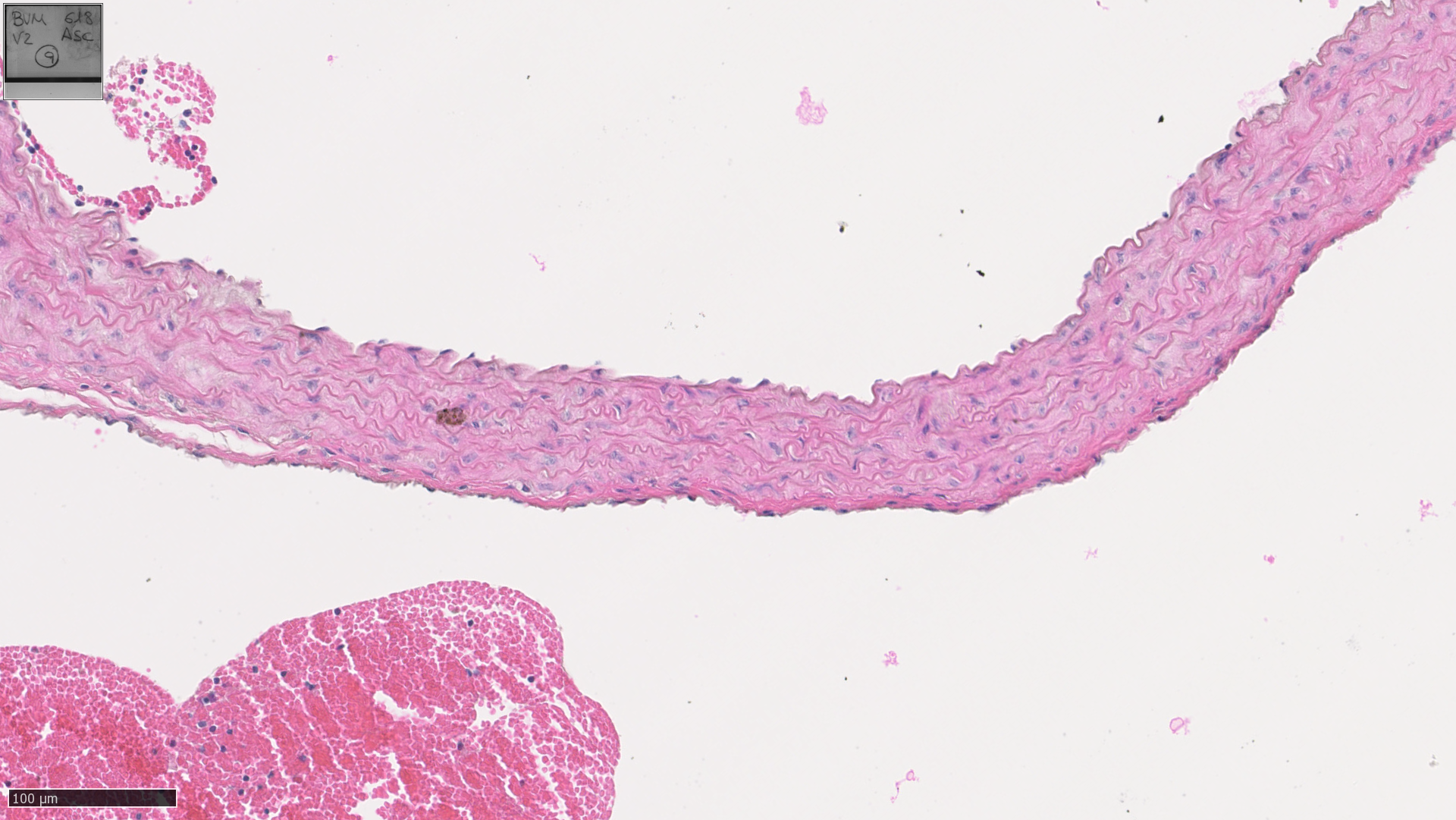

Supplement: Supplementary file 9 — Source Data Fig. 7 [file 44321_2023_9_MOESM9_ESM.zip › Figure 7/7A/h&e-mfs-vcan.tif]

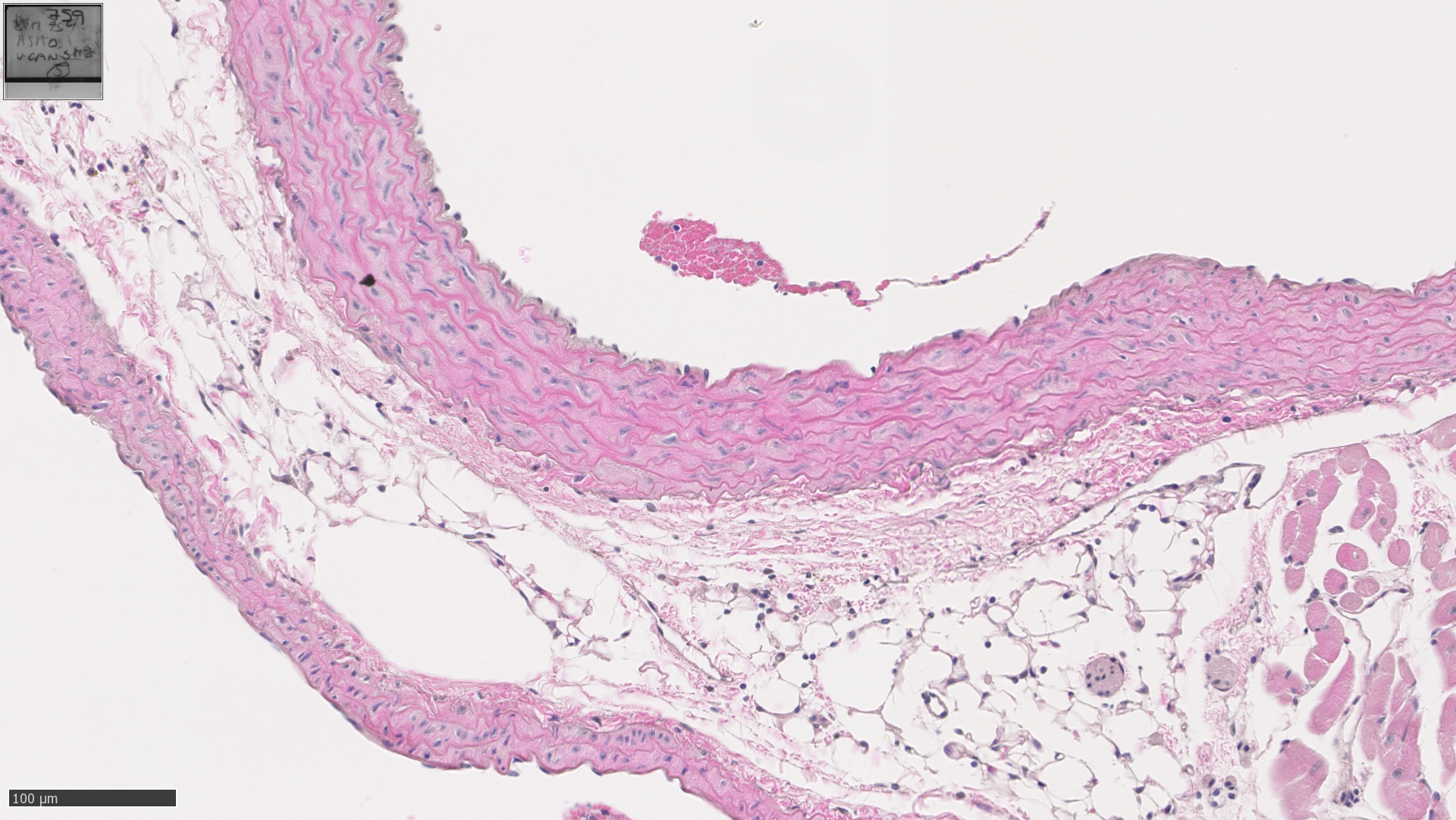

Supplement: Supplementary file 9 — Source Data Fig. 7 [file 44321_2023_9_MOESM9_ESM.zip › Figure 7/7A/h&e-wt-scr.tif]

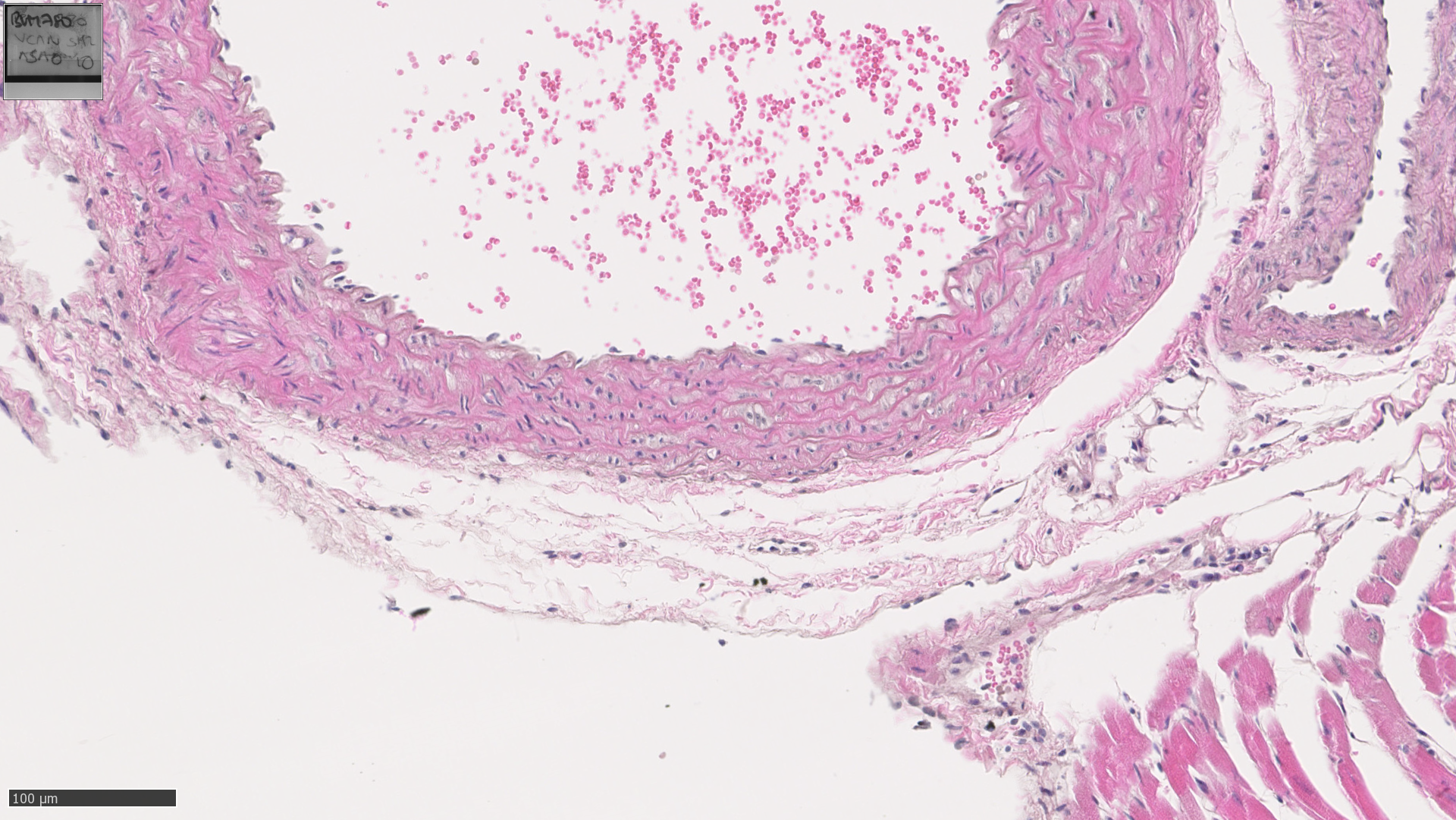

Supplement: Supplementary file 9 — Source Data Fig. 7 [file 44321_2023_9_MOESM9_ESM.zip › Figure 7/7A/h&e-wt-vcan.tif]

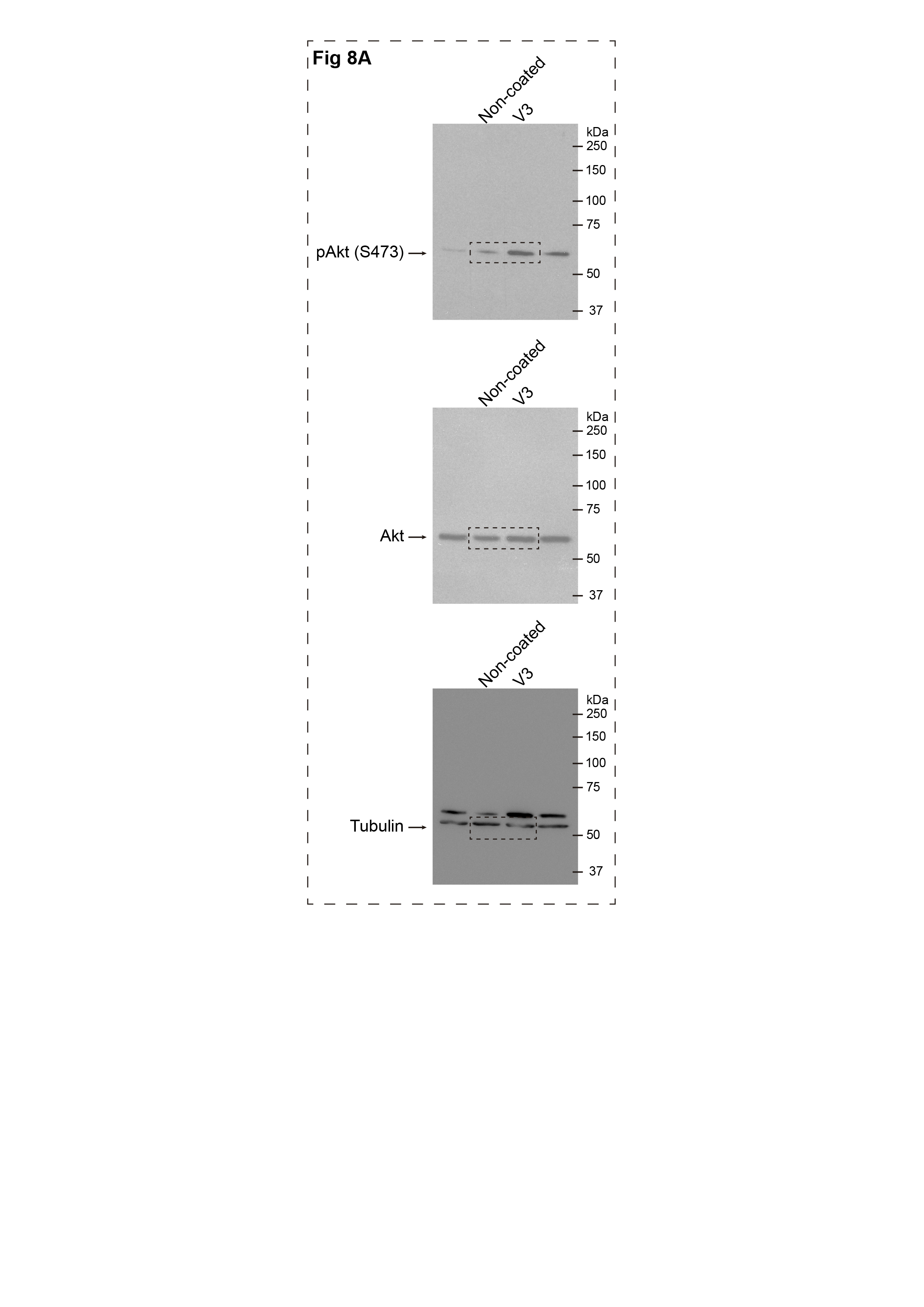

Supplement: Supplementary file 10 — Source Data Fig. 8 [file 44321_2023_9_MOESM10_ESM.zip › Figure 8/8A/cropped-blot.tif]

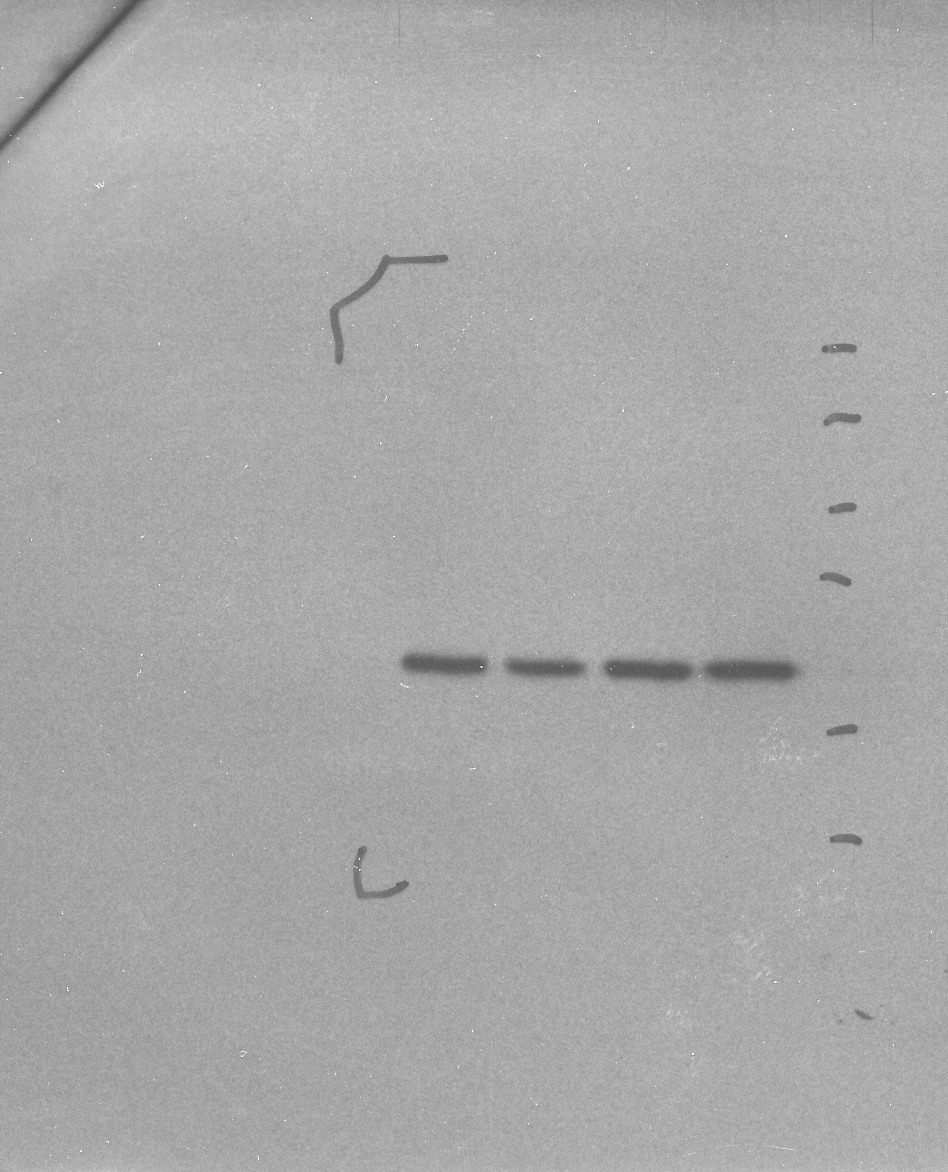

Supplement: Supplementary file 10 — Source Data Fig. 8 [file 44321_2023_9_MOESM10_ESM.zip › Figure 8/8A/wb-akt total.tif]

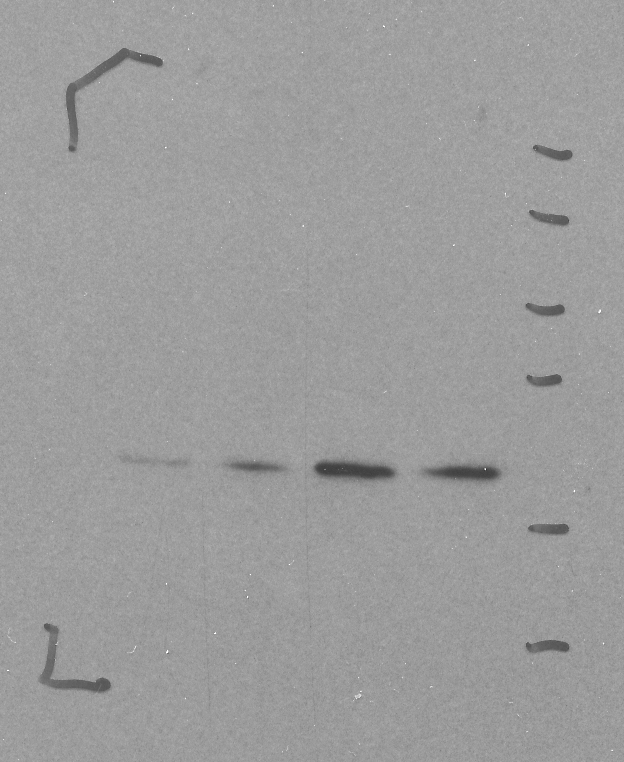

Supplement: Supplementary file 10 — Source Data Fig. 8 [file 44321_2023_9_MOESM10_ESM.zip › Figure 8/8A/wb-pakt.tif]

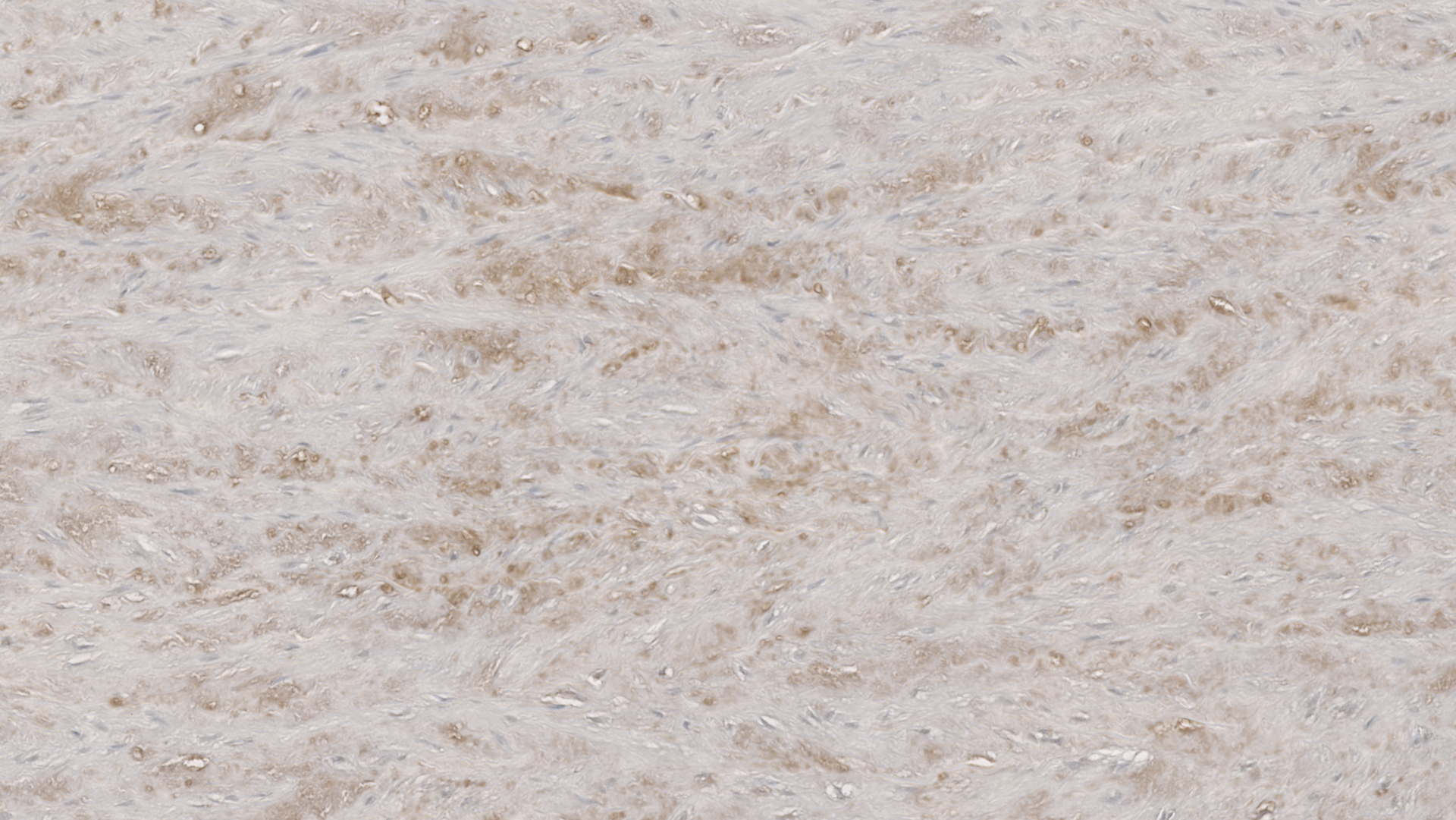

Supplement: Supplementary file 11 — Source Data EV Figures [file 44321_2023_9_MOESM11_ESM.zip › Figure EV2/ACAN-Ctrl-11_3.tif]

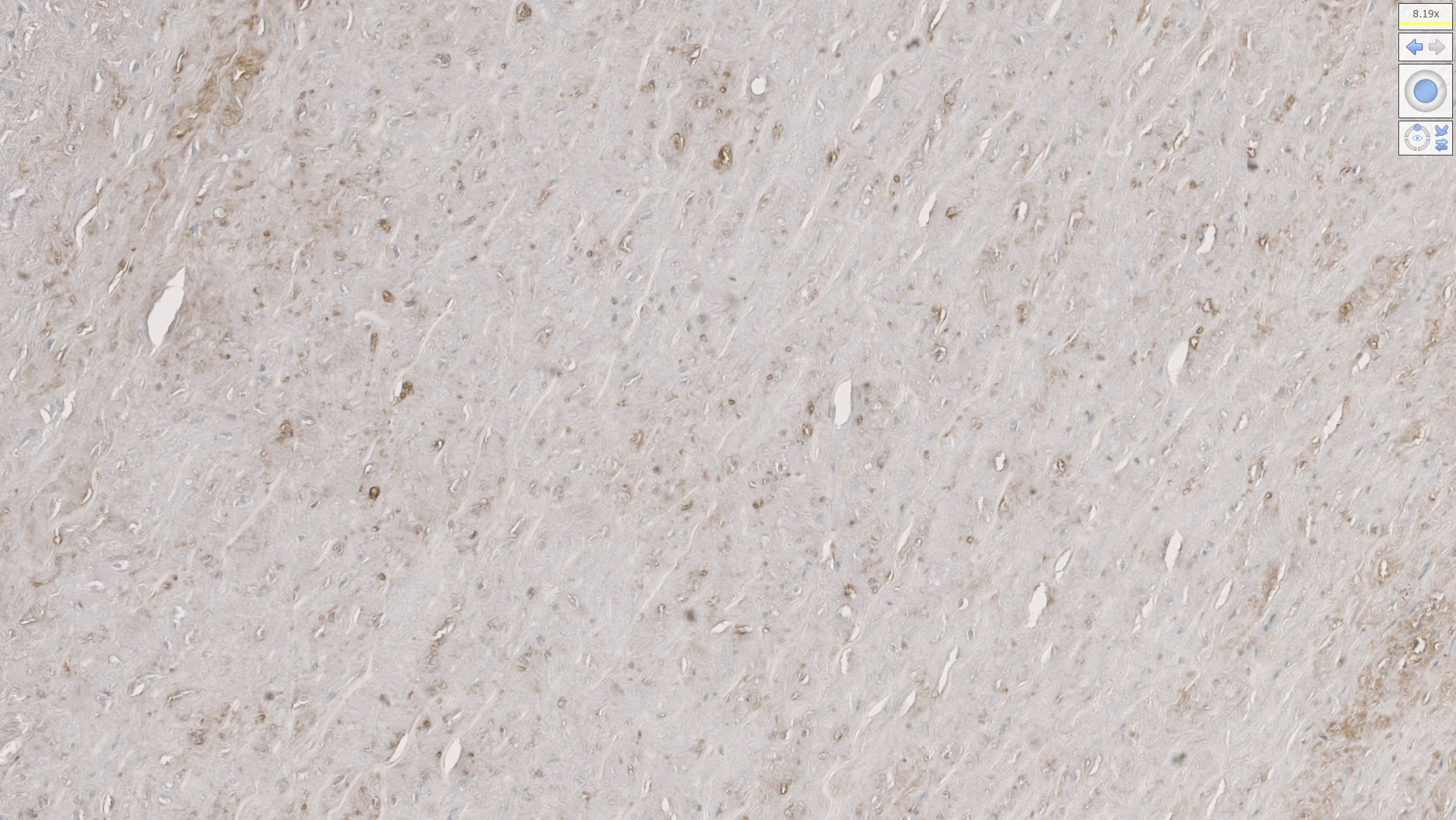

Supplement: Supplementary file 11 — Source Data EV Figures [file 44321_2023_9_MOESM11_ESM.zip › Figure EV2/ACAN-Ctrl-49_3.tif]

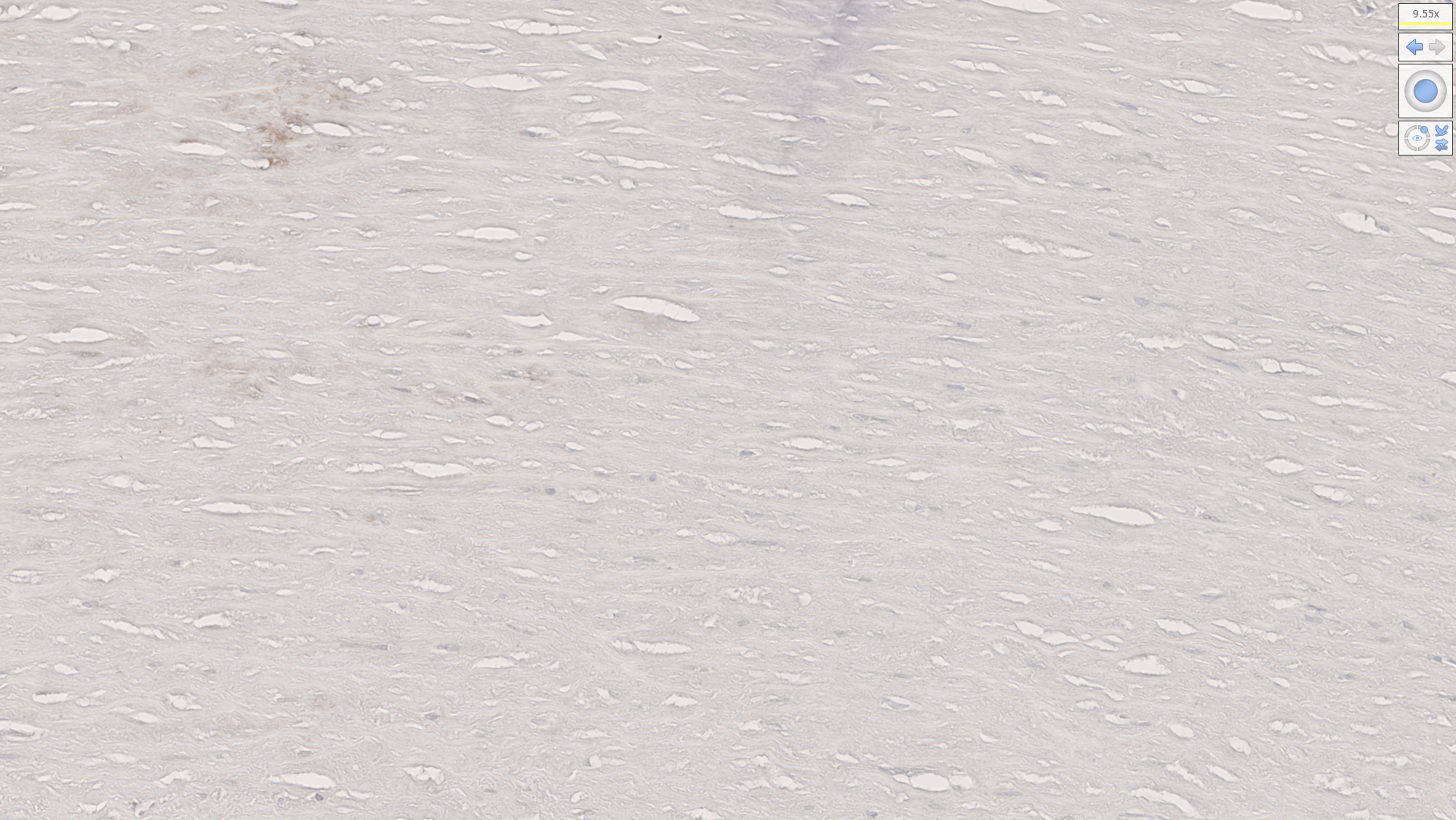

Supplement: Supplementary file 11 — Source Data EV Figures [file 44321_2023_9_MOESM11_ESM.zip › Figure EV2/ACAN-Ctrl-78_1.tif]

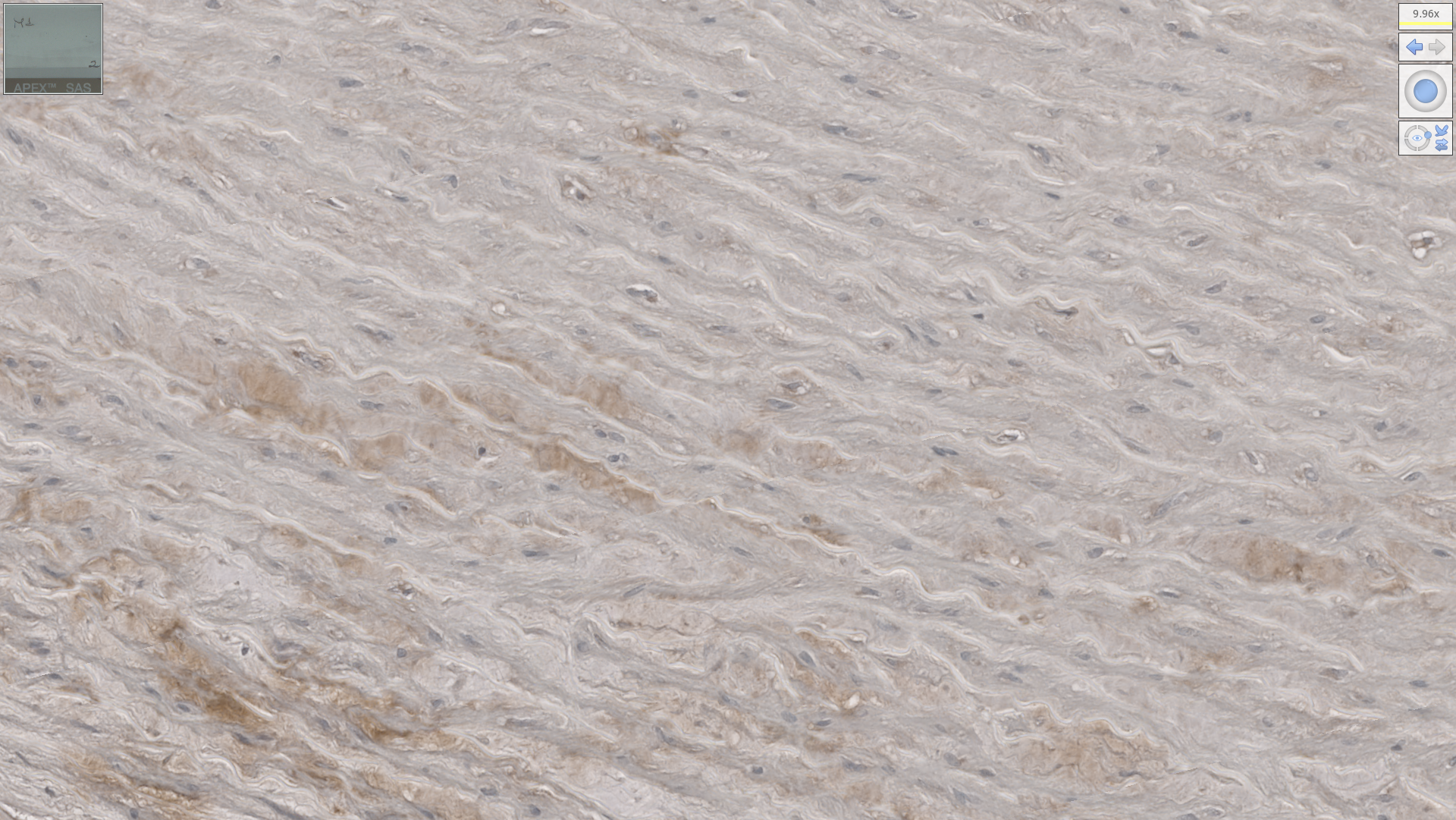

Supplement: Supplementary file 11 — Source Data EV Figures [file 44321_2023_9_MOESM11_ESM.zip › Figure EV2/ACAN-MFS-a1_2.tif]

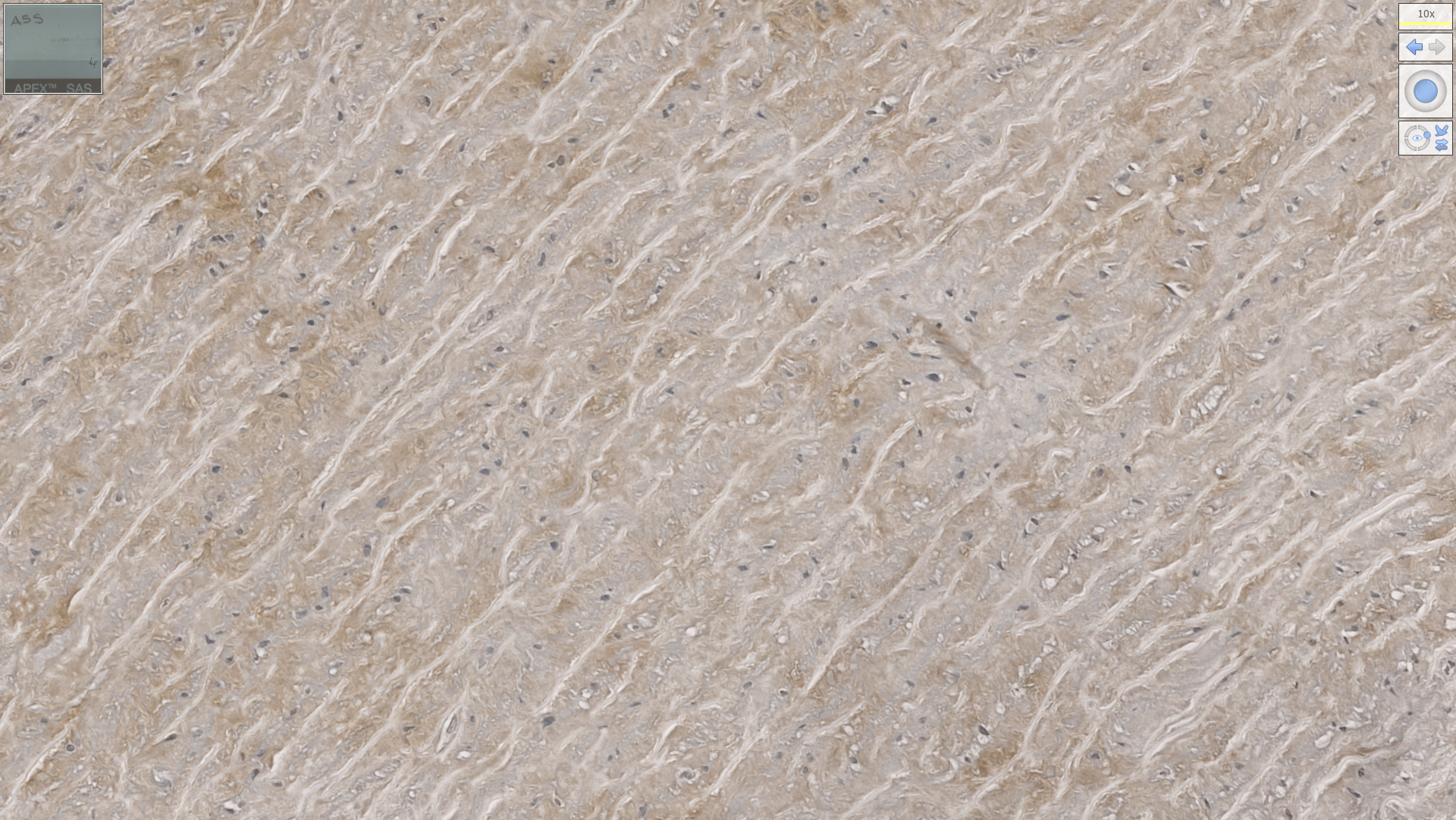

Supplement: Supplementary file 11 — Source Data EV Figures [file 44321_2023_9_MOESM11_ESM.zip › Figure EV2/ACAN-MFS-a55_2.tif]

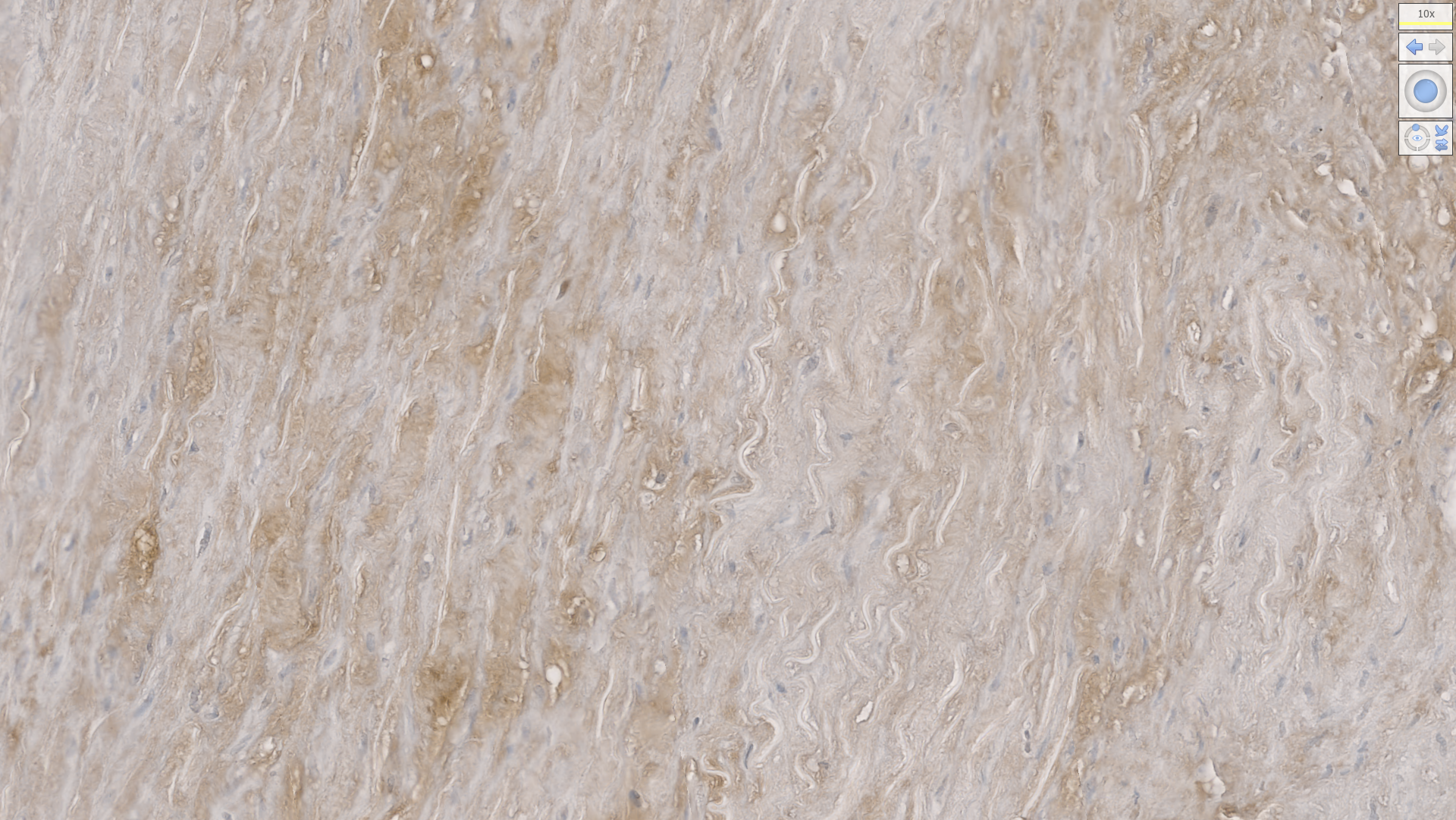

Supplement: Supplementary file 11 — Source Data EV Figures [file 44321_2023_9_MOESM11_ESM.zip › Figure EV2/ACAN-MFS-ao26_2.tif]

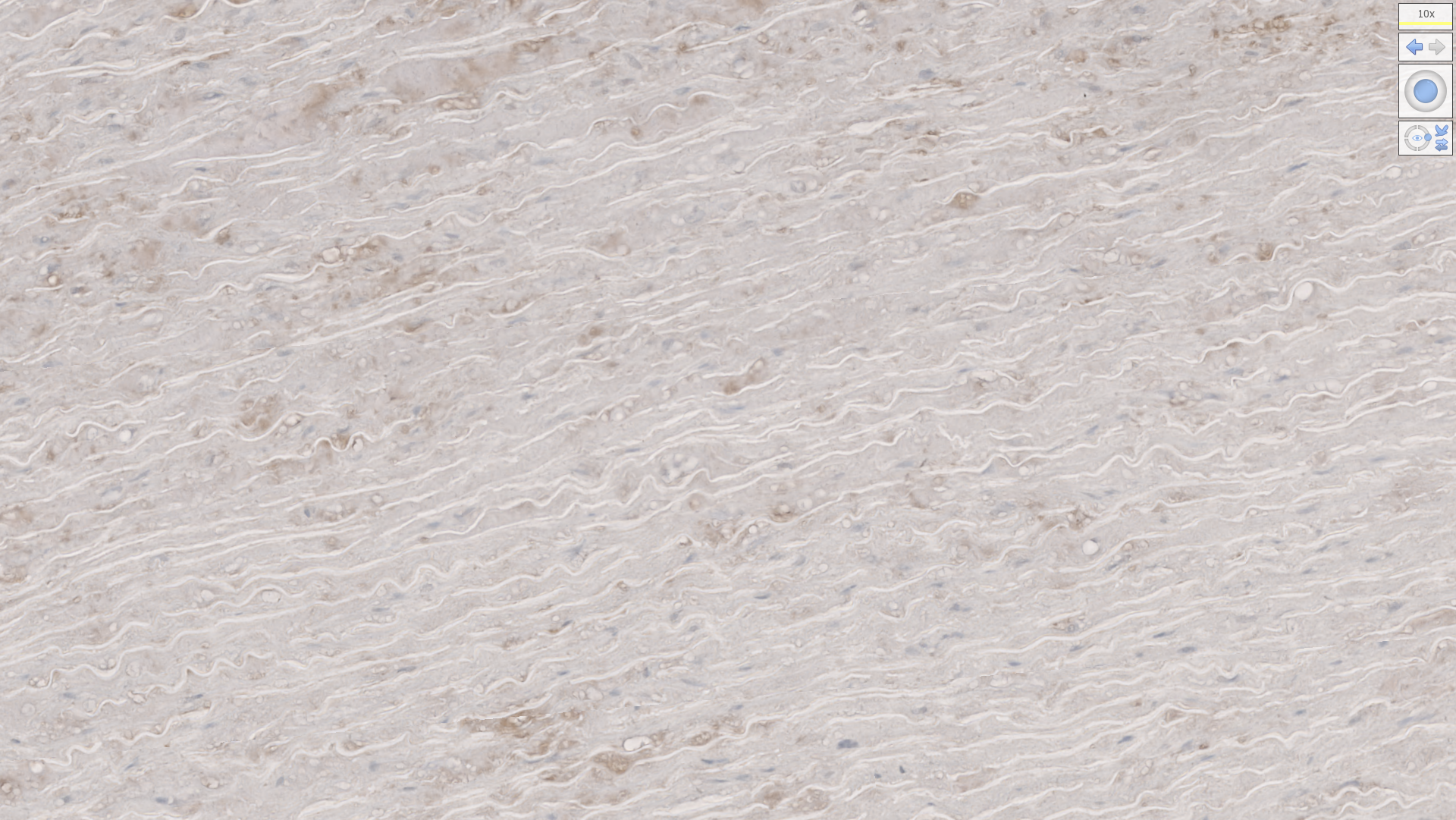

Supplement: Supplementary file 11 — Source Data EV Figures [file 44321_2023_9_MOESM11_ESM.zip › Figure EV2/ACAN-MFS-ao41_2.tif]

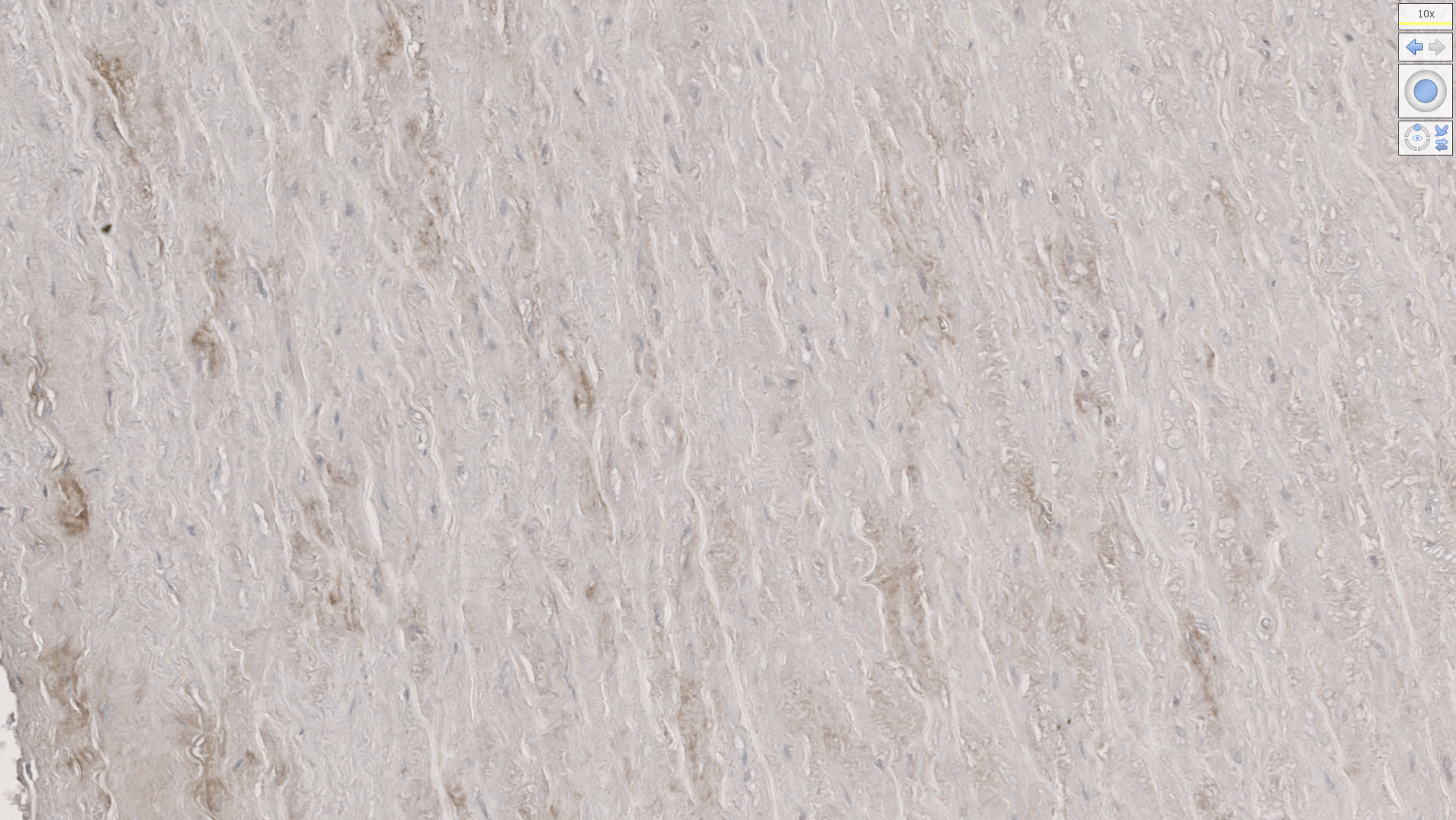

Supplement: Supplementary file 11 — Source Data EV Figures [file 44321_2023_9_MOESM11_ESM.zip › Figure EV2/ACAN-MFS-ao7_3.tif]

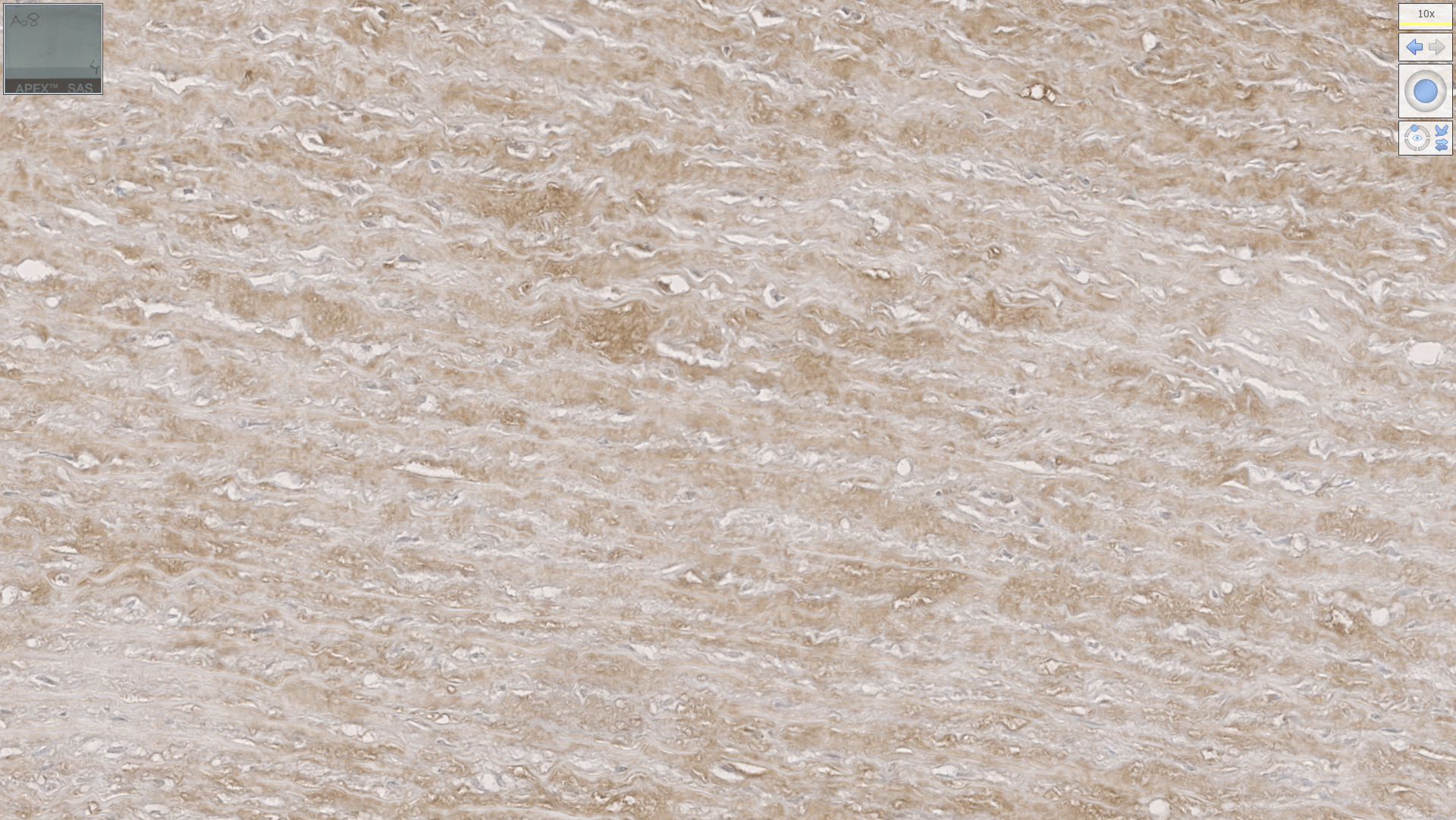

Supplement: Supplementary file 11 — Source Data EV Figures [file 44321_2023_9_MOESM11_ESM.zip › Figure EV2/ACAN-MFS-ao8_2.tif]

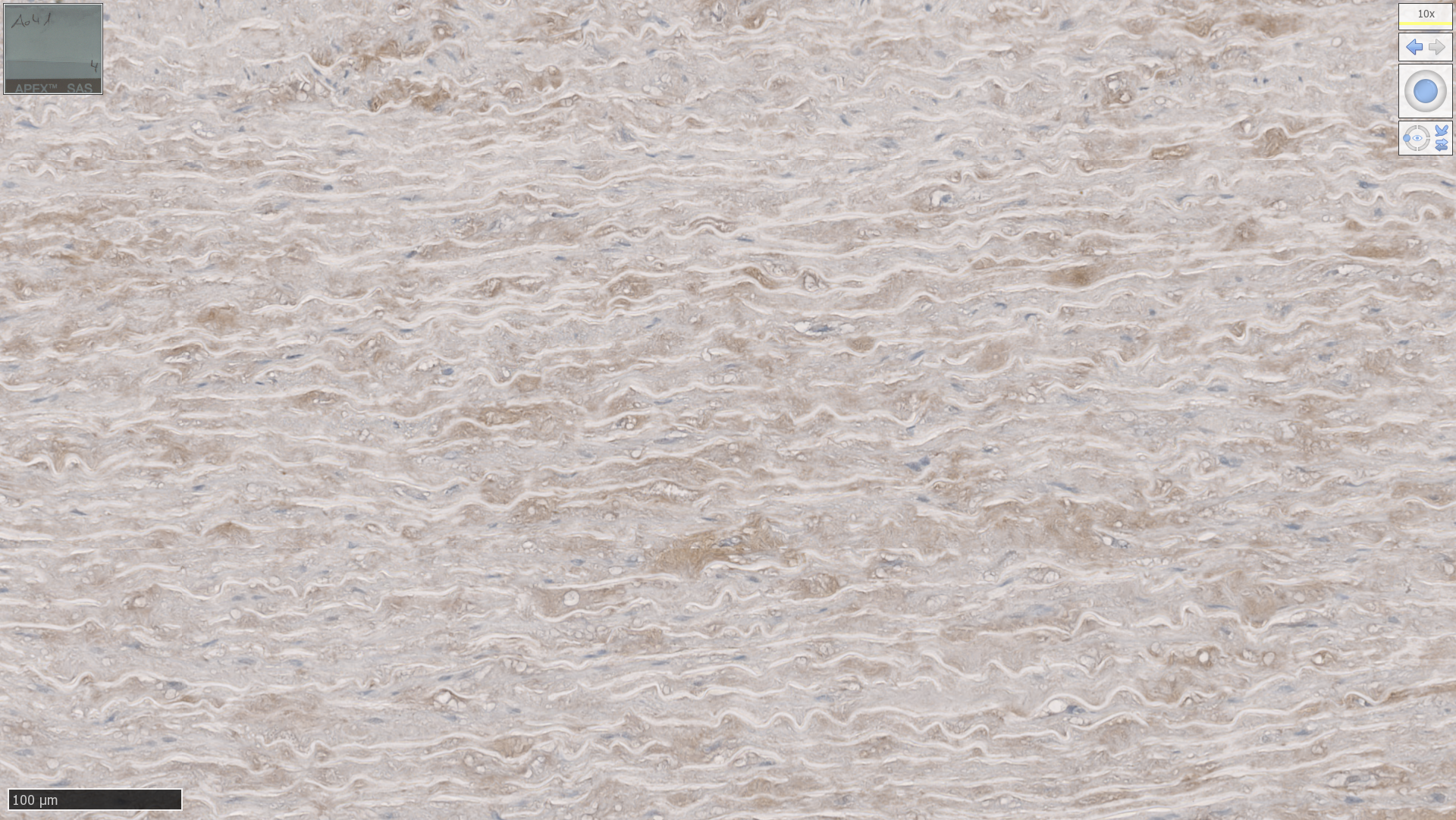

Supplement: Supplementary file 11 — Source Data EV Figures [file 44321_2023_9_MOESM11_ESM.zip › Figure EV2/scale bar.tif]

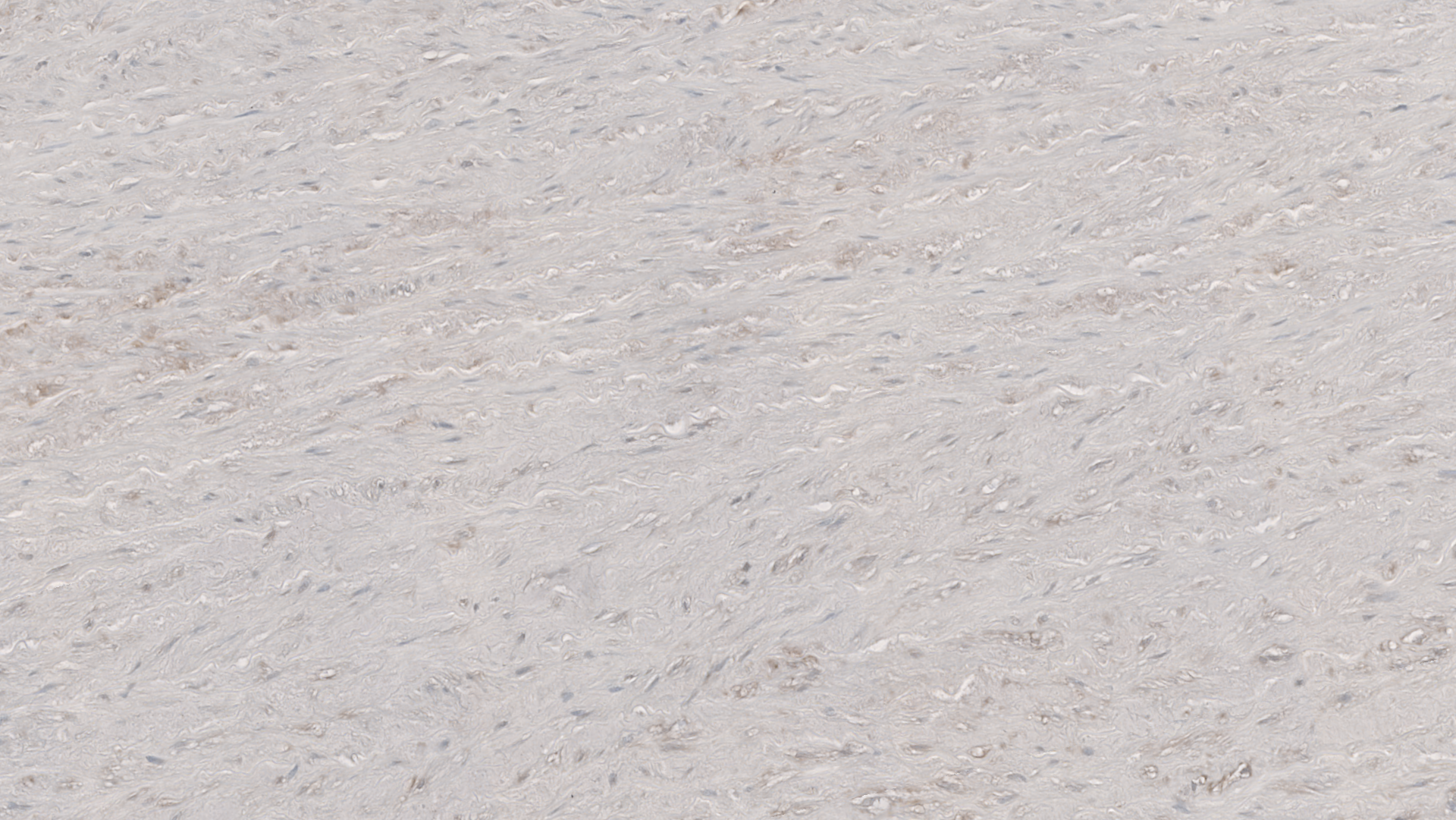

Supplement: Supplementary file 11 — Source Data EV Figures [file 44321_2023_9_MOESM11_ESM.zip › Figure EV2/VCAN-Ctrl-11_1.tif]

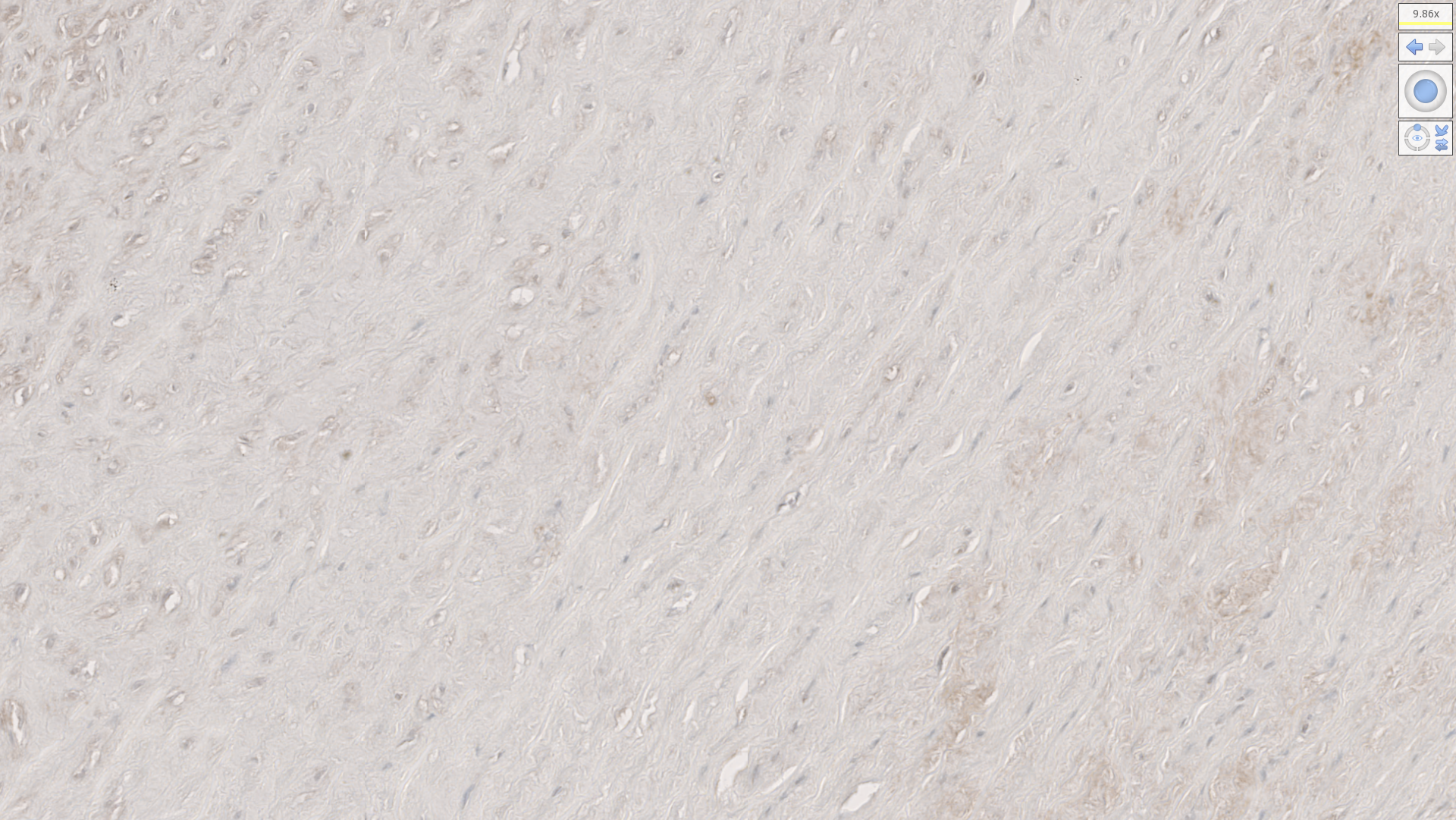

Supplement: Supplementary file 11 — Source Data EV Figures [file 44321_2023_9_MOESM11_ESM.zip › Figure EV2/VCAN-Ctrl-49_3.tif]

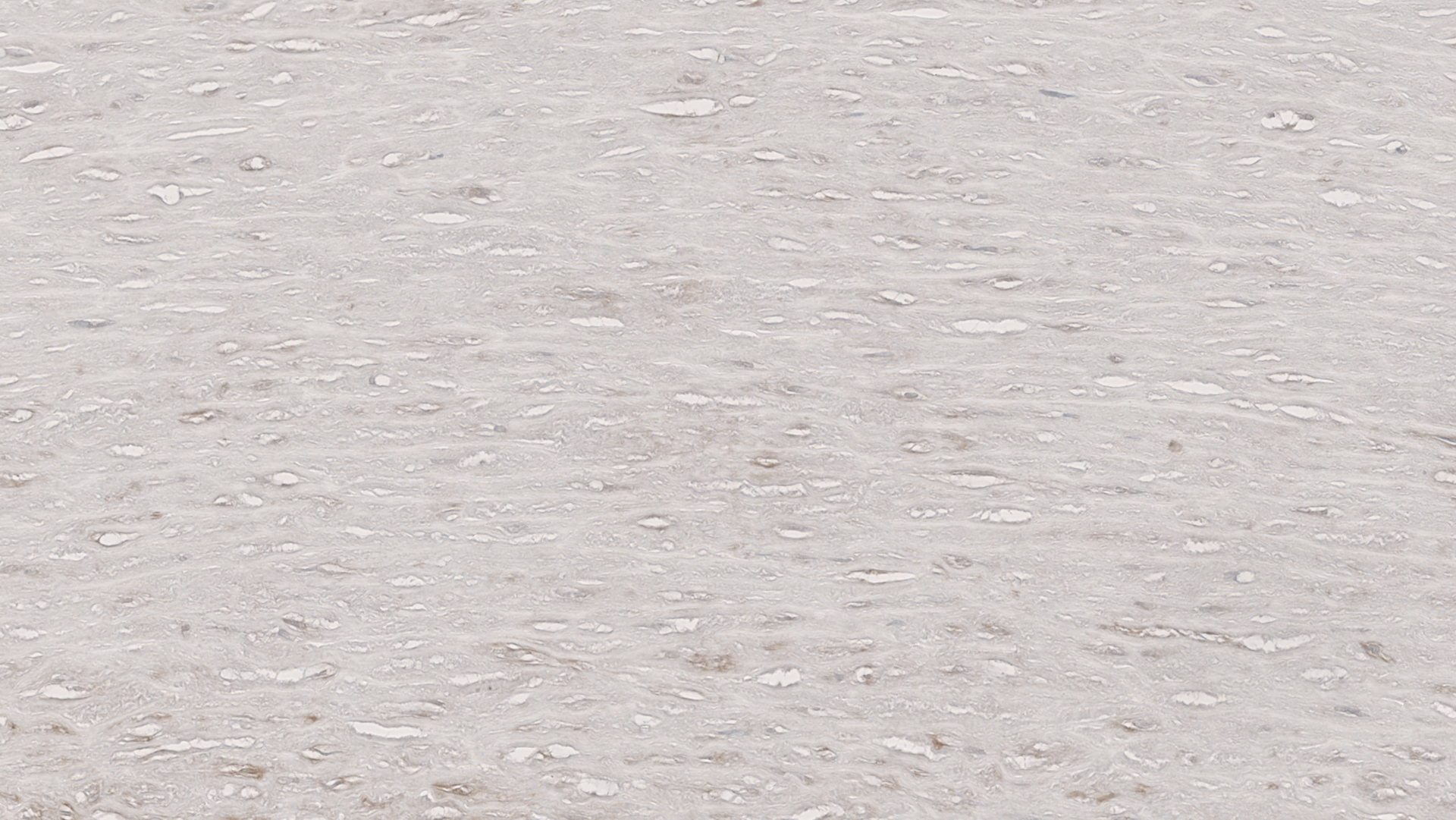

Supplement: Supplementary file 11 — Source Data EV Figures [file 44321_2023_9_MOESM11_ESM.zip › Figure EV2/VCAN-Ctrl-78_3.tif]

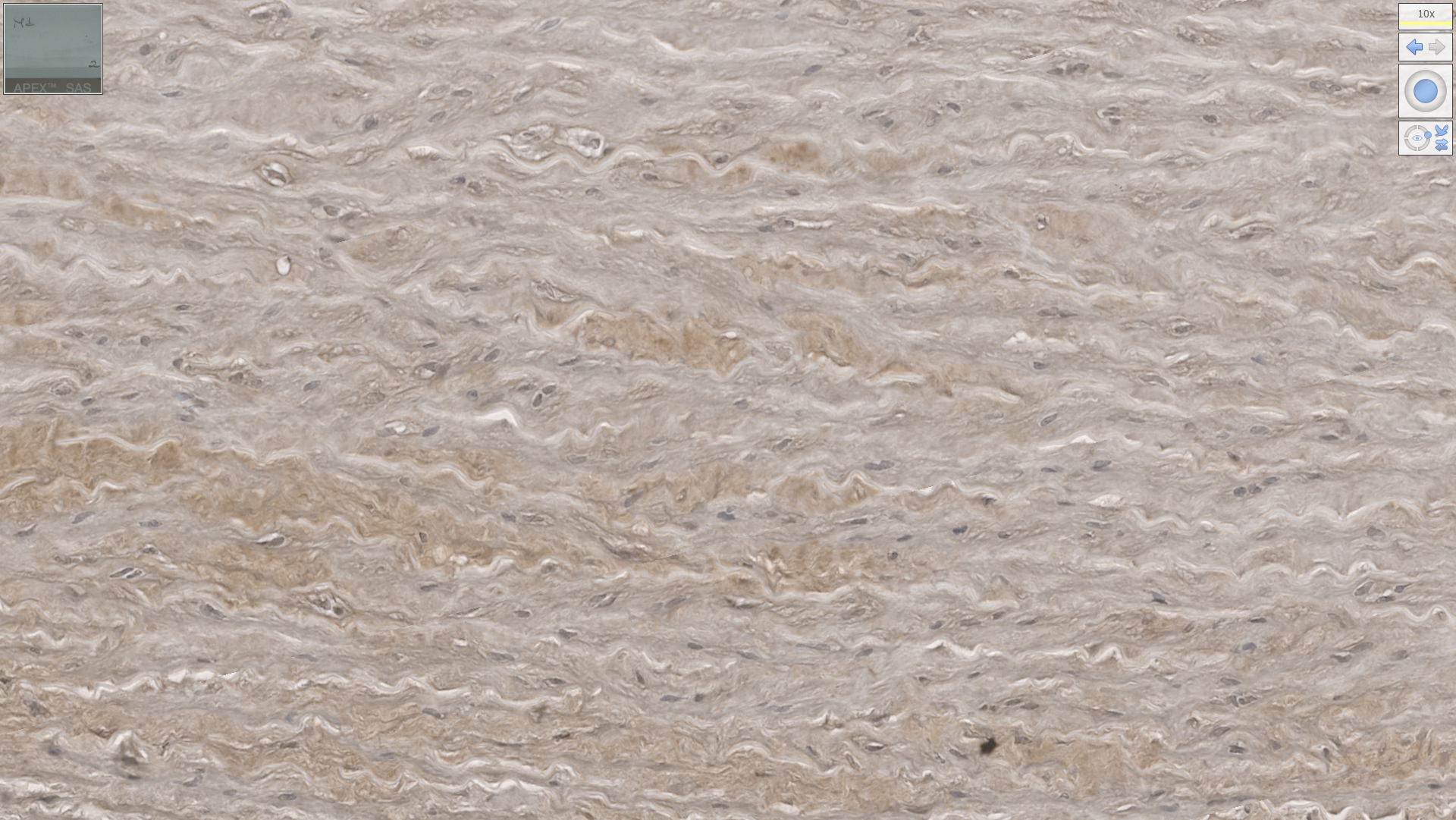

Supplement: Supplementary file 11 — Source Data EV Figures [file 44321_2023_9_MOESM11_ESM.zip › Figure EV2/VCAN-MFS-a1_2.tif]

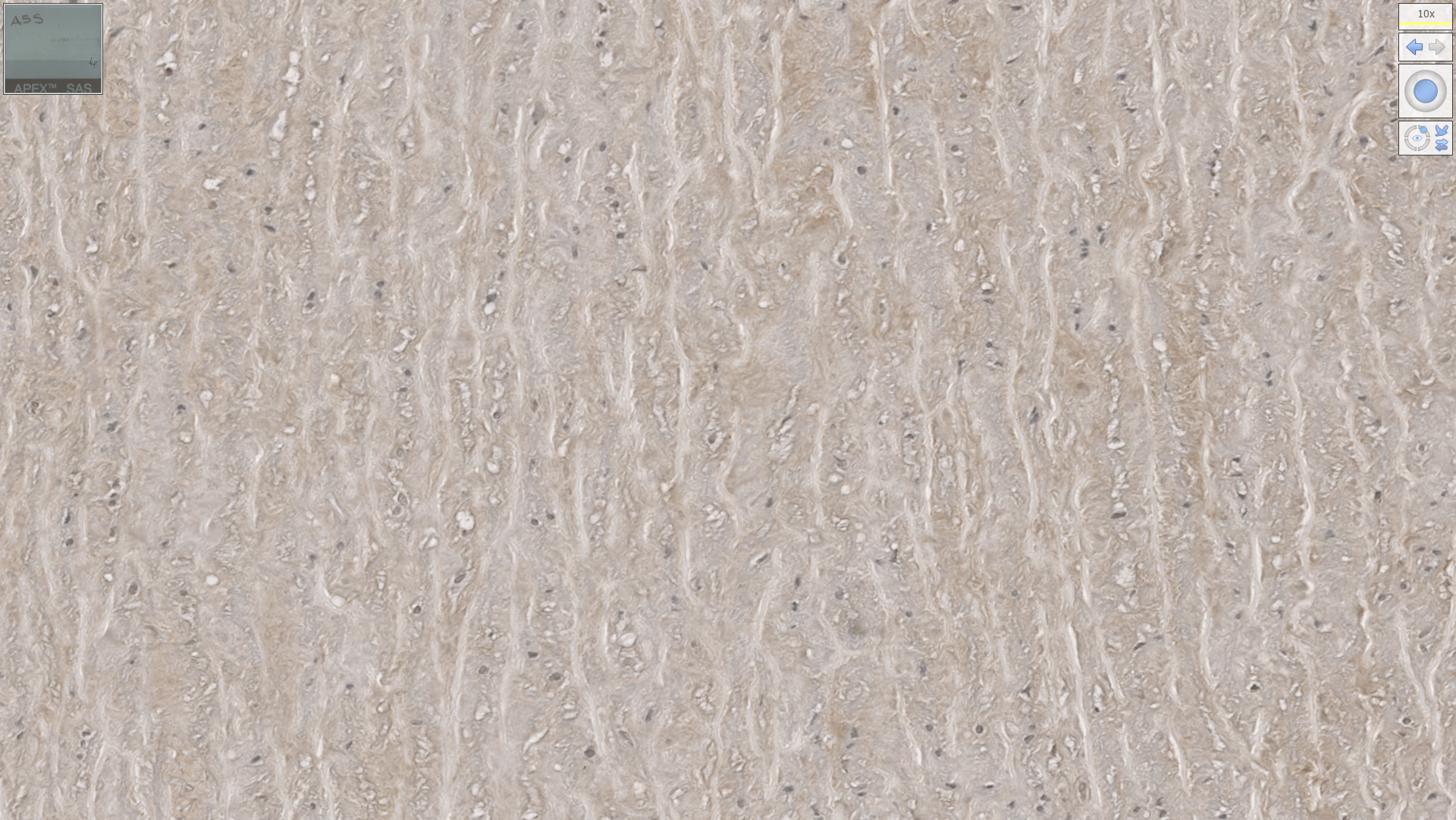

Supplement: Supplementary file 11 — Source Data EV Figures [file 44321_2023_9_MOESM11_ESM.zip › Figure EV2/VCAN-MFS-a55_3.tif]

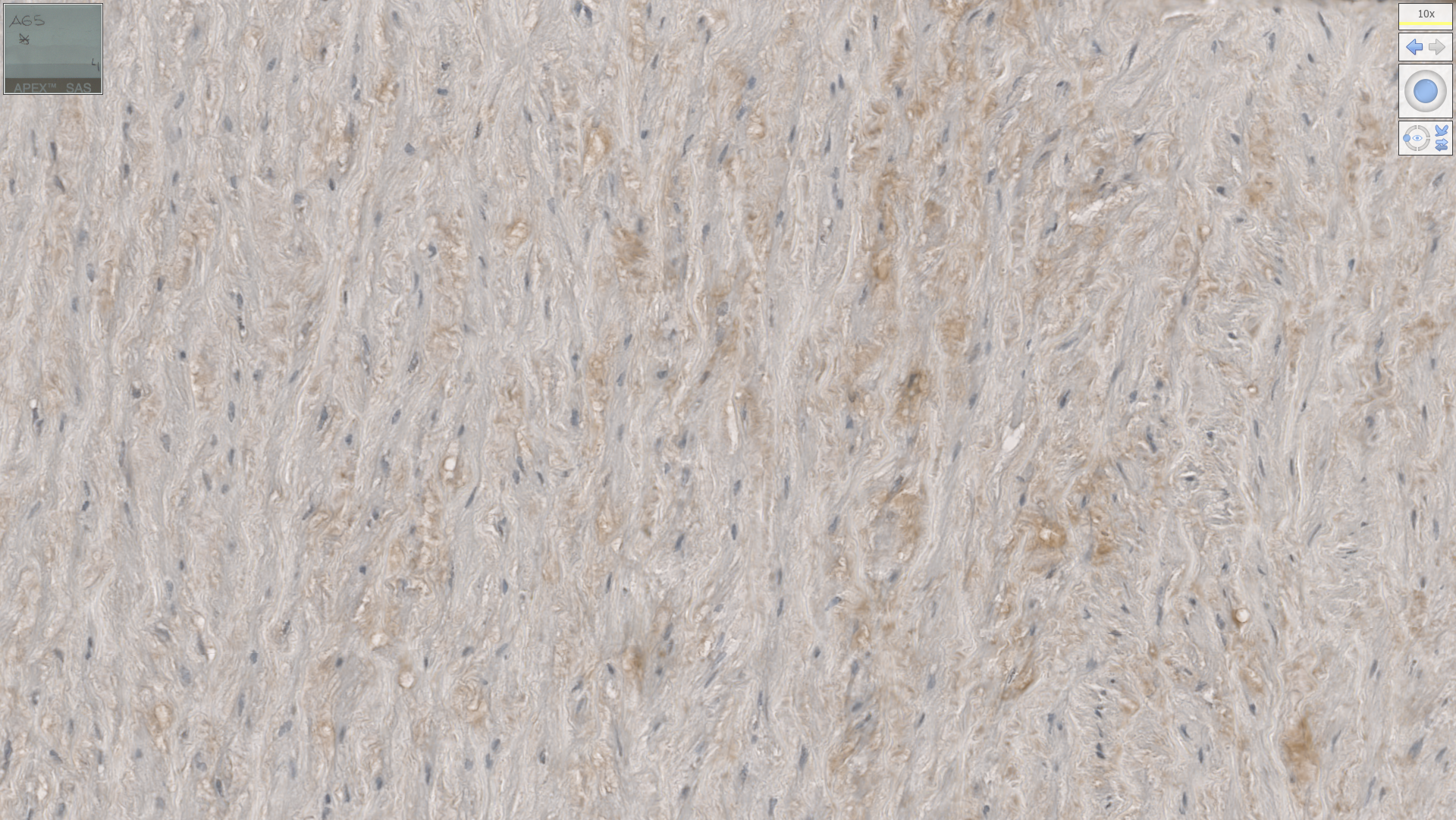

Supplement: Supplementary file 11 — Source Data EV Figures [file 44321_2023_9_MOESM11_ESM.zip › Figure EV2/VCAN-MFS-a65_2.tif]

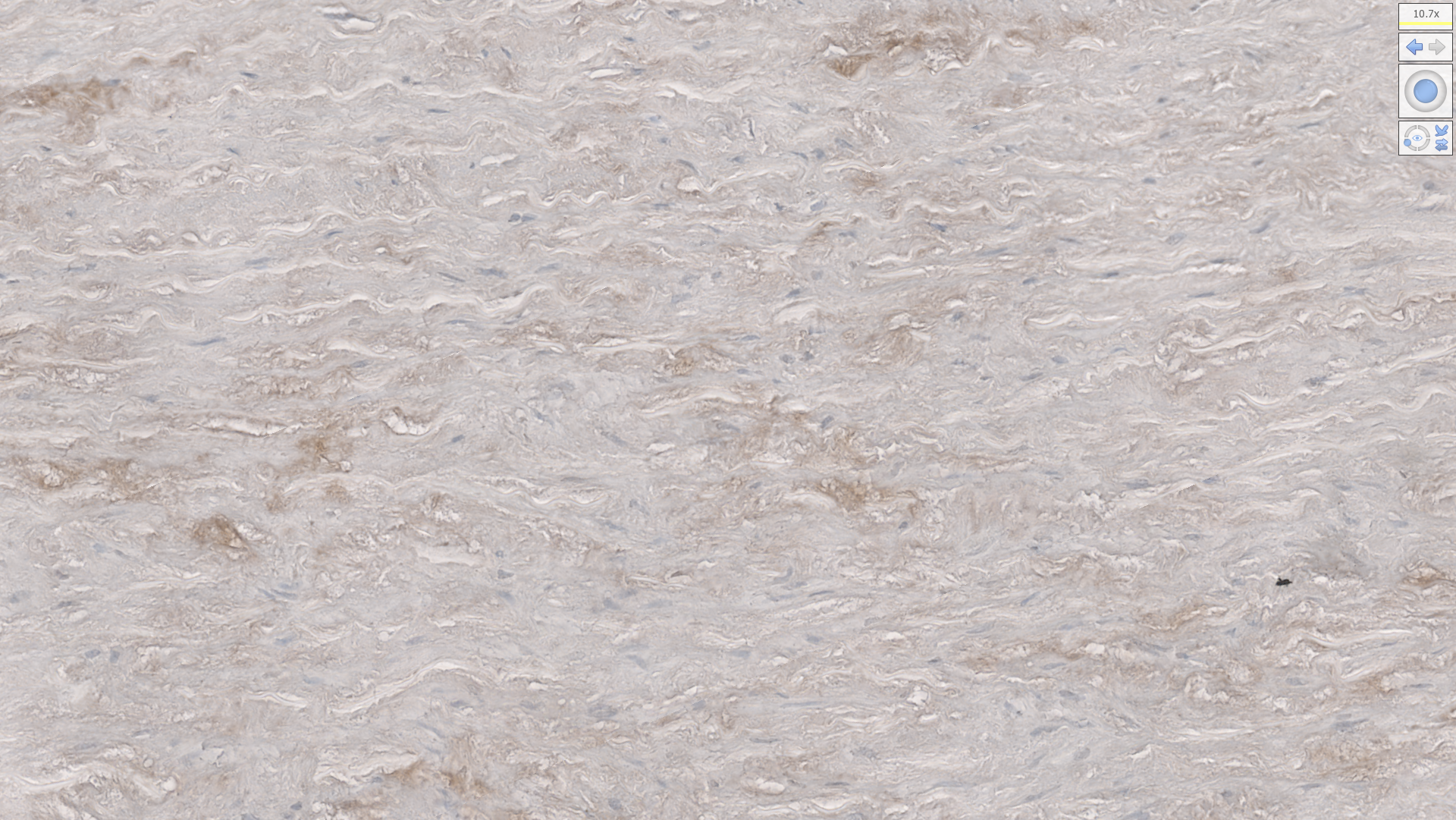

Supplement: Supplementary file 11 — Source Data EV Figures [file 44321_2023_9_MOESM11_ESM.zip › Figure EV2/VCAN-MFS-ao26_2.tif]

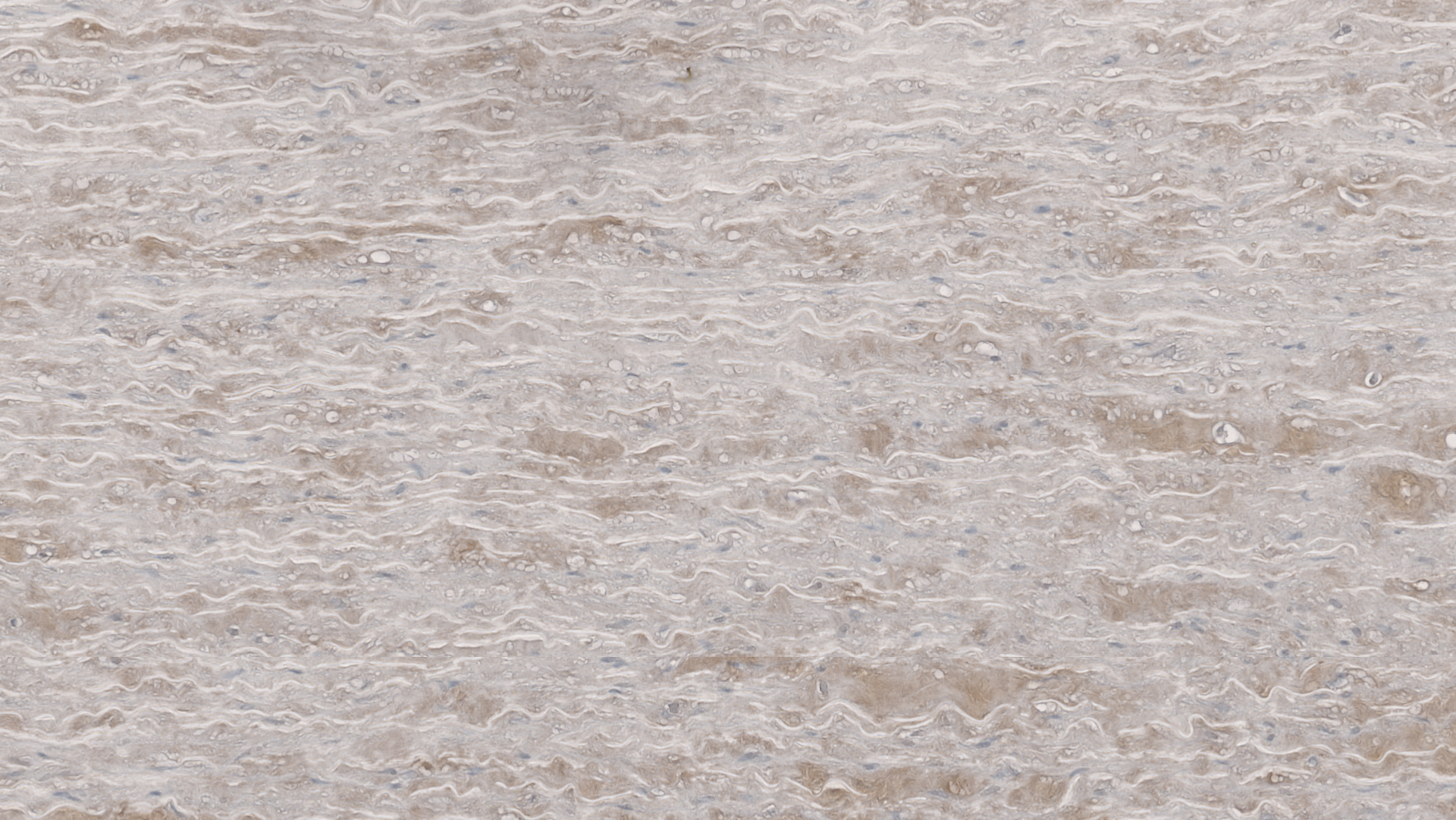

Supplement: Supplementary file 11 — Source Data EV Figures [file 44321_2023_9_MOESM11_ESM.zip › Figure EV2/VCAN-MFS-ao41_2.tif]

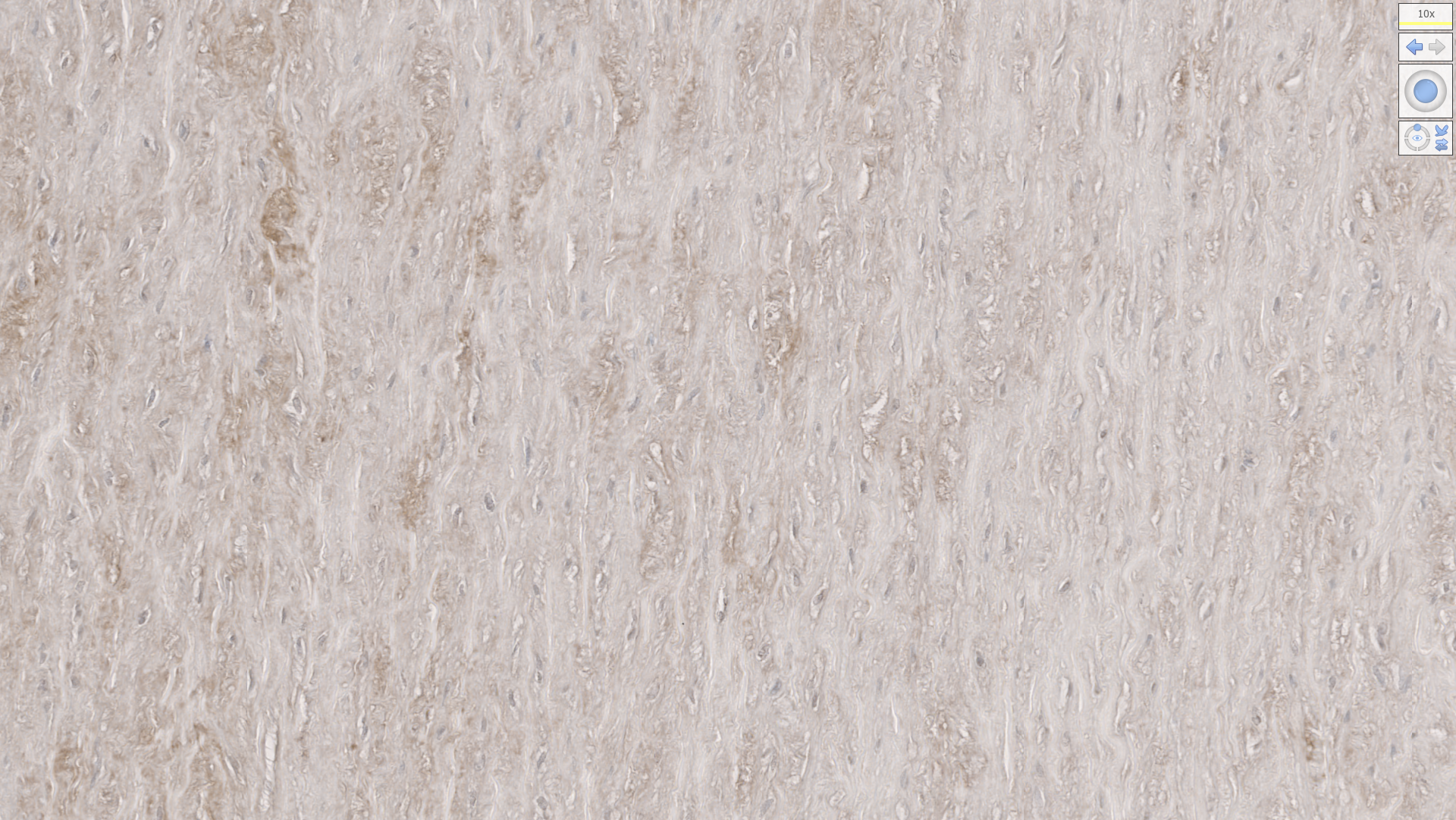

Supplement: Supplementary file 11 — Source Data EV Figures [file 44321_2023_9_MOESM11_ESM.zip › Figure EV2/VCAN-MFS-ao7_3.tif]
