# Supplementary material for: Versican accumulation drives Nos2 induction and aortic disease in Marfan syndrome via Akt activation
Source: EMBO Mol Med. 2024 Jan 2;16(1):9. doi: 10.1038/s44321-023-00009-7 (PMC10897446; doi:10.1038/s44321-023-00009-7)
Supplement: Supplementary file 12 — Expanded View Figures [file 44321_2023_9_MOESM12_ESM.pdf]

Expanded View Figures

Figure EV1. Accumulation of Vcan in aortas of MFS mice at 12 weeks but not at 4 weeks of age.

(**A**) Representative images of Vcan immunofluorescence (red), elastin autofluorescence (green), and Hoechst-stained nuclei (blue) in aortic sections from 4- or 12-week-old WT and MFS mice. Scale bar, 50 µm. (**B**) Quantification of Vcan immunofluorescence in mouse AsAo (4-week-old: WT, $n = 4$; MFS, $n = 3$. 12-week-old: WT, $n = 4$; MFS, $n = 3$). Data information: (**B**) data are shown relative to 12-week-old WT mice as mean ± s.e.m. Each data point denotes an individual mouse. $^*P < 0.05$ (two-way ANOVA with Tukey's post hoc test). Source data are available online for this figure.

© This is a U.S. Government work and not under copyright protection in the US; foreign copyright protection may apply

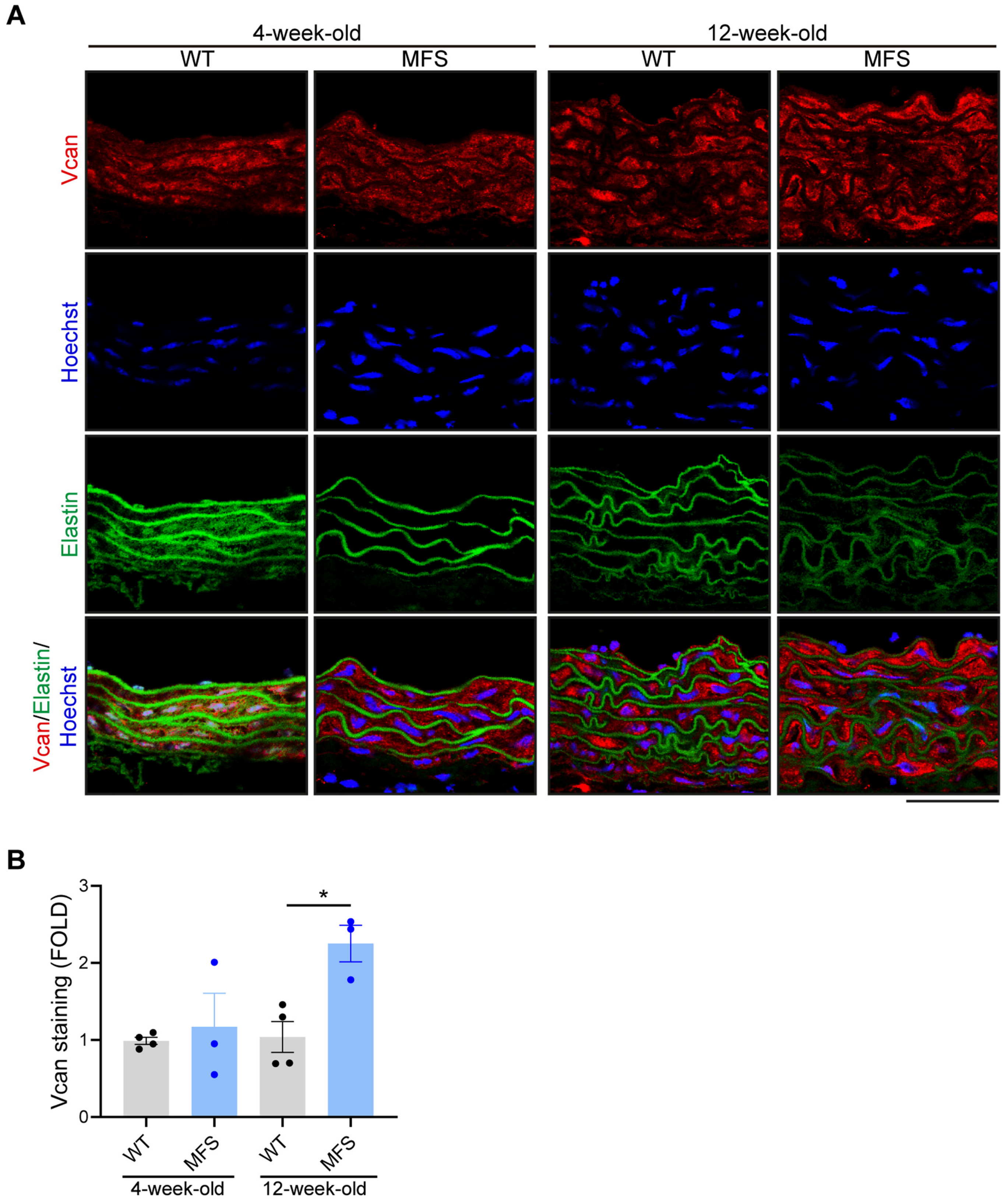

© This is a U.S. Government work and not under copyright protection in the US; foreign copyright protection may apply

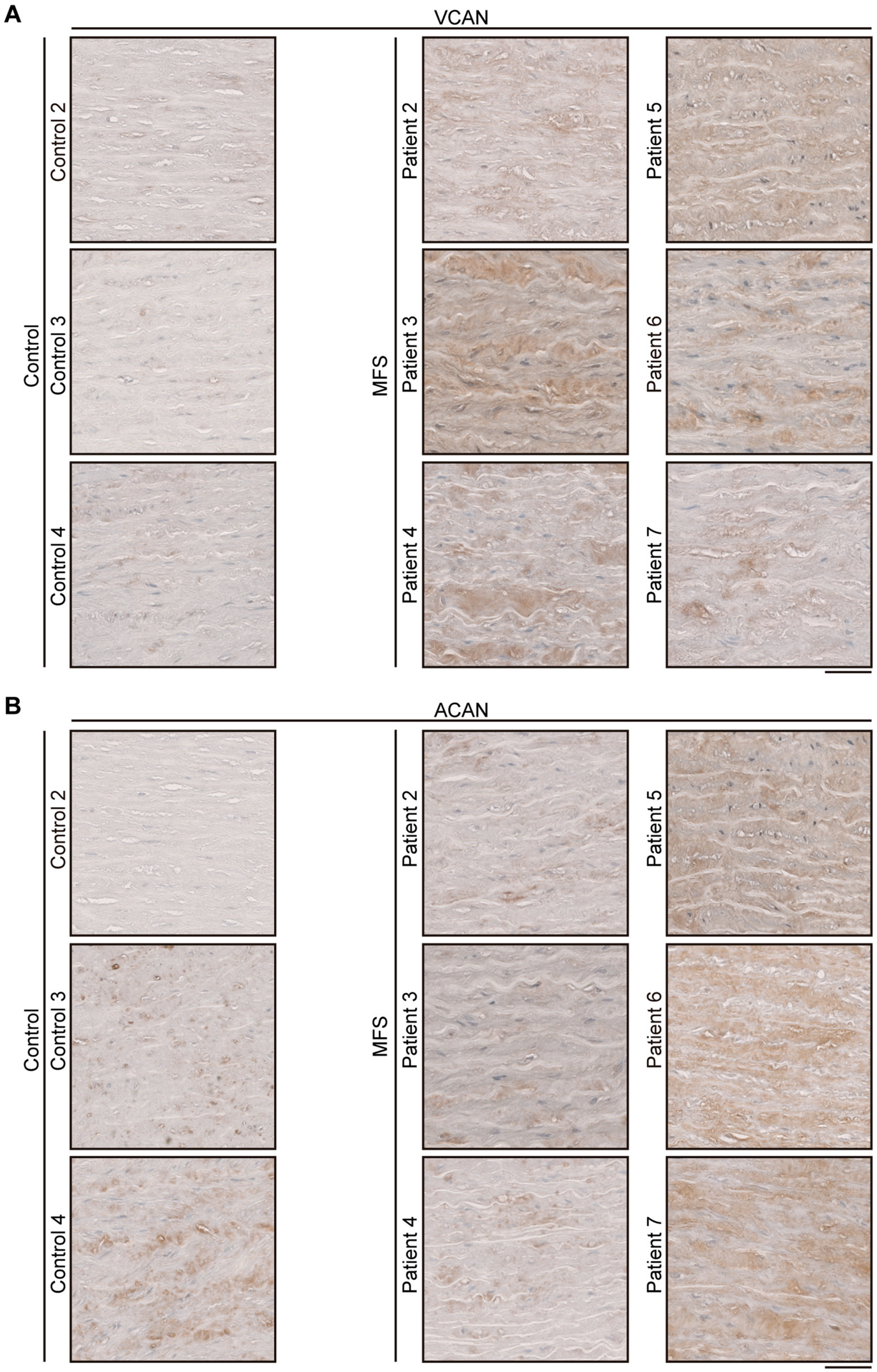

© This is a U.S. Government work and not under copyright protection in the US; foreign copyright protection may apply

◀ **Figure EV2. VCAN and ACAN protein expression in aortas of MFS patients.**

(**A**, **B**) Representative images of (**A**) VCAN and (**B**) ACAN in the medial layer of aortic sections from 3 control donors and 6 MFS patients. Scale bar, 50 μm. Source data are available online for this figure.

 © This is a U.S. Government work and not under copyright protection in the US; foreign copyright protection may apply

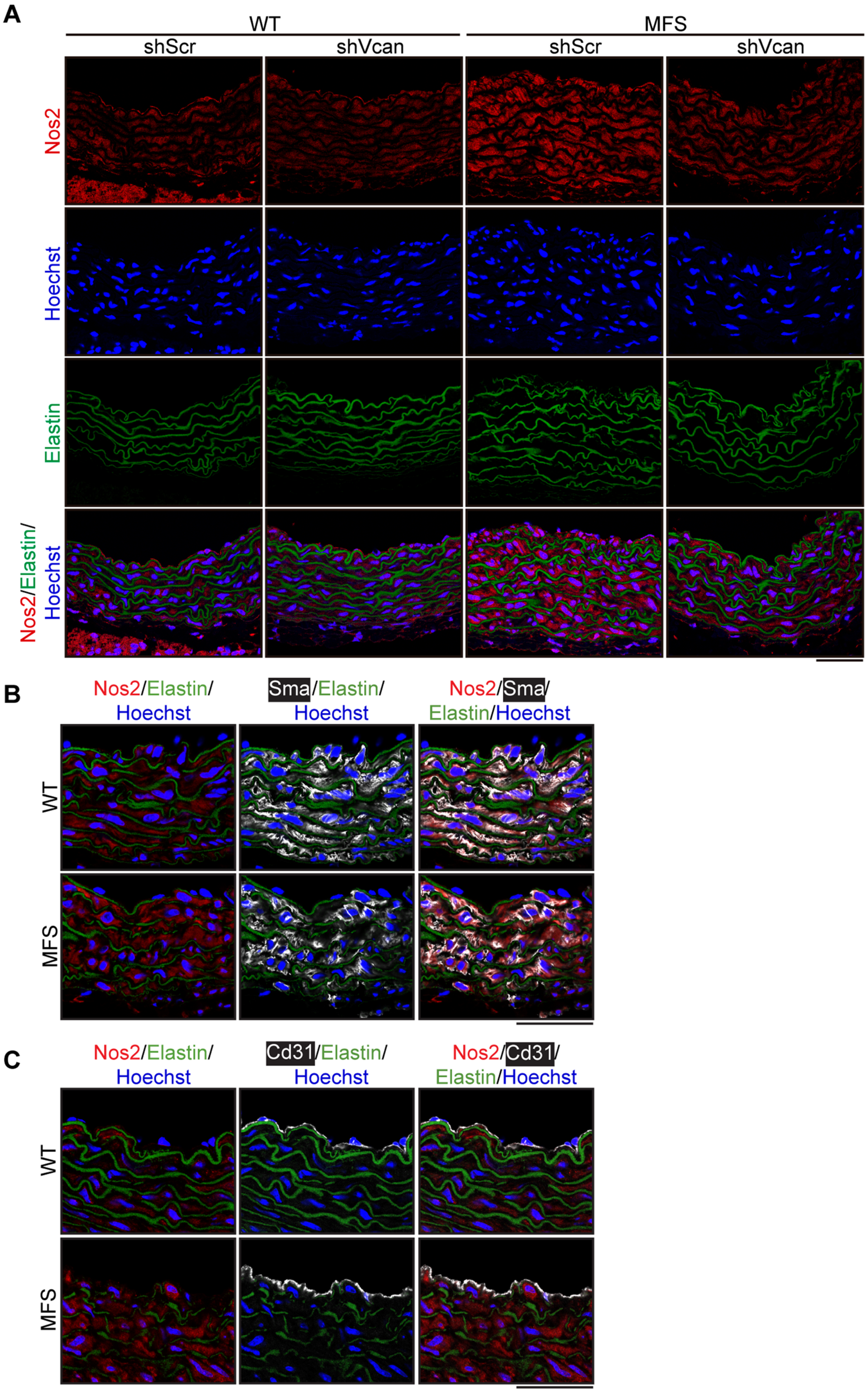

© This is a U.S. Government work and not under copyright protection in the US; foreign copyright protection may apply

Figure EV3. ***Vcan* silencing reduces Nos2 expression in aortas from MFS mice.**

(**A**) Representative images of Nos2 immunofluorescence (red), Hoechst-stained nuclei (blue), and elastin autofluorescence (green) in aortic sections from 16-week-old MFS and WT mice. Individual channels are shown followed by a composite image. Scale bar, 50 μm. The 4 merged images are identical to those shown in Fig. 7D. (**B**, **C**) Representative images of (**B**) Sma or (**C**) Cd31 (pale gray), Nos2 immunofluorescence (red), elastin autofluorescence (green), and Hoechst-stained nuclei (blue) in aortic sections from 12-week-old WT and MFS mice. Scale bar, 50 μm. Source data are available online for this figure.

 © This is a U.S. Government work and not under copyright protection in the US; foreign copyright protection may apply

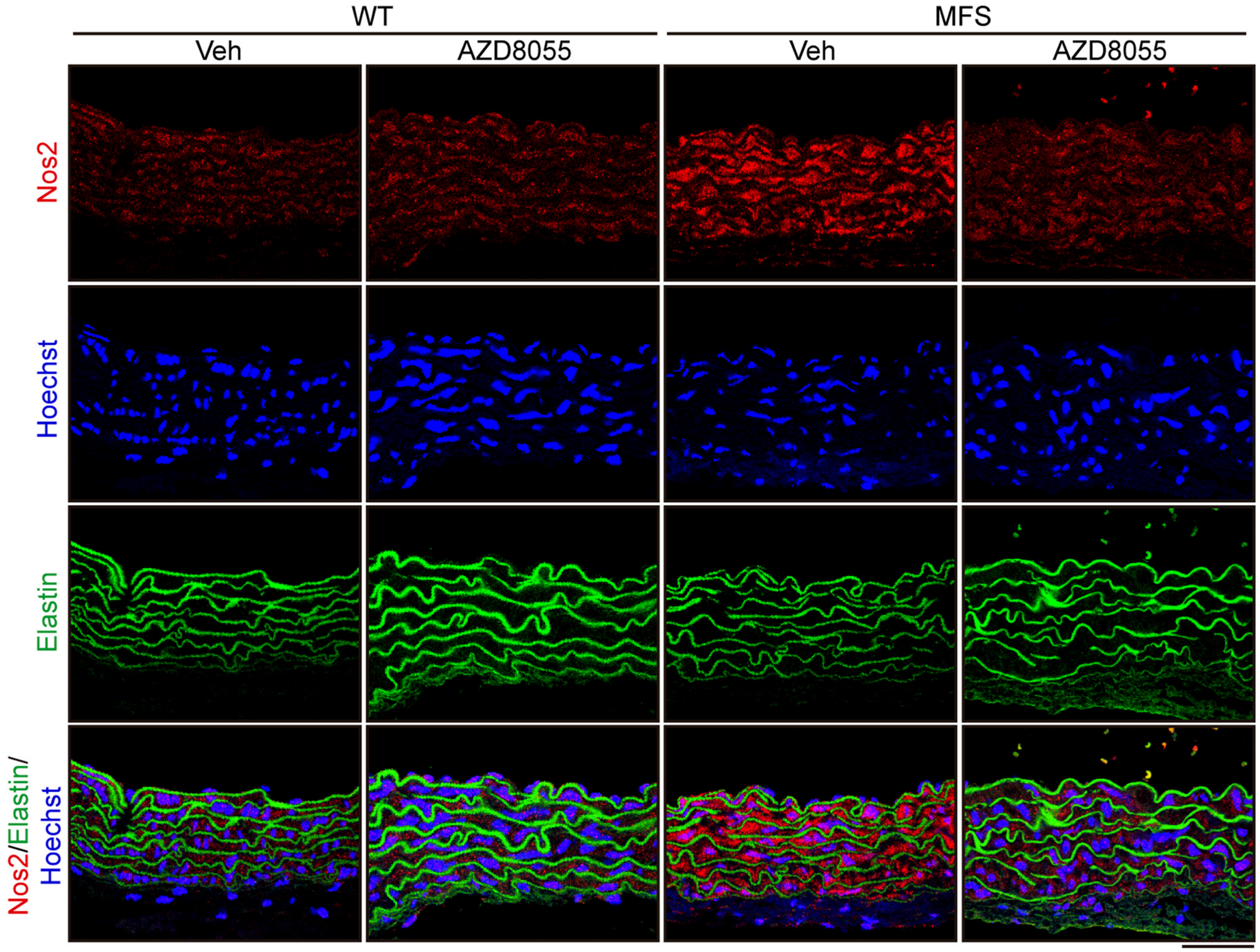

Figure EV4. Pharmacological inhibition of Akt signaling decreases aortic Nos2 expression in MFS mice.

Representative images of Nos2 immunofluorescence (red), Hoechst-stained nuclei (blue), and elastin autofluorescence (green), in aortic sections from WT and MFS mice treated as indicated. Individual channels are shown followed by a composite image. Scale bar, 50 μm. The 4 merged images are identical to those shown in Fig. 8D. Source data are available online for this figure.

© This is a U.S. Government work and not under copyright protection in the US; foreign copyright protection may apply

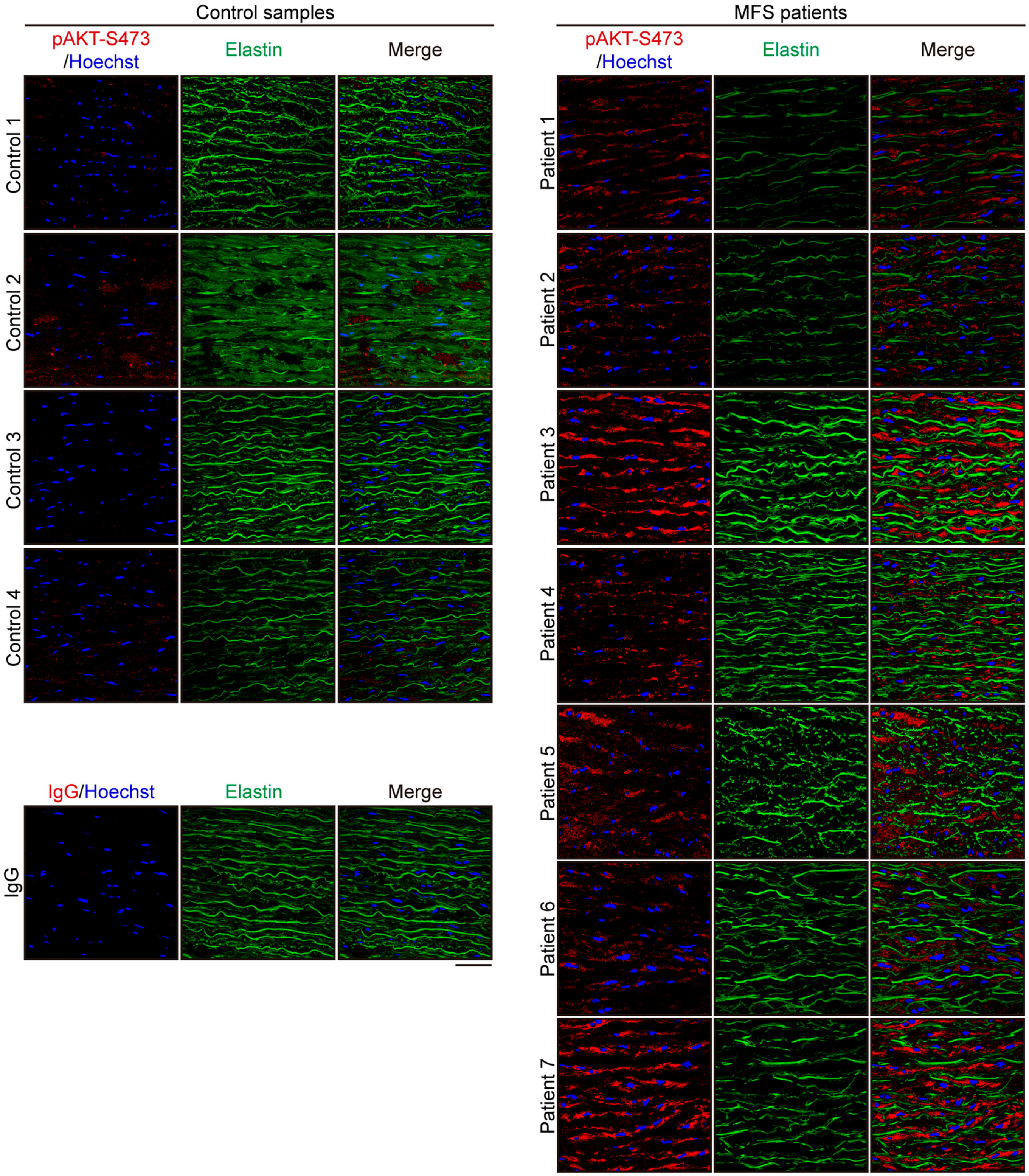

Figure EV5. AKT is activated in the aortas of MFS patients.

Representative images of pAKT-S473 immunofluorescence (red), Hoechst-stained nuclei (blue), and elastin autofluorescence (green) in the medial layer of aortic sections from 4 control donors and 7 MFS patients. A representative image of the staining with a control IgG (red) is also shown. Scale bar, 50 μm. The pAKT-S473/Hoechst and merge images corresponding to Control 1 and Patient 1 are identical to the pAKT-S473/Hoechst and pAKT-S473/Elastin/Hoechst images shown in Fig. 8F. Source data are available online for this figure.

© This is a U.S. Government work and not under copyright protection in the US; foreign copyright protection may apply
